# Development of novel anilinoquinazoline-based carboxylic acids as non-classical carbonic anhydrase IX and XII inhibitors

**DOI:** 10.1080/14756366.2023.2191163

**Published:** 2023-03-21

**Authors:** Zainab M. Elsayed, Hadia Almahli, Alessio Nocentini, Andrea Ammara, Claudiu T. Supuran, Wagdy M. Eldehna, Sahar M. Abou-Seri

**Affiliations:** a Scientific Research and Innovation Support Unit, Kafrelsheikh University, Kafrelsheikh, Egypt; b Department of Chemistry, University of Cambridge, Cambridge, UK; c Department of NEUROFARBA, Section of Pharmaceutical and Nutraceutical Sciences, University of Florence, Firenze, Italy; d Department of Pharmaceutical Chemistry, Kafrelsheikh University, Kafrelsheikh, Egypt; e School of Biotechnology, Badr University in Cairo, Badr City, Cairo, Egypt; f Department of Pharmaceutical Chemistry, Cairo University, Cairo, Egypt

**Keywords:** 2-Arylquinazoline, anticancer agents, metalloenzymes, drug design

## Abstract

As part of our ongoing endeavour to identify novel inhibitors of cancer-associated CA isoforms IX and XII as possible anticancer candidates, here we describe the design and synthesis of small library of 2-aryl-quinazolin-4-yl aminobenzoic acid derivatives (**6a–c**, **7a–c**, and **8a–c**) as new non-classical CA inhibitors. On account of its significance in the anticancer drug discovery and in the development of effective CAIs, the 4-anilinoquinazoline privileged scaffold was exploited in this study. Thereafter, the free carboxylic acid functionality was appended in the *ortho* (**6a–c**), *meta* (**7a–c**), or *para*-positon (**8a–c**) of the anilino motif to furnish the target inhibitors. All compounds were assessed for their inhibitory activities against the hCA I, II (cytosolic), IX, and XII (trans-membrane, tumour-associated) isoforms. Moreover, six quinazolines (**6a–c**, **7b**, and **8a–b**) were chosen by the NCI-USA for *in vitro* anti-proliferative activity evaluation against 59 human cancer cell lines representing nine tumour subpanels.

## Introduction

Carbonic anhydrases (CA, EC 4.2.1.1) are ubiquitous metalloenzymes that play a crucial role in catalysing the reversible hydration reaction of carbon dioxide to bicarbonate and protons.^1^ This reaction, catalysed by Zn^+2^ ion, has a critical role in many physiological and pathological processes such as gluconeogenesis and tumorigenicity.^2^^,^^3^ So far, fifteen human CA (*h*CA) isoforms have been identified, with varying distributions across tissues and cells.^4^ As a result of the dysfunction of different *h*CA isoforms activities, a number of pathological repercussions might occur, featuring these *h*CA isoforms as interesting pharmacological targets for a variety of therapeutic approaches using small molecule CA inhibitors (CAIs).^4^ Thus, the pharmacological applications of CAIs are identified for the management of diverse disorders such as ophthalmologic problems,^5^ epilepsy,^6^ obesity^7^ and human malignancies.^8^

**Figure 1.**
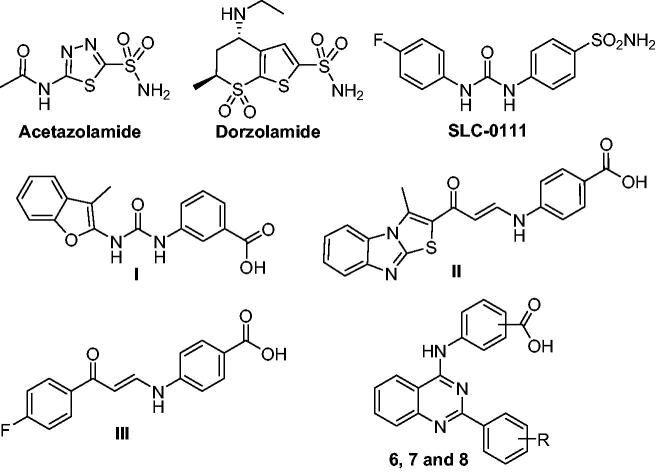
Structure of acetazolamide, dorzolamide, SLC-0111, non-classical CAIs (**I–III**), and the target inhibitors (**6**, **7,** and **8**).

Sulphonamides and their sulfamides and sulfamate bioisosteres are considered as classical hCA inhibitors with a high affinity to the zinc ion in the active site.^3^ It is worth to mention that although identification of several chemotypes of CAIs, like coumarins, phenols, thiocarbamates, and carboxylates,^9–11^ only primary sulfonamide-tethered CAIs have been clinically used for glaucoma (such as acetazolamide and dorzolamide), and investigated in the clinical trials for the treatment of human malignancies (SLC-0111), Figure 1.^12^^,^^13^ These sulfonamide-tethered CAIs produce strong CA inhibition, however, a number of them lack the necessary isoform selectivity. So, the design and synthesis of new non­classical CAIs stands out as a promising strategy to discover effective and isoform-selective CAIs for the management of different diseases. The carboxylic acid-based derivatives represent an important non-classical CAIs chemotype that can exert the CA inhibitory effect through different modes of action, such as anchoring to the zinc-bound water-hydroxide ion through H-bonding, or direct binding to the catalytic zinc displacing bound water-hydroxide anion.^14–16^

In the few last years, we have reported several carboxylic acid-tethered small molecules as new CAIs.^17–19^ A novel series of benzofuran-based carboxylic acids was described as promising CA inhibitors in 2020.^20^ Among these benzofuran derivatives, compound **I** (Figure 1) with a *meta*-benzoic acid moiety inhibited *h*CA IX at a submicromolar concentration (*K*_I_ = 0.79 μM), as well as exerted good *h*CA XII inhibitory activity (*K*_I_ = 2.3 μM). Also in the same year, we have developed a small library of methylthiazolo[3,2-*a*]benzimidazole-based carboxylic acid derivatives as novel CA inhibitors.^21^ In particular, compound **II** (Figure 1) effectively suppressed CA isoforms IX and XII with inhibition constants equal 0.83 μM and 2.4 μM, respectively. Furthermore, we identified a new series of non-classical CA inhibitors that incorporates enaminone-based carboxylic acids.^22^ Compound **III** (Figure 1) endowed with a *para*-benzoic acid motif showed submicromolar *h*CA IX inhibitory activity (*K*_I_ = 0.92 µM) and good *h*CA XII inhibitory activity (*K*_I_ = 1.1 µM).

Based on the findings described above, and as part of our ongoing endeavour to identify novel inhibitors of cancer-associated CA isoforms IX and XII as possible anticancer candidates,^23–29^ here we describe the design and synthesis of a small library of 2-aryl-quinazolin-4-yl aminobenzoic acid derivatives (**6a–c**, **7a–c**, and **8a–c**) as new non-classical CA inhibitors (Figure 1). On account of its significance in the anticancer drug discovery and development,^30–33^ and in the development of effective CAIs,^34^^,^^35^ the 4-anilinoquinazoline privileged scaffold was exploited in this study. Thereafter, the free carboxylic acid functionality was appended in the *ortho* (**6a–c**), *meta* (**7a–c**), or *para*-positon (**8a–c**) of the anilino motif to furnish the target inhibitors.

All the newly synthesised quinazoline-based carboxylic acid derivatives (**6a–c**, **7a–c,** and **8a–c**) were assessed for their inhibitory activities against the hCA I, II (cytosolic), IX and XII (trans membrane, tumour associated) isoforms by the stopped-flow CO_2_ hydrase assay. Moreover, six quinazolines (**6a–c**, **7b**, and **8a–b**) were chosen by the NCI-USA for *in vitro* anti-proliferative activity evaluation against 59 human cancer cell lines representing nine tumour subpanels.

## Experimental

### Chemistry

Melting points (°C, uncorrected) were determined using a Stuart melting point apparatus. The IR spectra (KBr) were recorded on a SHIMADZU FT/IR spectrometer. The NMR spectra recorded by BRUKER 400 MHz NMR spectrometers using DMSO-*d_6_* as the solvent. Chemical shifts were reported in parts per million (*δ*), and coupling constants (*J*) expressed in Hertz. ^1^H and ^13^C spectra were run at 400 and 101 MHz, respectively. Microanalytical data (C, H, and N) were obtained by FLASH 2000 CHNS/O analyser.

#### General procedures for the synthesis of 2-arylquinazolin-4(3H)-one derivatives (3a–c)

An aqueous solution of ferric chloride (5.4 g, 20 mmol) was added to a mixture of anthranilamide **1** (2.72 g, 20 mmol) and the appropriate aldehyde derivative **2a–c** (20 mmol).^36^ The mixture was heated at 80 °C for 3 h. After completion of the reaction, as indicated by TLC (n-hexane: ethyl acetate 1:1), the formed solid was filtrated, washed with water (4 × 5 ml), dried, and finally recrystallized from dioxane to produce 2-arylquinazolin-4(3*H*)-ones **3a–c**.

#### General procedures for the synthesis of 2-aryl-4-chloroquinazolines (4a–c)

To a suspension of 2-arylquinazolinones **3a–c** (1eq) in phosphorus oxychloride (10 eq), a catalytic amount of DMF was added.^36^ The reaction mixture was then heated at 90 °C for 4h. After cooling, the mixture was added drop-wise to ice-water with stirring, neutralised by ammonium hydroxide, and extracted by methylene chloride. The organic layer was washed with cold water, dried over anhydrous Na_2_SO_4_, and evaporated *in vacuo*. The obtained solid was crystallised from isopropanol to afford the key 2-aryl-4-chloroquinazolines intermediates **4a–c**.

#### Synthesis of 2/3/4-((2-arylquinazolin-4-yl)amino)benzoic acid derivatives (6a–c, 7a–c and 8a–c)

To a stirred solution of 4-chloroquinazoline derivatives **4a–c** (1 mmol) in refluxing isopropanol (5 ml) containing a few drops of HCl, the appropriate aminobenzoic acid derivative **5a–c** (1 mmol) was added. The reaction mixture was heated under reflux for 2 h. The solid formed upon cooling was collected by filtration, dried, and recrystallized from ethanol to afford the target quinazolines (**6a–c**, **7a–c,** and **8a–c**).

##### 2-((2-(m-Tolyl)quinazolin-4-yl)amino)benzoic acid (6a)

White crystals, (67%) yield; m.p. 198–200 °C; IR (KBr) *ν*_max_/cm^−1^; ^1^H NMR (DMSO-*d*_6_): 2.42 (s, 3H, CH_3_), 7.47–7.51 (m, 3H, Ar-H), 7.80 (t, 1H, H-6 quinazoline, *J* = 7.2 Hz), 7.86 (t, 1H, H-7 quinazoline, *J* = 7.6 Hz), 8.09–8.16 (m, 3H, Ar-H), 8.24–8.26 (m, 2H, Ar-H), 8.38 (d, 1H, H-5, quinazoline, *J* = 8.4 Hz), 8.61 (d, 1H, H-8 quinazoline, *J* = 8.0 Hz), 12.34 (s, 1H, NH); ^13^C NMR (DMSO-*d*_6_): 21.47 (CH_3_), 113.31, 122.0, 124.02, 125.95, 126.68, 126.85, 128.93, 129.36, 130.10, 131.54, 132.34, 133.78, 134.24, 136.35, 138.06, 138.74, 157.99, 159.29, 168.84 (C=O); Anal. Calcd. For: C_22_H_17_N_3_O_2_ (355.40): C, 74.35; H, 4.82; N, 11.82; Found: C, 74.15; H, 4.79; N, 11.85.

##### 2-((2-(p-Tolyl)quinazolin-4-yl)amino)benzoic acid (6b)

White crystals, (65%) yield; m.p. 200–202 °C; IR (KBr) *ν*_max_/cm^−1^; ^1^H NMR (DMSO-*d*_6_): 2.41 (s, 3H, CH_3_), 7.41 (d, 2H, Ar-H, *J* = 8.0 Hz_)_ , 7.47 (t, 1H, Ar-H, *J* = 7.2 Hz), 7.79 (t, 1H, H-6 quinazoline, *J* = 7.6 Hz), 7.85 (t, 1H, Ar-H, *J* = 7.6 Hz), 8.08–8.13 (m, 2H, Ar-H), 8.23–8.25 (m, 1H, Ar-H) , 8.28 (d, 2H, Ar-H, *J* = 8.4 Hz), 8.41 (d, 1H, H-5 quinazoline, *J* = 7.6 Hz), 8.61 (d, 1H,H-8 quinazoline, *J* = 8.4 Hz), 12.34 (s, 1H, NH); ^13^C NMR (DMSO-*d*_6_): 21.67 (CH_3_), 113.21, 124.08, 126.03, 126.77, 128.85, 129.71, 130.04, 131.53, 133.79, 136.39, 144.36, 157.78, 159.32, 168.80 (C=O); Anal. Calcd. For: C_22_H_17_N_3_O_2_ (355.40): C, 74.35; H, 4.82; N, 11.82; Found: 74.25; H, 4.80; N, 11.84.

##### 2-((2–(4-Methoxyphenyl)quinazolin-4-yl)amino)benzoic acid (6c)

White crystals, (72%) yield; m.p. 204–206 °C; IR (KBr) *ν*_max_/cm^−1^; ^1^H NMR (DMSO-*d*_6_): 3.87 (s, 3H, OCH_3_), 7.15 (d, 2H, Ar-H, *J* = 8.8 Hz), 7.49 (t, 1H, Ar-H, *J* = 7.2 Hz), 7.79 (t, 1H, H-6 quinazoline, *J* = 8.8 Hz), 7.83 (t, 1H, Ar-H, *J* = 8.8 Hz), 8.08–8.20 (m, 3H, Ar-H), 8.39 (d, 2H, Ar-H, *J* = 9.2 Hz), 8.45 (d, 1H, H-5 quinazoline, *J* = 8.4 Hz), 8.62 (d, 1H, H-8 quinazoline, *J* = 8.0 Hz), 12.28 (s, 1H, NH); ^13^C NMR (DMSO-*d*_6_): 56.02 (OCH_3_), 56.25, 114.56, 115.03, 120.92, 126.45, 126.99, 130.34, 131.34, 131.84, 133.75, 135.30, 136.57, 159.33, 162.52, 162.70; Anal. Calcd. For: C_22_H_17_N_3_O_3_ (371.40): C, 71.15; H, 4.61; N, 11.31; Found: 71.37; H, 4.60; N, 11.25.

##### 3-((2-(m-Tolyl)quinazolin-4-yl)amino)benzoic acid (7a)

Yellow crystals, (72%) yield; m.p. 248–250 °C; IR (KBr) *ν*_max_/cm^−1^; ^1^H NMR (DMSO-*d*_6_):2.42 (s, 3H, CH_3_), 7.47–7.53 (m, 2H, Ar-H), 7.63 (t, 1H, H-6 quinazoline, *J* = 8.0 Hz), 7.80 (t, 1H, H7 quinazoline, *J* = 7.6 Hz), 7.89 (d, 1H, Ar-H, *J* = 7.6 Hz), 8.07–8.11 (m, 2H, Ar-H), 8.30–8.31 (m, 2H, Ar-H) 8.40 (d, 1H, H-5 quinazoline, *J* = 8.4 Hz), 8.75 (s, 1H, Ar-H), 9.03 (d, 1H, H-8 quinazoline, *J* = 8.0 Hz),11.82 (s, 1H, NH); ^13^C NMR (DMSO-*d*_6_): 21.41 (CH_3_), 25.96, 62.49, 113.26, 125.15, 125.52, 126.96, 127.27, 128.57, 129.31, 130.34, 131.64, 134.48, 136.38, 137.81, 138.96, 157.61, 159.31, 167.49 (C=O); Anal. Calcd. For: C_22_H_17_N_3_O_2_ (355.40): C, 74.35; H, 4.82; N, 11.82; Found: 74.38; H, 4.85; N, 11.78.

##### 3-((2-(p-Tolyl)quinazolin-4-yl)amino)benzoic acid (7b)

Yellow crystals, (67%) yield; m.p. 220–223 °C; IR (KBr) *ν*_max_/cm^−1^; ^1^H NMR (DMSO-*d*_6_):2.42 (s, 3H, CH_3_), 7.40 (d, 2H, Ar-H, *J* = 8.0 Hz), 7.64 (t, 1H, Ar-H, *J* = 7.6 Hz), 7.80 (t, 1H, H-6 quinazoline, *J* = 7.6 Hz), 7.89 (d, 1H, Ar-H, *J* = 7.6 Hz), 8.07 (t, 1H, H-7 quinazoline, *J* = 8.0 Hz), 8.12 (d, 1H, Ar-H, *J* = 8.0 Hz), 8.37 (d, 2H, Ar-H, *J* = 8.0 Hz), 8.40 (d, 1H, H-5 quinazoline, *J* = 8.8 Hz), 8.69 (s, 1H, H-2, Ar-H), 9.00 (d, 1H, H-8 quinazoline, *J* = 8.4 Hz), 11.79 (s, 1H, NH); ^13^C NMR (DMSO-*d*_6_): 21.69, 25.97, 62.48, 113.20, 120.96, 125.15, 125.51, 127.28, 128.49, 128.73, 129.44, 129.87, 130.05, 131.70, 136.38, 137.82, 144.55, 157.46, 159.35, 167.41; Anal. Calcd. For: C_22_H_17_N_3_O_2_ (355.40): C, 74.35; H, 4.82; N, 11.82; Found: 74.51; H, 4.79; N, 11.86.

##### 3-((2–(4-Methoxyphenyl)quinazolin-4-yl)amino)benzoic acid (7c)

Off white crystals, (75%) yield; m.p. 244–246 °C; IR (KBr) *ν*_max_/cm^−1^; ^1^H NMR (DMSO-*d*_6_): 3.90 (s, 3H, OCH_3_), 7.18 (d, 2H, Ar-H, *J* = 8.8 Hz), 7.66 (t, 1H, H-6 quinazoline, *J* = 8.0 Hz), 7.77 (d, 1H, Ar-H, *J =* 7.2 Hz), 7.81 (t, 1H, H-7 quinazoline, *J* = 8.0 Hz), 7.91 (d, 1H, Ar-H, *J* = 7.6 Hz), 8.10 (d, 1H, Ar-H, *J* = 8.0 Hz), 8.37 (d, 1H, H-5 quinazoline, *J* = 8.4 Hz), 8.48 (d, 2H, Ar-H, *J* = 8.8 Hz), 8.69 (s, 1H, H-2, Ar-H), 8.93(d, 1H, H-8 quinazoline, *J* = 8.4 Hz), 11.70 (s, 1H, NH); ^13^C NMR (DMSO-*d*_6_): 56.25 (OCH_3_), 113.02, 115.00, 123.62, 125.06, 125.53, 125.99, 127.35, 128.36, 128.79, 129.54, 130.40, 131.76, 132.06, 132.49, 136.47, 137.82, 157.04, 159.28, 164.16, 167.07, 167.42; Anal. Calcd. For: C_22_H_17_N_3_O_3_ (371.40): C, 71.15; H, 4.61; N, 11.31; Found: C, 71.27; H, 4.64; N, 11.26.

##### 4-((2-(m-Tolyl)quinazolin-4-yl)amino)benzoic acid (8a)

White crystals, (78%) yield; m.p. 201–204 °C; IR (KBr) *ν*_max_/cm^−1^; ^1^H NMR (DMSO-*d*_6_):2.43 (s, 3H, CH_3_), 7.52–7.53 (m, 2H, Ar-H), 7.81 (t, 1H, H-6 quinazoline, *J* = 7.2 Hz), 8.05–8.12 (m, 5H, Ar-H), 8.20–8.22 (m, 1H, Ar-H) , 8.28 (s, 1H, Ar-H), 8.39 (d, 1H, H-5 quinazoline, *J* = 8.4 Hz), 9.01 (d, 1H, H-8 quinazoline, *J* = 8.0 Hz), 11.79 (s, 1H, NH); ^13^C NMR (DMSO-*d*_6_): 21.50 (CH_3_), 113.39, 124.27, 125.17, 127.03, 128.33, 128.59, 129.48, 130.11, 130.37, 132.13, 134.38, 136.43, 138.84, 141.66, 157.78, 159.42, 167.30 (C=O); Anal. Calcd. For: C_22_H_17_N_3_O_2_ (355.40): C, 74.35; H, 4.82; N, 11.82; Found: C, 74.32; H, 4.81; N, 11.86.

##### 4-((2-(p-Tolyl)quinazolin-4-yl)amino)benzoic acid (8b)

Yellow crystals, (70%) yield; m.p. 306–308 °C; IR (KBr) *ν*_max_/cm^−1^; ^1^H NMR (DMSO-*d*_6_):2.43 (s, 3H, CH_3_), 7.46 (d, 2H, Ar-H, *J* = 8.0 Hz), 7.82 (t, 1H, H-6 quinazoline, *J* = 8.0 Hz), 8.04 (d, 2H, Ar-H, *J* = 8.8 Hz), 8.09 (d, 2H, Ar-H, *J* = 4 Hz_)_ , 8.11 (t, 1H, H-7 quinazoline, *J* = 2.8 Hz), 8.32 (d, 2H, Ar-H, *J* = 8.4 Hz), 8.36 (d, 1H, H-5 quinazoline, *J* = 8.4 Hz), 8.96 (d, 1H, H-8 quinazoline, *J* = 8.0 Hz) 11.72 (s, 1H, NH); ^13^C NMR (DMSO-*d*_6_): 21.70, 113.35, 124.26, 125.10, 128.32, 128.51, 129.75, 130.18, 130.44, 136.45, 141.67, 144.41, 157.70, 159.44, 167.30; Anal. Calcd. For: C_22_H_17_N_3_O_2_ (355.40): C, 74.35; H, 4.82; N, 11.82; Found: C, 74.21; H, 4.80; N, 11.84.

##### 4-((2–(4-Methoxyphenyl)quinazolin-4-yl)amino)benzoic acid (8c)

Off white crystals, (78%) yield; m.p. >300 °C; IR (KBr) *ν*_max_/cm^−1^; ^1^H NMR (DMSO-*d*_6_): 3.90 (s, 3H, OCH_3_), 7.22 (d, 2H, Ar-H*, J* = 8.8 Hz,), 7.71 (d, 1H, Ar-H, *J* = 8.8 Hz), 7.81 (t, 1H, H-6 quinazoline, *J* = 7.6 Hz), 8.01 (d, 2H, Ar-H, *J* = 8.4 Hz), 8.09–8.13 (m, 3H, Ar-H and H-7 quinazoline), 8.42–8.47 (m, 3H, Ar-H and H-5 quinazoline), 8.97 (d, 1H, H-8 quinazoline, *J* = 8.4 Hz), 11.81 (s, 1H, NH); ^13^C NMR (DMSO-*d*_6_): 56.26, 113.06, 115.15, 120.53, 123.43, 124.56, 125.20, 128.44, 128.54, 130.45, 131.60, 132.08, 136.61, 141.48, 157.04, 159.39, 164.22, 167.29, 167.72; Anal. Calcd. For: C_22_H_17_N_3_O_3_ (371.40): C, 71.15; H, 4.61; N, 11.31; Found: C, 71.02; H, 4.59; N, 11.26.

### Biological evaluation

#### CA inhibitory assay

All the newly synthesised quinazoline-based carboxylic acid derivatives (**6a–c**, **7a–c** and **8a–c**) were assessed for their CA catalysed CO_2_ hydration activities against hCA isoforms I, II, IX and XII by the stopped flow CO_2_ hydrase assay as reported previously^37–40^ (Supporting Materials).

#### *In vitro* NCI-59 cancer cell lines assays

The NCI-USA anticancer assays was performed utilising the NCI, Bethesda, Drug Evaluation Branch protocol,^41–43^ using the SRB cytotoxicity assay,^44^ as desribed earlier.^45^^,^^46^

## Results and discussion

### Chemistry

**Scheme 1.**
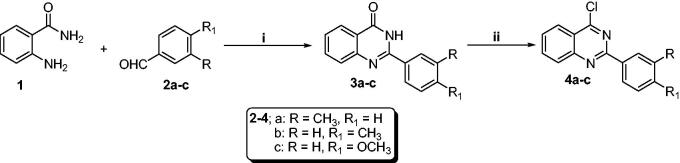
Synthesis of chloroquinazolines (**4a–c**): Reaction conditions (i) FeCl_3_/H_2_O/heating 80 °C/3h, (ii) POCl_3_/*N,N*-dimethylformamide (cat.)/heating 90 °C/4h.

**Scheme 2.**
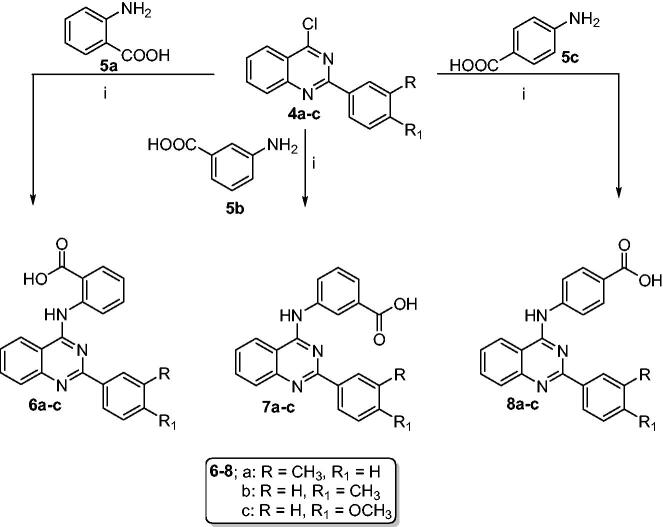
Synthesis of 2-aryl-quinazolin-4-yl aminobenzoic acids (**6a–c**, **7a–c** and **8a–c**): Reaction conditions (i) Isopropanol/HCl (cat.)/reflux/2h.

The synthetic strategy to develop the target 2-aryl-quinazolin-4-yl aminobenzoic acid derivatives (**6a–c**, **7a–c**, and **8a–c**) were represented in Schemes 1 and 2. Synthesis of intermediates (**3a–c**) was carried out by reacting different aldehydes (**2a–c**) with anthranilamide (**1**) in an aqueous solution of FeCl_3_. The key intermediates (**4a–c**) were then synthesised *via* a chlorination reaction of quinazolinone derivatives (**3a–c**) with phosphrous oxychloride in the presence of the catalytic amount of *N,N-*dimethylformamide (Scheme 1).

The target 2-aryl-quinazolin-4-yl aminobenzoic acids (**6a–c**, **7a–c,** and **8a–c**) were obtained, with a yield of 65–86%, by reacting 2-aryl-4-chloroquinazoline derivatives (**4a–c**) with aminobenzoic acid derivatives (**5a–c**) in refluxing isopropanol containing few drops of HCl (Scheme 2).

The target quinazoline derivatives (**6a–c**, **7a–c** and **8a–c**) were structurally confirmed by spectral and elemental analyses. The ^1^H NMR spectra of all compounds revealed a singlet signal around *δ* 11.70–12.34 *ppm* due to the proton of the NH group. Moreover, all compounds showed two doublet signals in the aromatic region around *δ* 7.49–8.40 and 8.61–9.03 *ppm* that are attributable to H5 and H8 of quinazoline moiety, respectively. In addition, ^1^H NMR spectra for derivatives (**6a–b**, **7a–b** and **8a–b**) showed another singlet signal for the CH_3_ group at the range of *δ* 2.41–2.43 *ppm*, whereas, the ^1^H NMR spectra for (**6c**, **7c** and **8c**) disclosed the singlet signal of the OCH_3_ group around *δ* 3.87–3.90 *ppm*. One the other hand, ^13^C NMR spectra for the target quinazoline derivatives confirmed the presence of the carboxylic C=O functionality at *δ* 162–170 *ppm*. Furthermore, ^13^C NMR spectra for compounds (**6a–b**, **7a–b**, and **8a–b**) showed a signal at *δ* 21.41–21.70 *ppm* for the CH_3_ carbon, whereas spectra of compounds (**6c**, **7c**, and **8c**) displayed a signal at *δ* 56.02–56.25 *ppm* for the OCH_3_ carbon.

### Biological evaluation

#### Carbonic anhydrase inhibition

All the newly synthesised quinazoline-based carboxylic acid derivatives (**6a–c**, **7a–c,** and **8a–c**) were assessed for their inhibitory activities against the hCA I, II (cytosolic), IX and XII (trans membrane, tumour associated) isoforms by the stopped-flow CO_2_ hydrase assay.^37^ Acetazolamide (AAZ) was used as a standard CA inhibitor. The data is summarised in Table 1.

**Table 1.** Inhibition data of hCA isoforms I, II, IX, XII, for carboxylic acids (**6a–c**, **7a–c**, and **8a–c**) by a stopped flow CO_2_ hydrase assay.

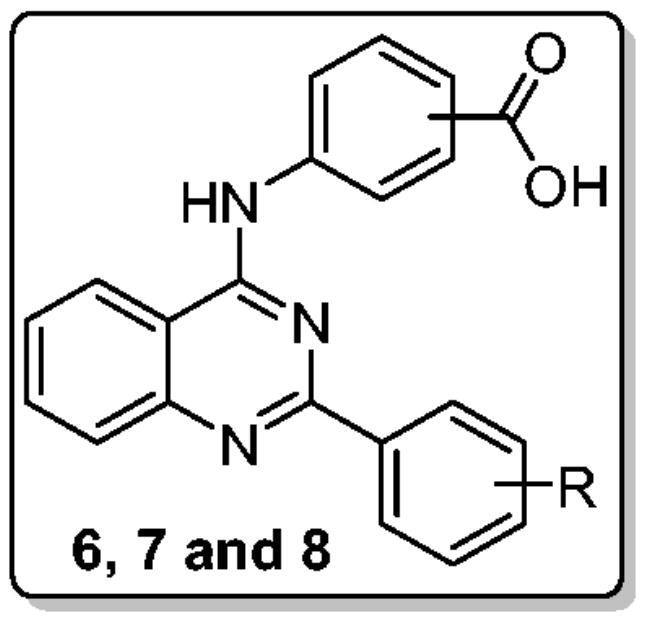						
			*K*_I_ (µM)^a^			
Cmpd	COOH	*R*	hCA I	hCA II	hCA IX	hCA XII
**6a**	2-COOH	3-CH_3_	>100	85.2	42.5	9.0
**6b**	2-COOH	4-CH_3_	>100	48.2	34.4	7.1
**6c**	2-COOH	4-OCH_3_	>100	83.1	46.4	8.9
**7a**	3-COOH	3-CH_3_	>100	26.0	29.3	4.8
**7b**	3-COOH	4-CH_3_	>100	41.7	24.2	0.91
**7c**	3-COOH	4-OCH_3_	>100	85.8	31.6	0.48
**8a**	4-COOH	3-CH_3_	87.7	9.3	4.3	3.8
**8b**	4-COOH	4-CH_3_	73.2	3.9	1.6	0.42
**8c**	4-COOH	4-OCH_3_	66.3	4.6	4.5	0.25
**AAZ**	–	–	0.25	0.01	0.02	0.006

^a^Mean from 3 different assays (errors were in the range of ± 5–10% of the reported values).

Only three of the tested quinazoline-based carboxylic acids (**8a**, **8b,** and **8c**) weakly inhibited the cytosolic hCA I isoform, with inhibition constants (*K*_I_s) equal 87.7, 73.2, and 66.3 µM, respectively, whereas quinazoline derivatives **6a–c** and **7a–c** could not inhibit hCA I up to 100 µM. These results revealed that grafting the carboxylic acids functionality at the *para* position (**8a–c**) could result in modest hCA I inhibitory activity, while shifting to ortho- (**6a–c**) or meta- (**7a–c**) positions resulted in the elimination of hCA I inhibitory activity (*K*_I_s > 100 M), Table 1.

The cytosolic hCA II was effectively inhibited by *para*-aminobenzoic acid-bearing quinazolines (**8a–c**) with *K*_I_s of 9.3, 3.9 and 4.6 µM, respectively, whereas, their *ortho* (**6a–c**) and *meta* (**7a–c**) regioisomers elicited modest inhibitory effects with inhibition constants spanning in the range of 26.0 − 85.8 µM. It is worth to mention that substitution of the 2-phenyl motif with a 4-methyl group, in series **8**, led to compound **8b** with the best hCA II inhibitory activity (*K*_I_ = 3.9 µM).

Similar to the hCA I and hCA II inhibition profiles, the obtained *K*_I_ values disclosed that the cancer-related hCA IX isoform was inhibited most effectively by *para*-aminobenzoic acid-bearing quinazolines (**8a–c**) with *K*_I_s equal 4.3, 1.6 and 4.5 µM, respectively. In addition, hCA IX was moderately affected by quinazolines decorated with *ortho* and *meta* aminobenzoic acid motifs with *K*_I_s ranging between 24.2 and 46.4 µM. The order of activities of target quinazoline-based carboxylic acids towards hCA IX was increased in the order of *para* isomers **8** (*K*_I_s: 1.6 − 4.5 µM) > *meta* isomers **7** (*K*_I_s: 24.2 − 31.6 µM) > *ortho* isomers **6** (*K*_I_s: 34.4 − 46.4 µM), Table 1. Regarding the impact of substitution on the 2-phenyl moiety, within series **6**, **7**, and **8**, it was found that the order of hCA IX inhibitory activities was 4-methyl derivatives (**6b**, **7b**, and **8b**; *K*_I_s = 34.4, 24.2 and 1.6 µM) > 3-methyl derivatives (**6a**, **7a**, and **8a**; *K*_I_s = 42.5, 29.3 and 4.3 µM) > 4-methoxy derivatives (**6c**, **7c** and **8c**; *K*_I_s = 46.4, 31.6 and 4.5 µM), Table 1.

**Figure 2.**
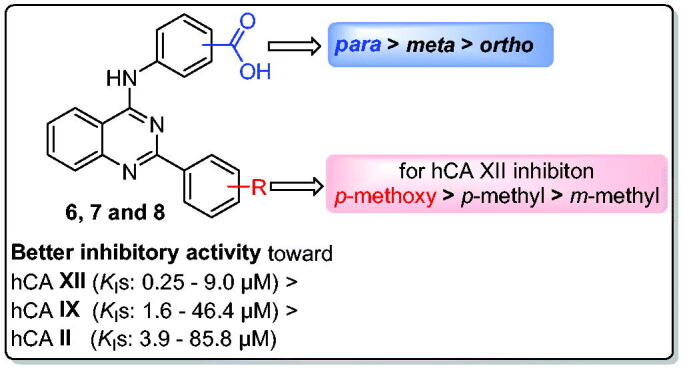
SARs summary for the hCA inhibitory activities of target quinazolines.

The second cancer-related isoform studied in this study is hCA XII, which is also the most vulnerable to the prepared molecules. All quinazoline-based carboxylic acids (**6a–c**, **7a–c**, and **8a–c**) exhibited good inhibition of hCA XII (*K*_I_s: 0.25 − 9.0 µM), as seen by the data in Table 1. In particular, the best hCA XII inhibitory effect was exerted by quinazoline **8c** with a *K*_I_ value equals 0.25 µM. Besides, quinazolines **7b**, **7c**, and **8b** displayed also sub-micromolar inhibitory activity towards hCA XII with *K*_I_ values 0.91, 0.48, and 0.42 µM, respectively. Similarly to the abovementioned deduced Structure-Activity Relationship (SAR) for hCA I, II, and IX isoforms, the order of hCA XII inhibitory activities was increased in the order of *para* isomers **8** (*K*_I_s: 0.25 − 3.8 µM) > *meta* isomers **7** (*K*_I_s: 0.48 − 4.8 µM) > *ortho* isomers **6** (*K*_I_s: 7.1–9 µM), Table 1. Also, it’s noteworthy that appending *p*-methoxyphenyl moiety at C-2 of quianzoline within series **7** and **8** (**7c** and **8c**; *K*_I_s = 0.48 and 0.25 µM) resulted in a better hCA XII inhibitory activity than *p*-methylphenyl (**7b** and **8b**; *K*_I_s = 0.91 and 0.42 µM) and *m*-methylphenyl (**7a** and **8a**; *K*_I_s = 4.8 and 3.8 µM) moieties. The SAR for the inhibitory activity of the new quinazolines towards different hCA isoforms is summarised in Figure 2.

As a result of the inhibitory profile for the reported quinazoline-based carboxylic acid derivatives (**6a–c**, **7a–c**, and **8a–c**) (Table 1), the selectivity index (SI) for each derivative was calculated and presented in Table 2. Regarding the selectivity towards CA IX and XII isoforms, most the examined quinazoline-based carboxylic acids (**6a–c**, **7a–c**, and **8a–c**) exhibited low to moderate selectivity, except compounds **7b** and **7c** that disclosed excellent selectivity towards hCA XII over hCA I (SI = 109.9 and 208.3, respectively), and hCA II (SI = 45.82 and 178.75, respectively), in addition, compounds **8b** and **8c** displayed outstanding selectivity towards hCA XII over hCA I (SI = 174.3 and 265.2, respectively), Table 2.

**Table 2.** Selectivity ratios for the inhibition of CA IX and XII isoforms over CA I and II isoforms for carboxylic acids (**6a–c**, **7a–c**, and **8a–c**) and acetazolamide.

Compound	I/IX	II/IX	I/XII	II/XII
**6a**	2.4	2.00	11.1	9.47
**6b**	2.9	1.40	14.1	6.79
**6c**	2.2	1.79	11.2	9.34
**7a**	3.4	0.89	20.8	5.42
**7b**	4.1	1.72	109.9	45.82
**7c**	3.2	2.72	208.3	178.75
**8a**	20.4	2.16	23.1	2.45
**8b**	45.8	2.44	174.3	9.29
**8c**	14.7	1.02	265.2	18.40
**AAZ**	10.0	0.5	43.9	2.2

#### *In vitro* anti-proliferative activity

The structures of all novel quinazoline based-carboxylic acids prepared in this study were submitted to the National Cancer Institute (NCI-USA), and six compounds (**6a–c**, **7b**, and **8a–b**) were chosen for *in vitro* anti-proliferative activity evaluation against fifty-nine human cancer cell lines representing nine tumour subpanels, according to Bethesda, Drug Evaluation Branch Protocol.^41–43^

##### Preliminary single dose screening at 10 µM concentration

The anti-proliferative activities of the selected quinazoline derivatives (**6a–c**, **7b**, and **8a–b**) have been evaluated at single (10 μM) dose assay using the SRB protocol.^44^ The obtained data was presented as a mean-graph of the percentage growth of the various treated cancer cells and was displayed in Table 2 as the percentage growth inhibition (GI%) induced by the investigated compounds.

Examining the data in Table 3 revealed that the tested quinazoline-based-carboxylic acids (**6a–c**, **7b**, and **8a–b**) demonstrated diverse patterns of sensitivity and selectivity against the various NCI cancer cell panels. Quinazoline derivatives featuring *ortho* aminobenzoic acid (**6a–c**) showed excellent broad cell growth inhibitory activity (GI % mean = 63, 84, and 52, respectively) against most of all cancer cell lines, whereas compounds (**7b** and **8a–b**) with *meta* and *para*-aminobenzoic acid moiety showed fair selective activity (GI % mean = 14, 16 and 20, respectively) towards certain cancer cell lines as shown in Table 3.

**Table 3.** Percentage growth inhibition (GI%) of subpanel tumour cell lines at 10 μM concentration of the quinazoline based-carboxylic acids (**6a–c**, **7b**, and **8a–b**).

Subpanel/cell line		Compound^a^					
		6a	6b	6c	7b	8a	8b
Leukaemia	CCRF-CEM	82	87	58	21	25	28
	HL-60(TB)	60	82	67	–	–	24
	K-562	71	82	69	20	–	23
	MOLT-4	65	89	64	–	22	24
	RPMI-8226	71	83	80	29	30	45
	SR	77	81	NT	41	32	38
Non-Small Cell Lung Cancer	A549/ATCC	56	70	60	–	22	–
	EKVX	79	87	66	41	–	28
	HOP-62	54	72	49	–	–	–
	HOP-92	105	120	78	29	29	25
	NCI-H226	64	85	47	25	52	62
	NCI-H23	79	104	38	–	52	–
	NCI-H322M	52	66	24	–	–	–
	NCI-H460	81	109	89	–	22	–
	NCI-H522	40	67	63	–	–	–
Colon cancer	COLO 205	–	–	–	–	–	–
	HCC-2998	73	95	38	–	24	28
	HCT-116	78	93	62	26	–	22
	HCT-15	86	95	61	55	29	49
	HT29	60	82	37	–	–	–
	KM12	70	92	64	32	23	42
	SW-620	65	86	61	–	–	–
CNS cancer	SF-268	42	89	43	31	37	30
	SF-295	73	94	60	34	36	42
	SF-539	52	79	37	27	–	21
	SNB-19	79	91	55	28	33	29
	SNB-75	45	84	68	–	–	–
	U251	73	82	66	21	27	–
Melanoma	LOX IMVI	64	91	50	–	29	43
	MALME-3M	63	80	31	–	–	–
	M14	75	87	48	–	–	–
	MDA-MB-435	55	77	44	–	–	–
	SK-MEL-2	34	94	57	–	–	–
	SK-MEL-28	50	71	35	–	–	–
	SK-MEL-5	94	124	70	–	29	28
	UACC-257	51	71	28	–	–	–
	UACC-62	72	89	44	–	–	–
Ovarian cancer	IGROV1	41	69	21	–	–	–
	OVCAR-3	66	94	54	–	–	20
	OVCAR-4	58	78	66	23	–	26
	OVCAR-5	23	34	27	–	–	–
	OVCAR-8	40	69	40	–	–	–
	NCI/ADR-RES	75	95	46	–	–	24
	SK-OV-3	24	69	53	–	–	–
Renal cancer	786-0	75	81	48	24	29	–
	A498	52	78	41	–	54	–
	ACHN	63	92	54	24	–	24
	CAKI-1	48	79	53	–	–	–
	SN12C	59	78	32	23	42	51
	TK-10	–	41	52	–	–	–
	UO-31	69	87	25	51	54	58
Prostate	PC-3	71	84	60	–	–	23
	DU-145	50	81	43	–	–	–
Breast cancer	MCF7	76	92	49	–	–	37
	MDA-MB-231	34	65	–	–	–	–
	HS 578 T	84	109	72	34	48	32
	BT-549	100	123	62	–	34	41
	T-47D	73	89	71	–	22	32
	MDA-MB-468	115	126	57	–	–	–
Mean GI %		63	84	52	14	16	20
Number of sensitive cell lines		56	58	56	21	25	30

^a^Only GI% higher than 20% are shown.

In particular, quinazoline derivative **6b** stood out as the most effective anti-proliferative compound (GI % mean = 84). Compound **6b** exhibited excellent activity with GI% more than 75% against the examined cancer cell lines from all subpanels, except non-small cell lung (A549, HOP-62, NCI-H322M and NCI-H522), colon (COLO 205), melanoma (SK-MEL-28 and UACC-257), ovarian (IGROV1, OVCAR-5, OVCAR-8 and SK-OV-3), renal (TK-10) and breast (MDA-MB-231) cancer cell lines. In addition, compound **6b** showed good activity towards non-small cell lung (A549, HOP-62, NCI-H322M and NCI-H522), melanoma (SK-MEL-28 and UACC-257), ovarian (IGROV1, OVCAR-8, and SK-OV-3), breast (MDA-MB-231) cancer cell lines with GI% of 70, 72, 66, 67, 71, 71, 69, 69, 69 and 65% respectively. It is noteworthy to mention that quinazoline derivative **6b** had a lethal cytotoxic effect against non-small cell lung (HOP-92, NCI-H23, and NCI-H460), melanoma (SK-MEL-5), and breast (HS 578 T, BT-549, and MDA-MB-468) cancer cell lines with GI% equal 120, 104, 109,124, 109, 123 and 126% respectively.

Moreover, quinazoline **6a** disclosed a broad**-**spectrum anticancer effect against 56 cell lines representing all subpanels and emerged as the second most active compound in this assay (mean % GI = 63). Superiorly, quinazoline **6a** exerted effective cell growth inhibitory activity with GI% more than 75% against leukaemia (CCRF-CEM and SR), non-small cell lung (EKVX, NCI-H23 and NCI-H460), colon (HCT-116 and HCT-15), CNS (SNB-19), melanoma (MDA-MB-435 and SK-MEL-5), ovarian (NCI/ADR-RES), renal (786–0) and breast (MCF7, HS 578 T and BT-549) cancer cell lines. In addition, compound 6a possessed a lethal impact towards non-small cell lung (HOP-92) and breast (MDA-MB-468) cancer cell lines with GI% of 105 and 115% respectively.

##### *In vitro* NCI 5-dose assay

The preliminary screening data showed that quinazoline-carboxylic acid **6b** (NSC: 835857) was the most active anticancer molecule in this study, with promising activity against numerous tumour cell lines. Thus, **6b** was selected by NCI for further evaluations at **a** 5-doses (0.01–100 µM) level. Three dose**-**response parameters (GI_50_, TGI, and LC_50_) were calculated and displayed in Table 4.

**Table 4.** GI_50_, TGI, and LC_50_ values of NCI five doses anticancer assay for **6b** (NSC: 835857).

Cancer type/cells	Compound **6b** (NSC: 835857)		
	GI_50_ (µM)	TGI (µM)	LC_50_ (µM)
Leukaemia			
MOLT-4	11.7	<100	<100
RPMI-8226	4.4	<100	<100
K-562	7.05	<100	<100
SR	10.3	<100	<100
CCRF-CEM	5.7	<100	<100
HL-60(TB)	12.9	75.4	<100
Non-small cell lung cancer			
HOP-92	2.9	29.3	<100
NCI-H226	10.2	54.2	<100
NCI-H522	4.4	27.1	<100
NCI-H322M	13.6	<100	<100
NCI-H460	8.5	27.7	84.0
NCI-H23	15.7	44.4	<100
EKVX	8.9	56.2	<100
HOP-62	10.8	33.7	<100
A549/ATCC	8.31	<100	<100
Colon cancer			
KM 12	13.7	81.2	<100
SW-620	18.2	<100	<100
HT29	17.9	<100	<100
HCT-15	5.8	28.7	<100
COLO 205	17.8	41.0	94.1
HCC-2998	12.4	36.0	<100
HCT-116	10.1	36.1	<100
CNS cancer			
SNB-75	1.4	80.3	<100
U251	5.6	35.0	<100
SF-539	11.9	28.3	67.2
SNB-19	8.1	81.7	<100
SF-295	7.04	27.0	86.0
SF-268	14.3	<100	<100
Melanoma			
MDA-MB-435	13.9	87.2	<100
UACC-62	10.2	27.0	71.6
M14	15.5	77.4	<100
UACC-257	15.3	90.3	<100
SK-MEL-5	5.3	20.3	57.5
SK-MEL-28	15.9	97.4	<100
LOX IMVI	10.2	31.6	97.7
MALME-3M	15.6	64.5	<100
SK-MEL-2	3.7	15.6	83.5
Ovarian cancer			
IGROV1	19.1	87.1	<100
OVCAR-4	8.8	<100	<100
OVCAR-5	21.8	68.9	<100
OVCAR-8	18.2	<100	<100
NCI/ADR-RES	16.8	98.5	<100
SK-OV-3	11.9	55.3	<100
Renal cancer			
786-0	17.1	<100	<100
A498	19.9	58.1	<100
ACHN	6.4	91.1	<100
CAKI-1	13.8	<100	<100
RXF 393	13.1	65.9	<100
SN 12 C	11.0	60.4	<100
TK-10	29.3	<100	<100
UO-31	7.3	77.9	<100
Prostate cancer			
PC-3	12.7	<100	<100
DU-145	17.7	<100	<100
Breast cancer			
MCF7	9.4	89.9	<100
MDA-MB-231	17.2	59.7	<100
HS 578 T	10.2	67.0	<100
BT-549	14.6	33.8	78.1
T-47D	6.3	70.0	<100
MDA-MB-468	8.3	42.0	<100

Results displayed in Table 4, disclosed that compound **6b** exhibited good anti-proliferative activities towards all the examined human cancer cell subpanels with GI_50_ values range 1.4 − 19.9 μM, except for renal TK-10 cell line (GI_50_ = 29.3 μM). In particular, the best anti-proliferative activity was noticed for non-small cell (HOP-92), CNS (SNB-75), and melanoma (SK-MEL-2) cancer cell lines with GI_50_ values equal 2.9, 1.4, and 3.7 μM, respectively (Table 4).

Concerning the cytostatic impact of quinazoline **6b**, it showed moderate to good effect towards melanoma (SK-MEL-2), non-small cell (HOP-92, NCI-H522, NCI-H460, and HOP-62), colon (HCT-15, COLO 205, HCC-2998, and HCT-116), CNS (U251, SF-539, and SF-295), melanoma (UACC-62, SK-MEL-5, and LOX IMVI), and breast (BT-549, and MDA-MB-468) with TGI range = 15.6– 42 μM. It is worthy to mention that **6b** exhibited LC_50_ values more than 100 μM and considered as non-lethal towards all the examined cell lines except for non-small cell (NCI-H460), colon (COLO 205), CNS (SF-539 and SF-295), melanoma (UACC-62, SK-MEL-5, LOX IMVI, and SK-MEL-2), and breast (BT-549) that possessed weak lethal effect with LC_50_ = 84.0, 94.1, 67.2, 86.0, 71.6, 57.5, 97.7, 83.5, and 78.1 μM, respectively (Table 3).

With regard to the sensitivity of the examined cell lines, quinazoline **6b** elicited comparatively homogenous growth inhibitory activity throughout all NCI panels, with good growth inhibition full panel GI_50_ (MG-MID) equals 11.99 μM, as well as subpanel GI_50_ (MG-MID) values spanning from 8.04 to 15.66 μM. In particular, the most susceptible subpanels were CNS and Leukaemia with MG-MID of 8.04 and 8.68 μM, respectively (Table 5). In order to assess the selectivity of **6b**, its full panel MG-MID is divided by its individual subpanel MG-MID (Table 5). The selectivity index for compound **6b** ranged from 0.76 to 1.49 which points out that **6b** has non-selective broad-spectrum anti-proliferative activity towards all NCI cancer subpanels. It is worth to mention that the best anti-proliferative counterpart 6b is not the most active inhibitor against CA IX or XII, thus the target of this compound could be other than CAs.

**Table 5.** Median GI_50_ values (µM) for compound **6b** on subpanel tumour cell lines.

Subpanel tumour cell line	6b	
	MG-MID	Selectivity index
Leukaemia	8.68	1.38
Non-small cell lung cancer	9.24	1.29
Colon cancer	13.70	0.87
CNS cancer	8.04	1.49
Melanoma	11.73	1.02
Ovarian cancer	15.66	0.76
Renal cancer	14.74	0.81
Prostate cancer	15.2	0.78
Breast cancer	10.99	1.09
Full panel MG-MID	11.99	–

## Conclusions

Three sets of 2-aryl-quinazolin-4-yl aminobenzoic acid regioisomers (**6a–c**, **7a–c**, and **8a–c**) were designed and synthesised as new non-classical CA inhibitors. Their CA inhibitory activities towards isoforms I, II, IX, and XII were evaluated. Only three of the tested quinazoline-based carboxylic acids (**8a**, **8b**, and **8c**) weakly inhibited the cytosolic hCA I isoform, with inhibition constants (*K*_I_s) equal 87.7, 73.2, and 66.3 µM. The cytosolic hCA II was effectively inhibited by *para*-aminobenzoic acid-bearing quinazolines (**8a–c**) with *K*_I_s of 9.3, 3.9, and 4.6 µM, respectively, whereas, their *ortho* (**6a–c**) and *meta* (**7a–c**) regioisomers elicited modest inhibitory effects. Moreover, the cancer-related hCA IX isoform was inhibited most effectively by quinazolines (**8a–c**) with *K*_I_s equal 4.3, 1.6, and 4.5 µM, respectively. Also, the results revealed that the cancer-related hCA XII isoform is the most vulnerable to the prepared molecules. In particular, the best hCA XII inhibitory effect was exerted by quinazoline **8c** (*K*_I_ = 0.25 µM), also, quinazolines **7b**, **7c**, and and **8b** displayed sub-micromolar hCA XII inhibitory activity (*K*_I_ = 0.91, 0.48, and 0.42 µM, respectively). The SAR analysis highlighted that the order of hCA inhibitory activities was increased in the order of *para* isomers **8 **>** ***meta* isomers **7 **>** ***ortho* isomers **6**. On the other hand, the anti-proliferative activities of the quinazoline derivatives (**6a–c**, **7b**, and **8a–b**) have been evaluated at single (10 μM) dose assay against 59 cancer cell lines in the NCI-USA. Quinazoline derivatives featuring *ortho* aminobenzoic acid (**6a–c**) showed excellent broad cell growth inhibitory activity (GI % mean = 63, 84 and 52, respectively) against most of all cancer cell lines, whereas compounds (**7b** and **8a–b**) with *meta* and *para* aminobenzoic acid moiety showed fair selective activity (GI % mean = 14, 16, and 20, respectively) towards certain cancer cell lines. Thereafter, **6b** was selected by NCI for further evaluations at 5-doses (0.01–100 µM) level. Quinazoline **6b** elicited comparatively homogenous growth inhibitory activity throughout all NCI panels, with good growth inhibition full panel GI_50_ (MG-MID) equals 11.99 μM, as well as subpanel GI_50_ (MG-MID) values spanning from 8.04 to 15.66 μM. In particular, the most susceptible subpanels were CNS and Leukaemia with MG-MID of 8.04 and 8.68 μM, respectively.

## Supplementary Material

Supplemental Material
