# Supplementary material for: Development of novel anilinoquinazoline-based carboxylic acids as non-classical carbonic anhydrase IX and XII inhibitors
Source: J Enzyme Inhib Med Chem. 2023 Mar 21;38(1):2191163. doi: 10.1080/14756366.2023.2191163 (PMC10035947; doi:10.1080/14756366.2023.2191163)

Supporting Information

Development of Novel Anilinoquinazoline-Based Carboxylic acids as Non-classical Carbonic Anhydrase IX and XII Inhibitors

Zainab M. Elsayed, Hadia Almahli, Alessio Nocentini, Andrea Ammara, Claudiu T. Supuran*, Wagdy M. Eldehna*, Sahar M. Abou-Seri*

* Corresponding authors. E-mail addresses: wagdy2000@gmail.com (W.M. Eldehna), claudiu.supuran@unifi.it (C.T. Supuran), sahar.shaarawy@pharma.cu.edu.eg (S.M. Abou-Seri)

Tables of Contents

1. **Carbonic Anhydrase Inhibition Assay** 2

2. **NCI Anticancer Assay** 3-4

3. **NMR Spectra** 5-30

4. **NCI charts** 31-40

1. Carbonic anhydrase inhibition assay

The carbonic anhydrase catalyzed CO_2 hydration actions for the quinazoline-based carboxylic acid derivatives (**6a-c**, **7a-c** and **8a-c**) were assayed utilizing an instrument of Applied Photophysics stopped-flow, as described previously. The enzymes are recombinant proteins prepared in our lab. Phenol red (at a concentration of 0.2 mM) has been used as indicator, working at the absorbance maximum of 557 nm, with 20 mM Hepes (pH 7.5) as buffer, and 20 mM Na_2SO_4 (for maintaining constant the ionic strength), following the initial rates of the CA-catalyzed CO_2 hydration reaction for a period of 10-100 s. The CO_2 concentrations ranged from 1.7 to 17 mM for the determination of the kinetic parameters and inhibition constants. For each inhibitor at least six traces of the initial 5-10% of the reaction have been used for determining the initial velocity. The uncatalyzed rates were determined in the same manner and subtracted from the total observed rates. Stock solutions of inhibitor (0.1 mM) were prepared in distilled-deionized water and dilutions up to 0.01 nM were done thereafter with the assay buffer. Inhibitor and enzyme solutions were preincubated together for 15 min at room temperature prior to assay, in order to allow for the formation of the E-I complex. The inhibition constants were obtained by non-linear least-squares methods using PRISM 3 and the Cheng-Prusoff equation, and represent the mean from at least three different determinations.

2. NCI Anticancer Assay

The cytotoxicity *in vitro* assay was conducted at National Cancer Institute (NCI), Bethesda, USA against 59 cancer cell lines. The one-dose data were reported as a mean graph of the percent growth of treated cells. The number reported for the one-dose assay was growth relative to the no-drug control and relative to the time zero number of cells. This allowed detection of both growth inhibition (values between 0 and 100) and lethality (values less than 0). For example, a value of 100 means no growth inhibition. A value of 40 would mean 60% growth inhibition. A value of 0 means no net growth over the course of the experiment. A value of -40 would mean 40% lethality. A value of -100 means all cells were dead.

The human tumor cell lines of the cancer screening panel were grown in RPMI 1640 medium containing 5% fetal bovine serum and 2 mM L-glutamine. For a typical screening experiment, cells were inoculated into 96 well microtiter plates in 100 μL at plating densities ranging from 5,000 to 40,000 cells/well depending on the doubling time of individual cell lines. After cell inoculation, the microtiter plates were incubated at 37° C, 5 % CO2, 95 % air and 100 % relative humidity for 24 h prior to addition of experimental drugs. After 24 h, two plates of each cell line were fixed *in situ* with TCA, to represent a measurement of the cell population for each cell line at the time of drug addition (Tz). Experimental drugs were solubilized in dimethyl sulfoxide at 400-fold the desired final maximum test concentration and stored frozen prior to use. At the time of drug addition, an aliquot of frozen concentrate was thawed and diluted to twice the desired final maximum test concentration with complete medium containing 50 μg/mL gentamicin. Additional four, 10-fold or ½ log serial dilutions were made to provide a total of five drug concentrations plus control. Aliquots of 100 μL of these different drug dilutions were added to the appropriate microtiter wells already containing 100 μL of medium, resulting in the required final drug concentrations. Following drug addition, the plates were incubated for an additional 48 h at 37°C, 5 % CO2, 95 % air, and 100 % relative humidity. For adherent cells, the assay was terminated by the addition of cold TCA. Cells were fixed *in situ* by the gentle addition of 50 μL of cold 50 % (w/v) TCA (final concentration, 10 % TCA) and incubated for 60 minutes at 4°C. The supernatant was discarded, and the plates were washed five times with tap water and air dried. Sulforhodamine B (SRB) solution (100 μL) at 0.4 % (w/v) in 1 % acetic acid was added to each well, and plates were incubated for 10 minutes at room temperature. After staining,

unbound dye was removed by washing five times with 1 % acetic acid and the plates were air dried. Bound stain was subsequently solubilized with 10 mM trizma base, and the absorbance was read on an automated plate reader at a wavelength of 515 nm. For suspension cells, the methodology was the same except that the assay was terminated by fixing settled cells at the bottom of the wells by gently adding 50 μL of 80 % TCA (final concentration, 16 % TCA). Using the seven absorbance measurements [time zero, (Tz), control growth, (C), and test growth in the presence of drug at the five concentration levels (Ti)], the percentage growth was calculated at each of the drug concentrations levels. Percentage growth inhibition was calculated as:

[(Ti-Tz)/(C-Tz)] x 100 for concentrations for which Ti>/=Tz

[(Ti-Tz)/Tz] x 100 for concentrations for which Ti<Tz

Three dose response parameters were calculated for each experimental agent. Growth inhibition of 50 % (GI_{50}) was calculated from [(Ti-Tz)/(C-Tz)] x 100 = 50, which is the drug concentration resulting in a 50% reduction in the net protein increase (as measured by SRB staining) in control cells during the drug incubation. The drug concentration resulting in total growth inhibition (TGI) was calculated from Ti = Tz. The LC_{50} (concentration of drug resulting in a 50% reduction in the measured protein at the end of the drug treatment as compared to that at the beginning) indicating a net loss of cells following treatment was calculated from [(Ti-Tz)/Tz] x 100 = -50.

Wagdy Eldahna-Q 9a-DMSO-Hnmr-A

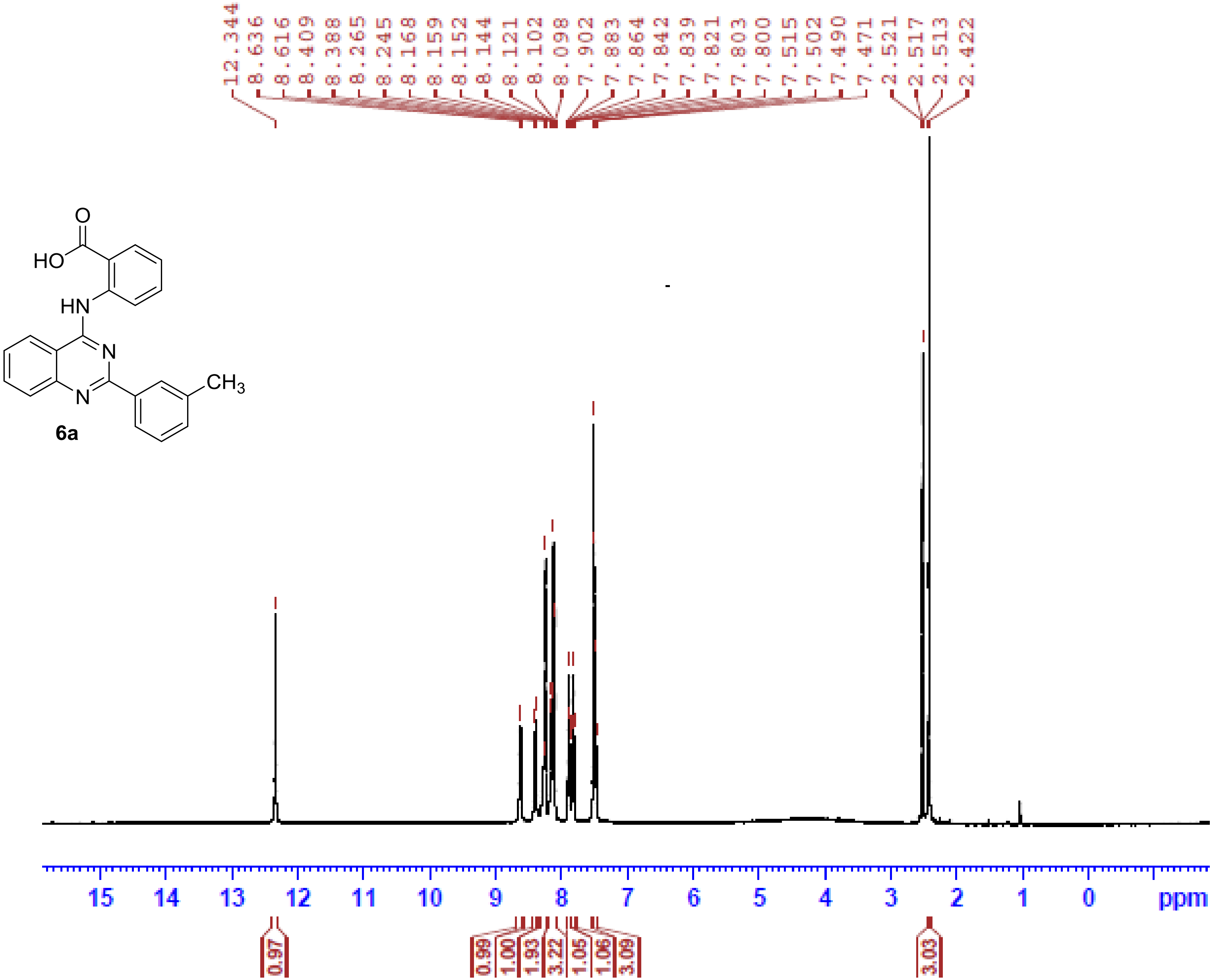

Wagdy Eldahna-Q 9a-DMSO-Hnmr-A

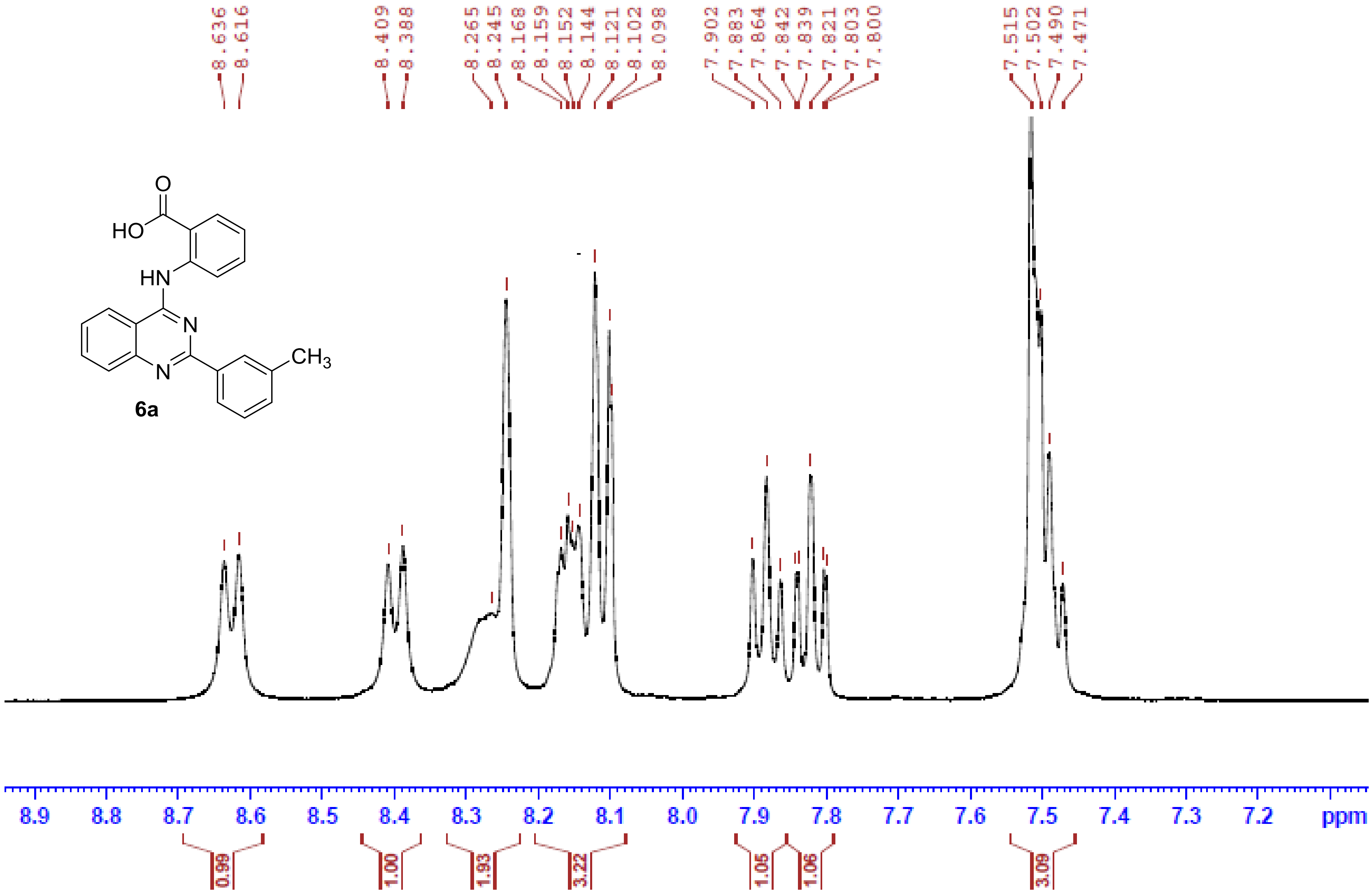

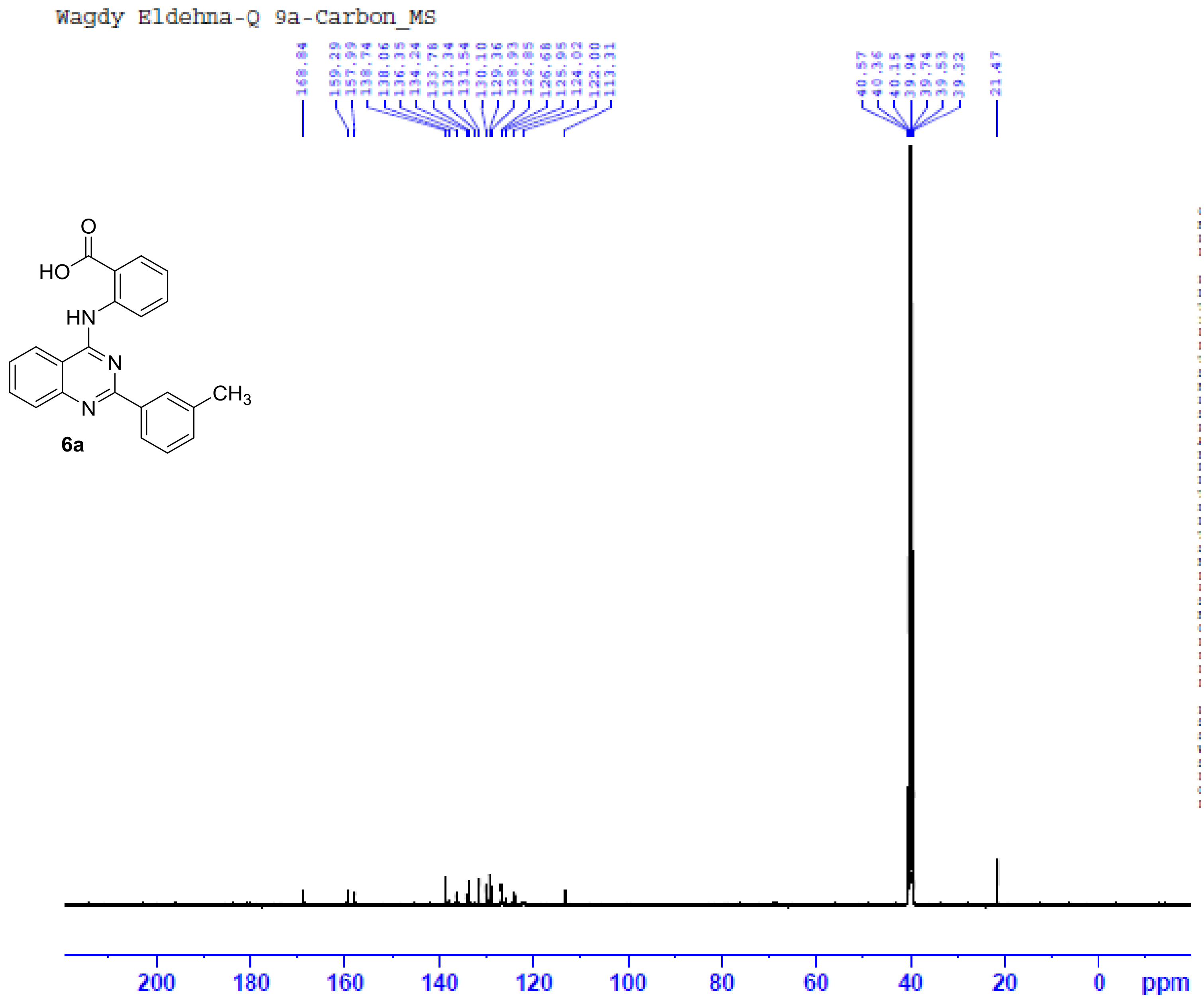

Wagdy Eldehna-Q 9a-Carbon_MS
168.84
159.29
157.99
138.74
138.06
136.35
134.24
133.78
132.34
131.54
130.10
129.36
128.93
126.85
126.68
125.95
124.02
122.00
113.31
40.57
40.36
40.15
39.94
39.74
39.53
39.32
21.47
O
HO
HN
N
N
CH3
6a
200
180
160
140
120
100
80
60
40
20
0
ppm

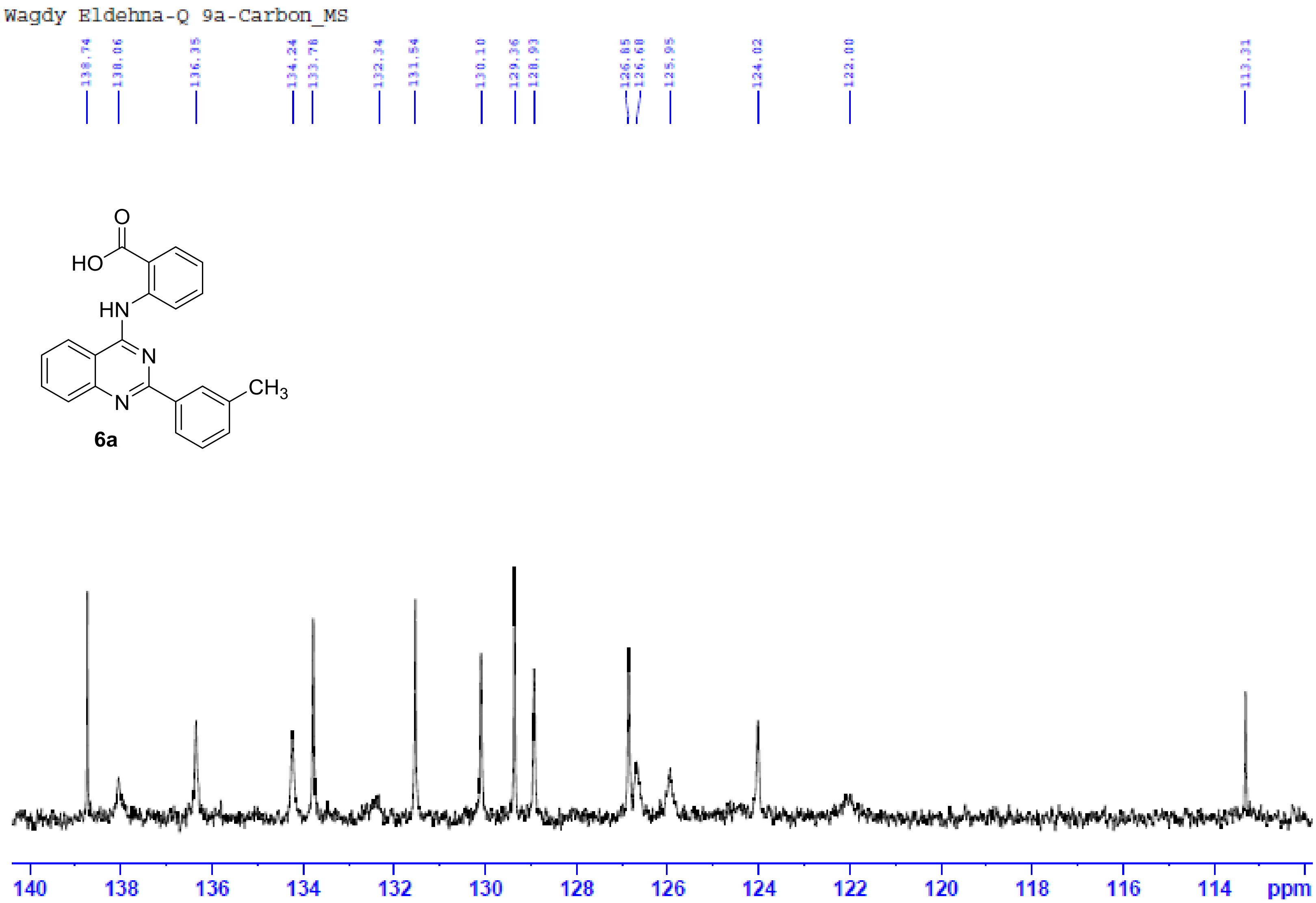
Wagdy Eldehna-Q 9a-Carbon_MS
138.74
138.06
136.35
134.24
133.78
132.34
131.54
130.10
129.36
128.93
126.85
126.68
125.95
124.02
122.00
113.31
O
HO
HN
N
N
CH3
6a
140
138
136
134
132
130
128
126
124
122
120
118
116
114
ppm

Wagdy Eldahna-Q 9d-DMSO-Hnmr-A

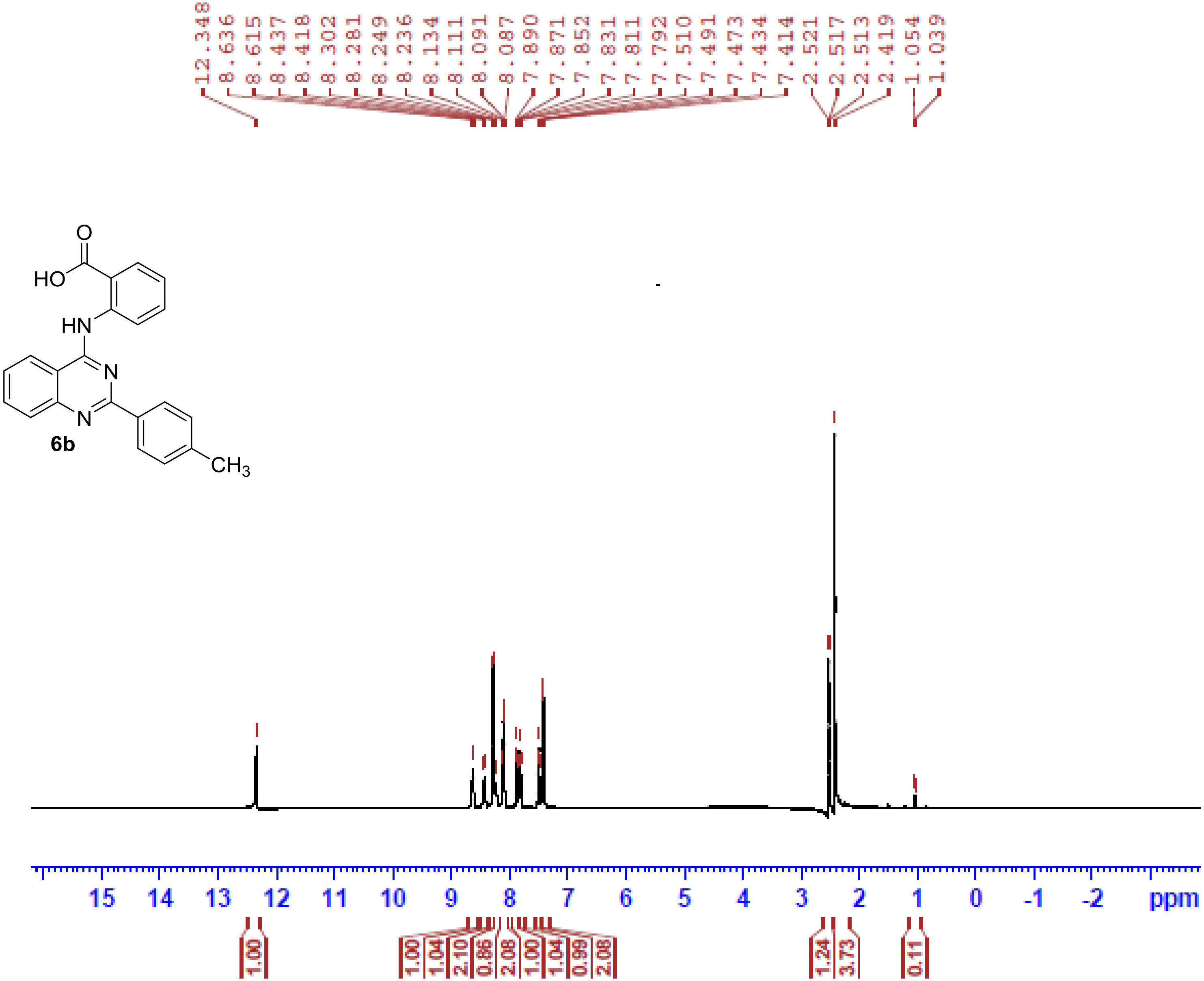

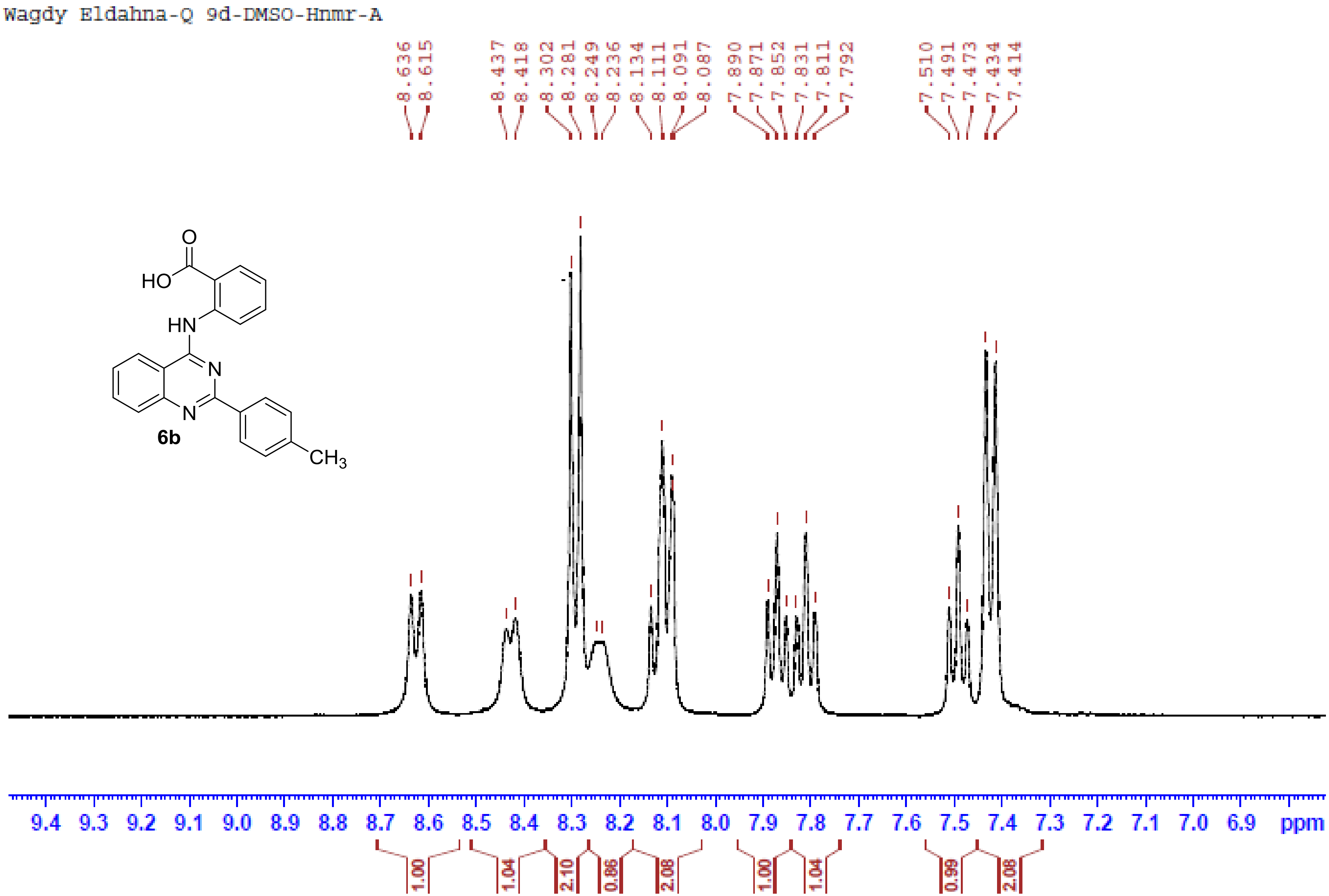
Wagdy Eldahna-Q 9d-DMSO-Hnmr-A
8.636
8.615
8.437
8.418
8.302
8.281
8.249
8.236
8.134
8.111
8.091
8.087
7.890
7.871
7.852
7.831
7.811
7.792
7.510
7.491
7.473
7.434
7.414
O
HO
HN
N
N
6b
CH3
9.4 9.3 9.2 9.1 9.0 8.9 8.8 8.7 8.6 8.5 8.4 8.3 8.2 8.1 8.0 7.9 7.8 7.7 7.6 7.5 7.4 7.3 7.2 7.1 7.0 6.9 ppm
1.00
1.04
2.10
0.86
2.08
1.00
1.04
0.99
2.08

Wagdy Eldehna-Q 9d-Carbon_MS

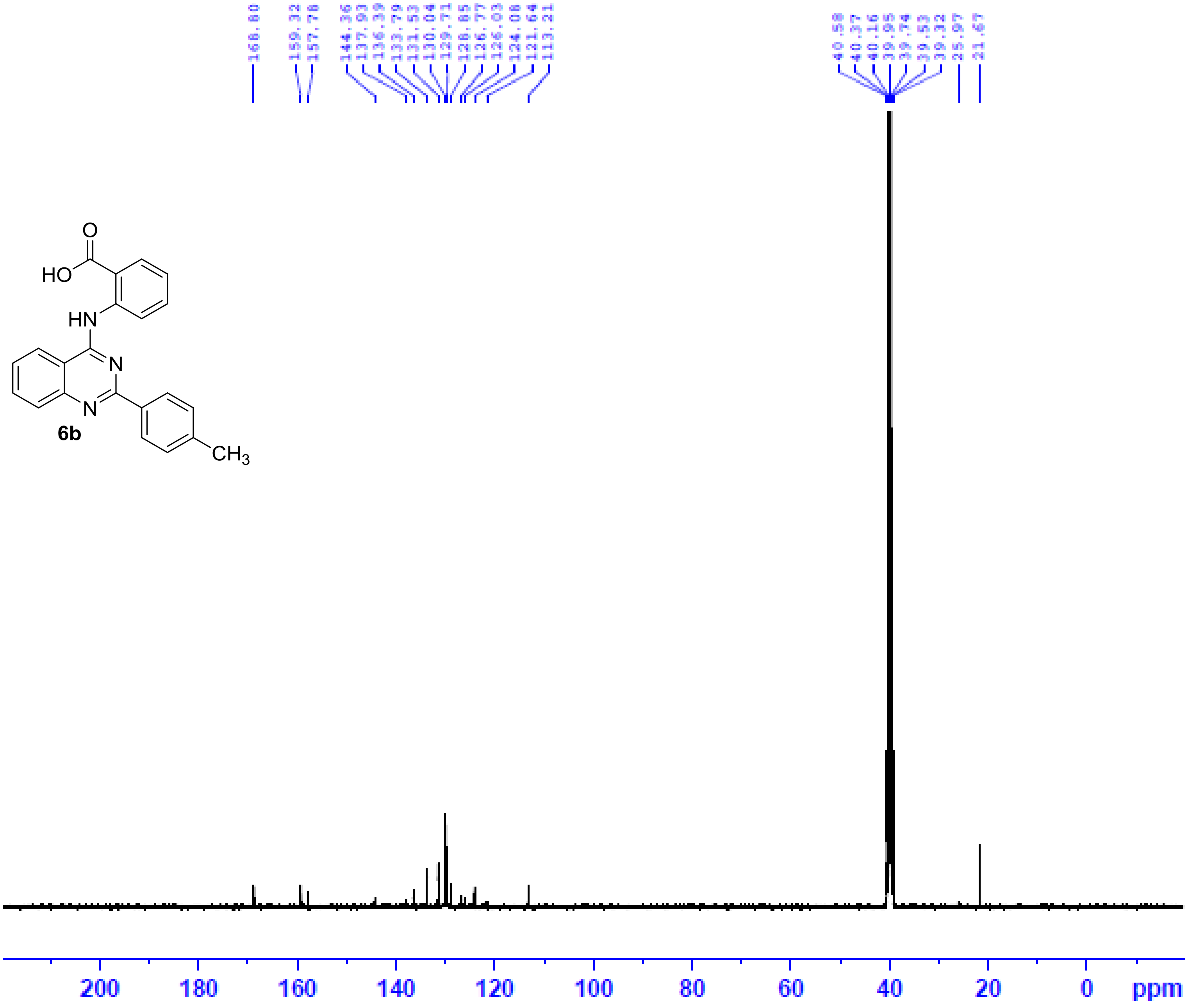

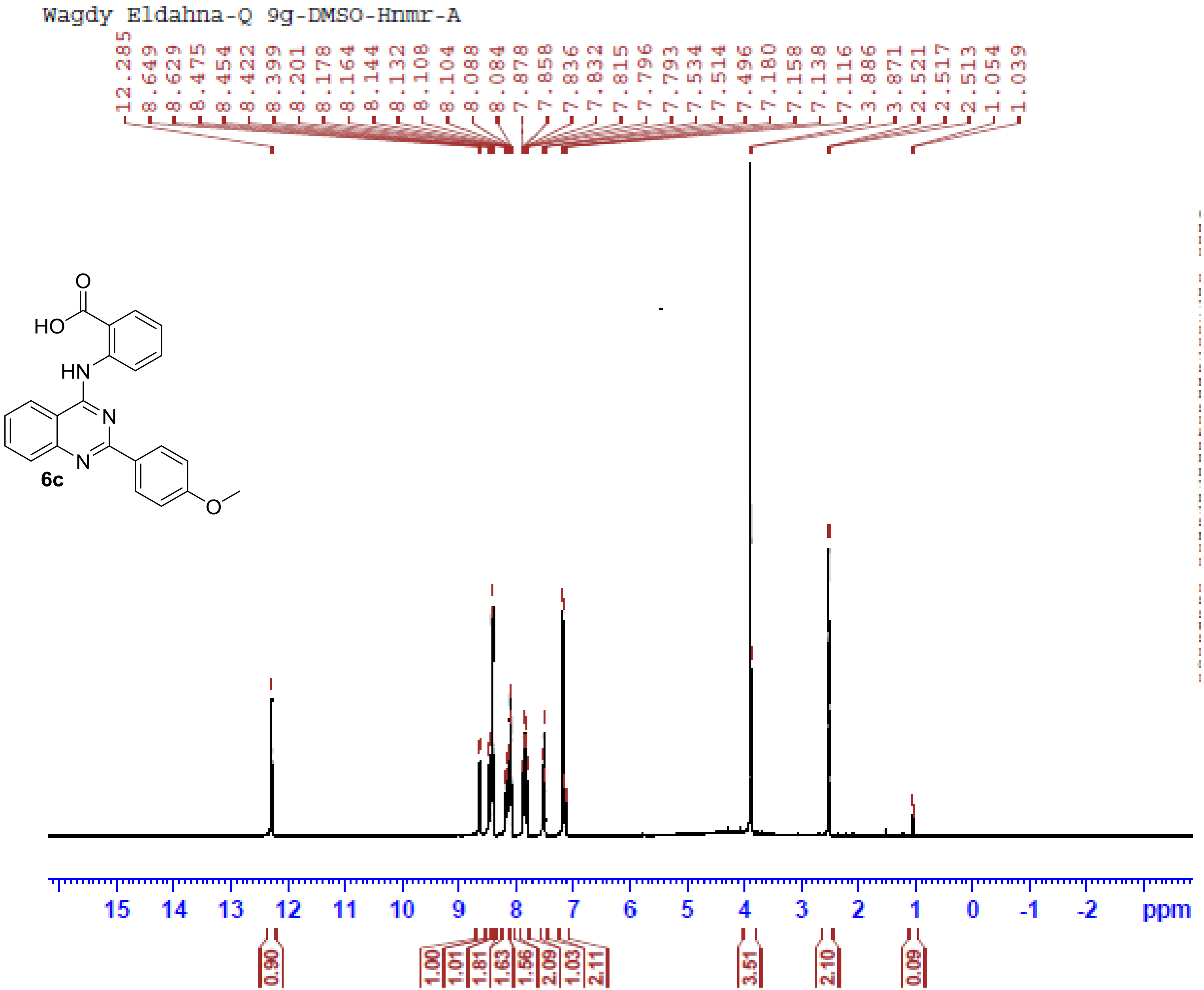

Wagdy Eldahna-Q 9g-DMSO-Hnmr-A
12.285
8.649
8.629
8.475
8.454
8.422
8.399
8.201
8.178
8.164
8.144
8.132
8.108
8.104
8.088
8.084
7.878
7.858
7.836
7.832
7.815
7.796
7.793
7.534
7.514
7.496
7.180
7.158
7.138
7.116
3.886
3.871
2.521
2.517
2.513
1.054
1.039
O
HO
HN
N
N
6c
O
15
14
13
12
11
10
9
8
7
6
5
4
3
2
1
0
-1
-2
ppm
0.90
1.00
1.01
1.81
1.63
1.56
2.09
1.03
2.11
3.51
2.10
0.09

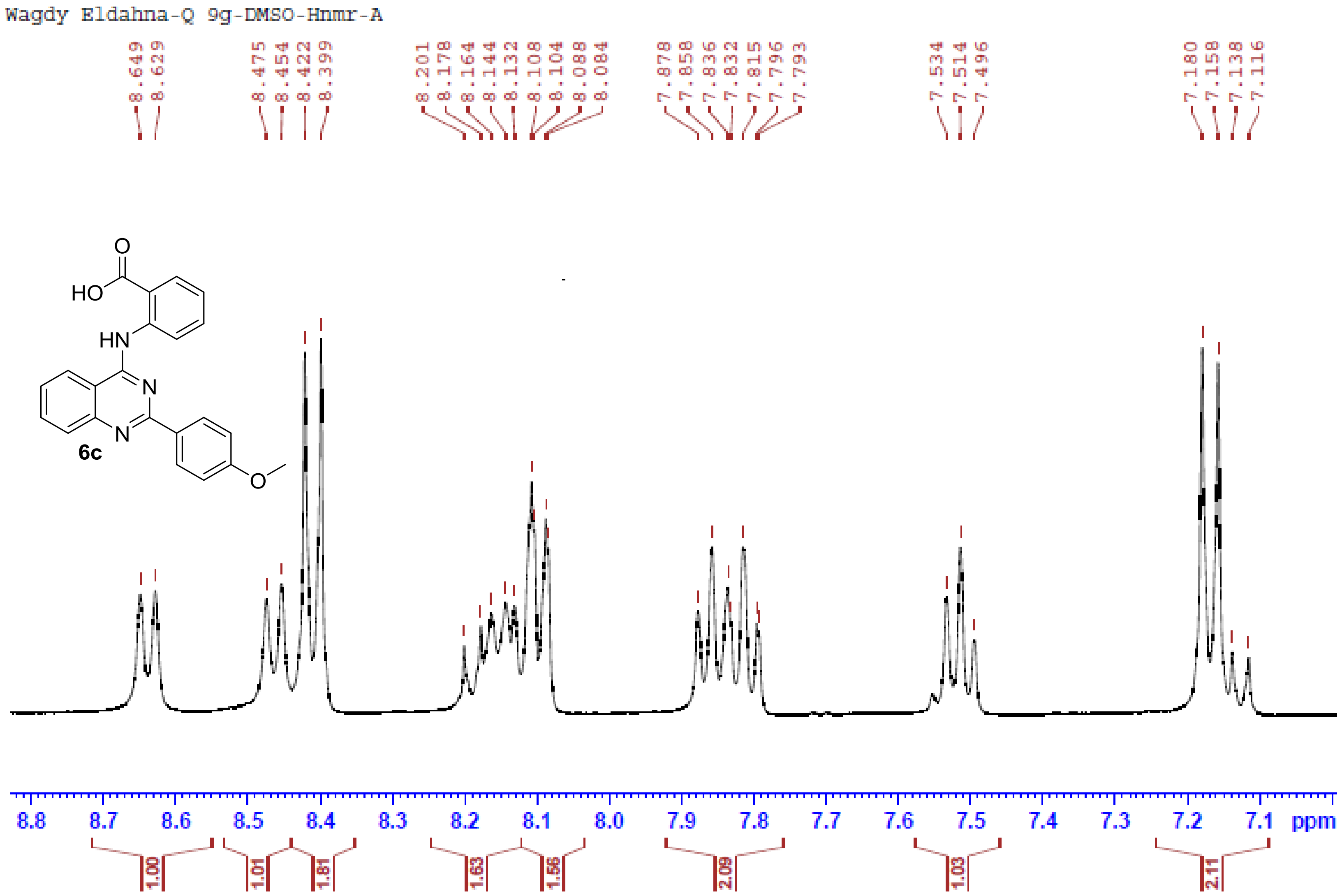

Wagdy Eldahna-Q 9g-DMSO-Hnmr-A
8.649
8.629
8.475
8.454
8.422
8.399
8.201
8.178
8.164
8.144
8.132
8.108
8.104
8.088
8.084
7.878
7.858
7.836
7.832
7.815
7.796
7.793
7.534
7.514
7.496
7.180
7.158
7.138
7.116
O
HO
HN
N
N
O
6c
8.8
8.7
8.6
8.5
8.4
8.3
8.2
8.1
8.0
7.9
7.8
7.7
7.6
7.5
7.4
7.3
7.2
7.1
ppm
1.00
1.01
1.81
1.63
1.56
2.09
1.03
2.11

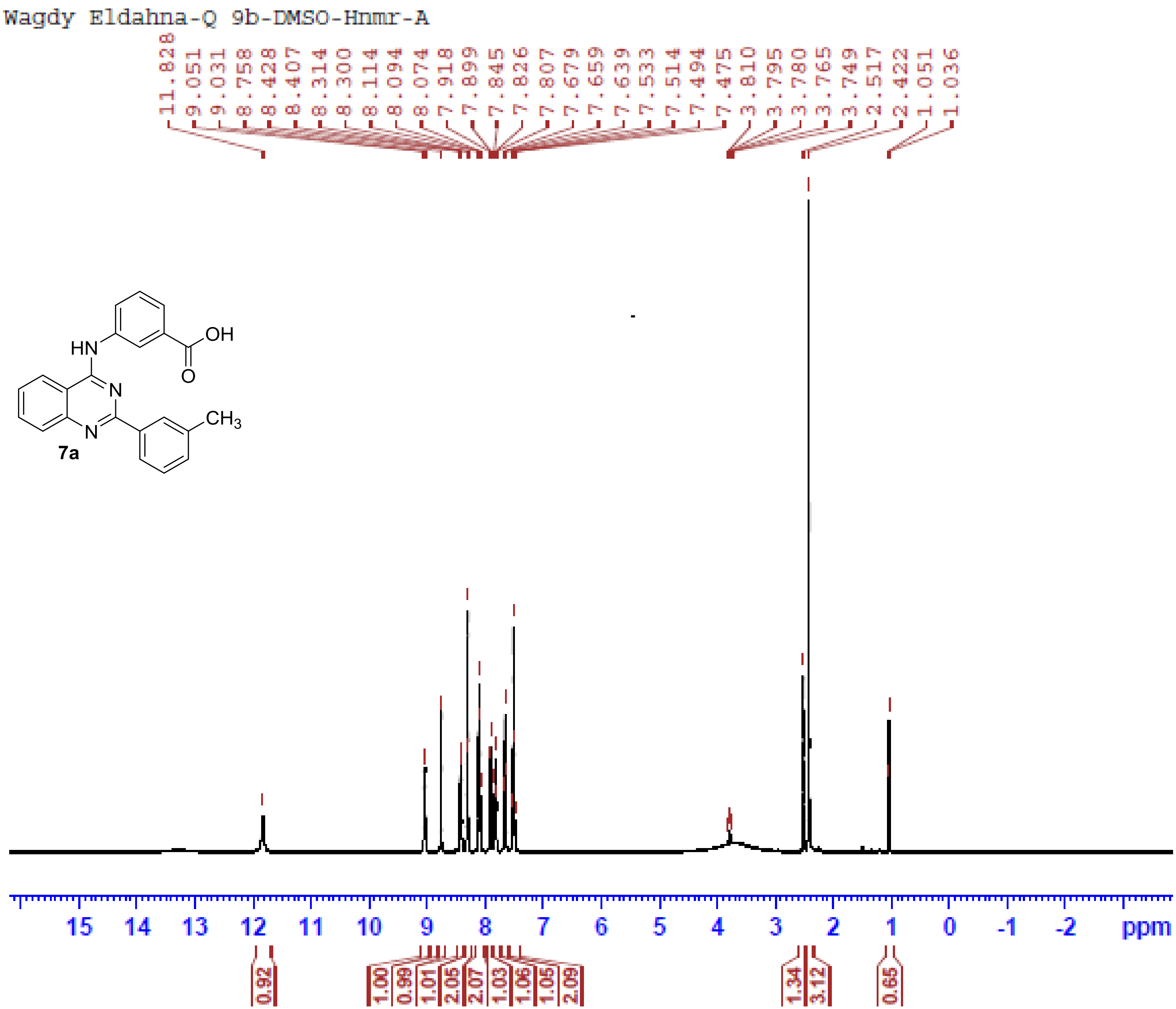
Wagdy Eldahna-Q 9b-DMSO-Hnmr-A
11.828
9.051
9.031
8.758
8.428
8.407
8.314
8.300
8.114
8.094
8.074
7.918
7.899
7.845
7.826
7.807
7.679
7.659
7.639
7.533
7.514
7.494
7.475
3.810
3.795
3.780
3.765
3.749
2.517
2.422
1.051
1.036
HN
OH
O
N
N
CH3
7a
15
14
13
12
11
10
9
8
7
6
5
4
3
2
1
0
-1
-2
ppm
0.92
1.00
0.99
1.01
2.05
2.07
1.03
1.06
1.05
2.09
1.34
3.12
0.65

Wagdy Eldahna-Q 9b-DMSO-Hnmr-A

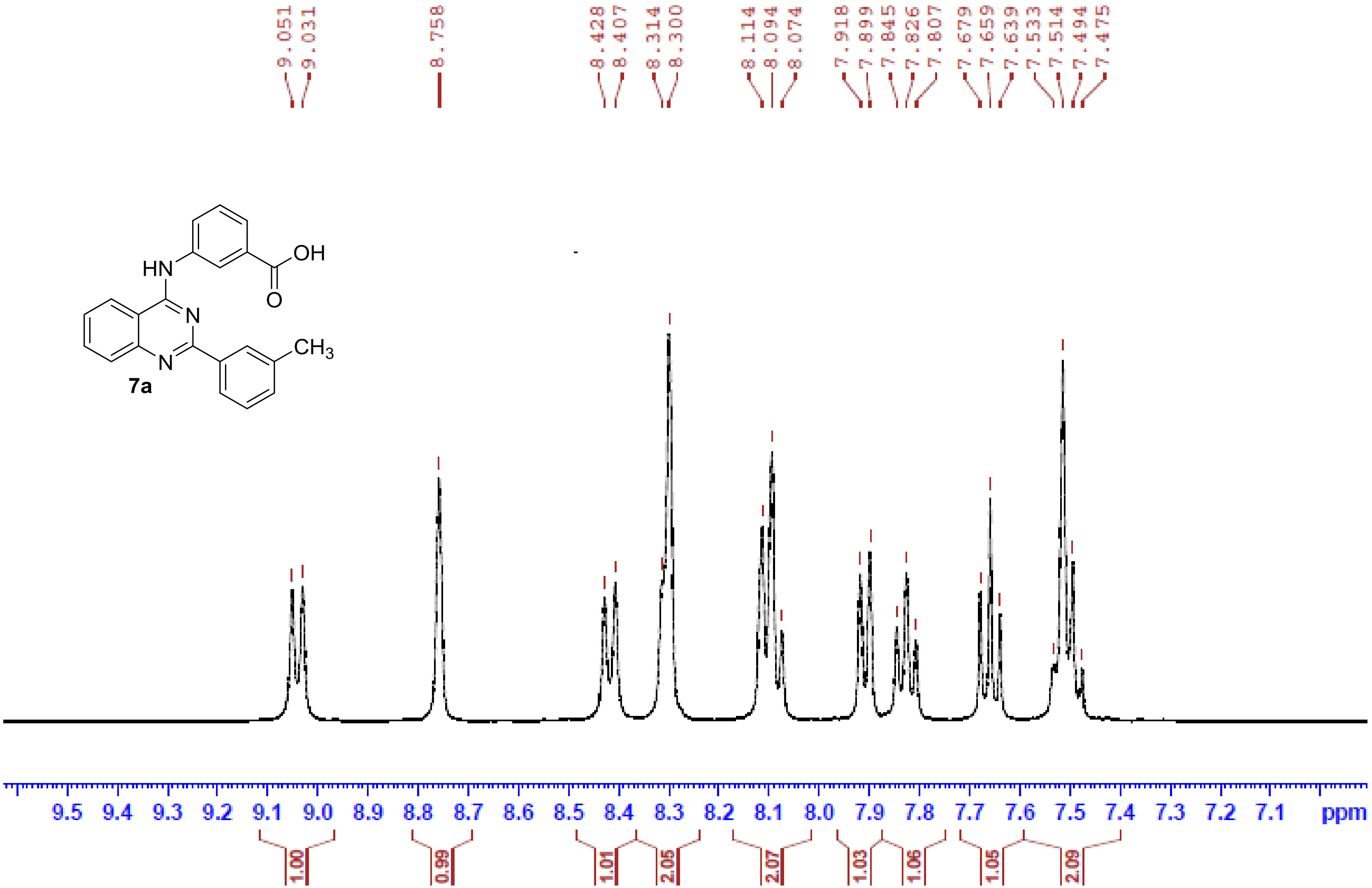

Wagdy Eldehna-Q 9b-Carbon_MS

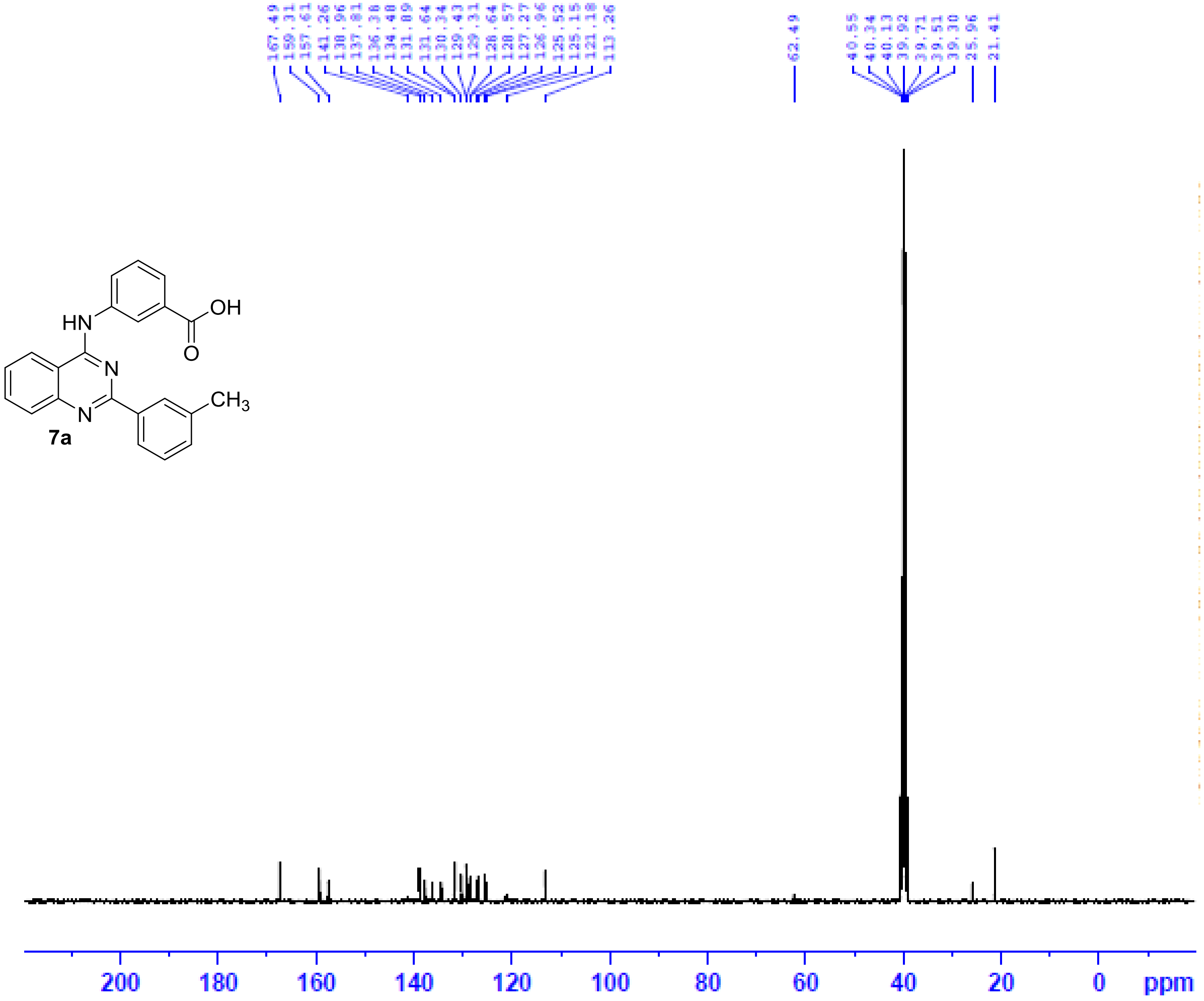

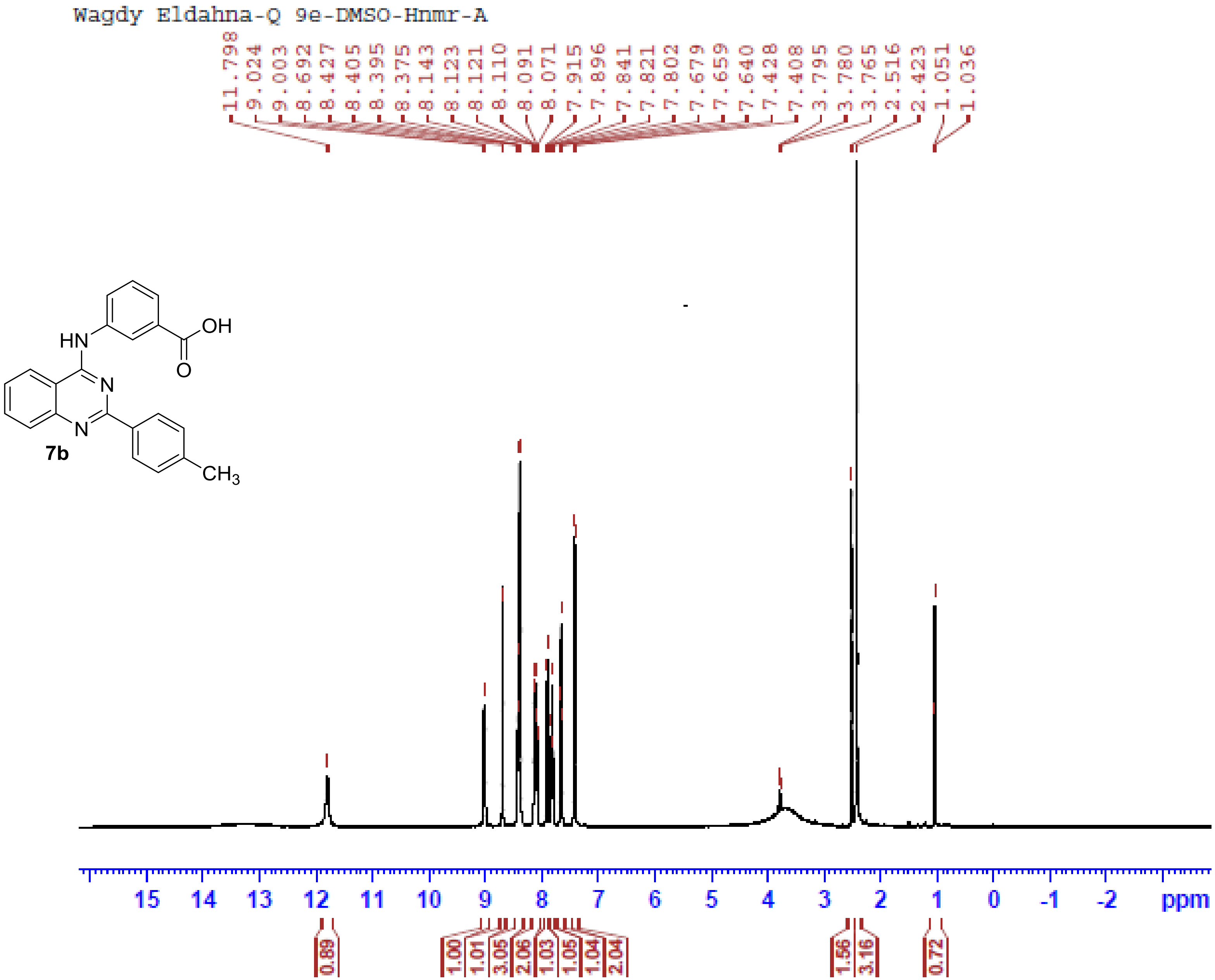
Wagdy Eldahna-Q 9e-DMSO-Hnmr-A
11.798
9.024
9.003
8.692
8.427
8.405
8.395
8.375
8.143
8.123
8.121
8.110
8.091
8.071
7.915
7.896
7.841
7.821
7.802
7.679
7.659
7.640
7.428
7.408
3.795
3.780
3.765
2.516
2.423
1.051
1.036
HN
OH
O
N
N
CH3
15 14 13 12 11 10 9 8 7 6 5 4 3 2 1 0 -1 -2 ppm
0.89
1.00
1.01
3.05
2.06
1.03
1.05
1.04
2.04
1.56
3.16
0.72
7b

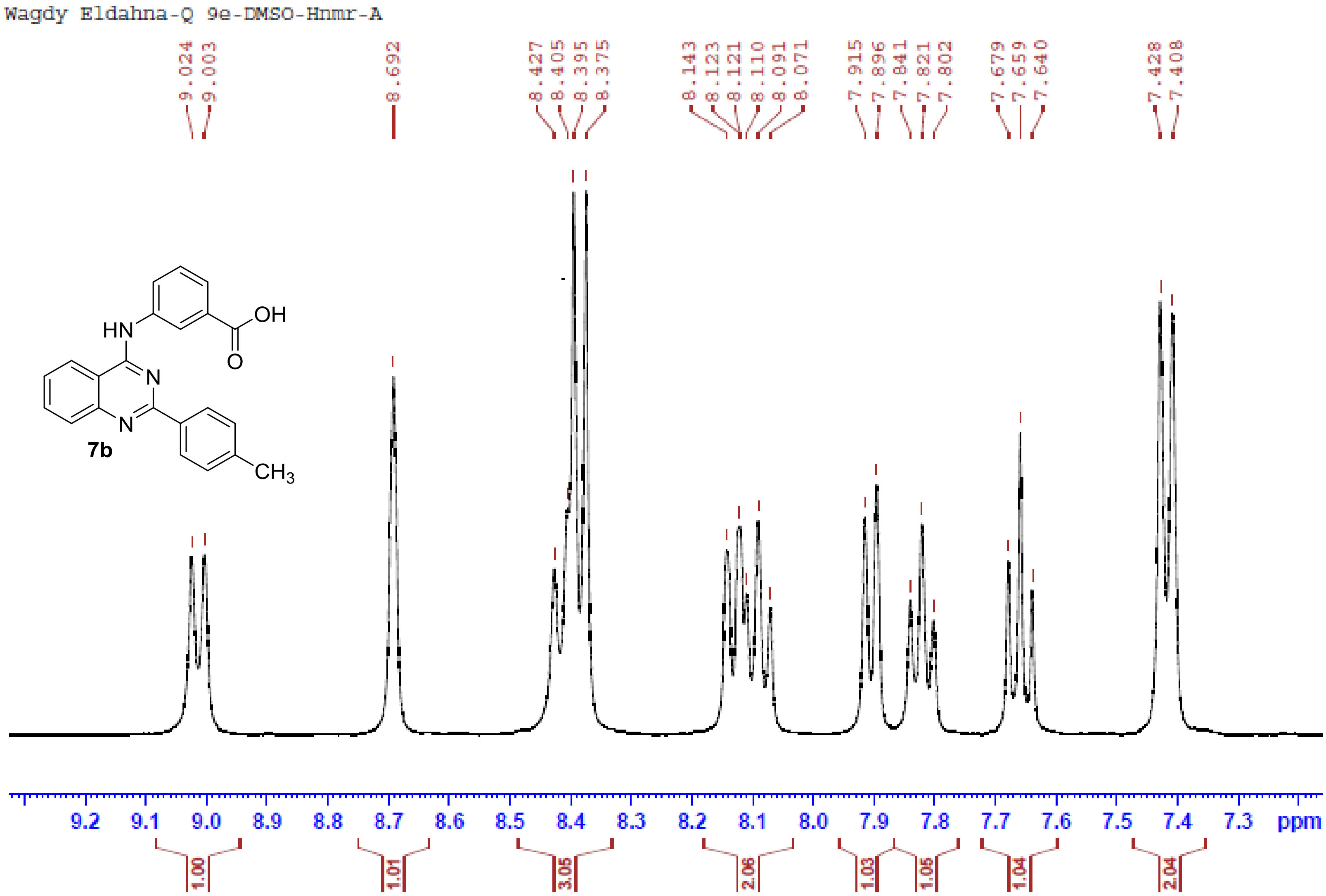

Wagdy Eldahna-Q 9e-DMSO-Hnmr-A
9.024
9.003
8.692
8.427
8.405
8.395
8.375
8.143
8.123
8.121
8.110
8.091
8.071
7.915
7.896
7.841
7.821
7.802
7.679
7.659
7.640
7.428
7.408
HN
OH
O
N
N
CH3
7b
9.2 9.1 9.0 8.9 8.8 8.7 8.6 8.5 8.4 8.3 8.2 8.1 8.0 7.9 7.8 7.7 7.6 7.5 7.4 7.3 ppm
1.00
1.01
3.05
2.06
1.03
1.05
1.04
2.04

Wagdy Eldehna-Q 9e-Carbon_MS

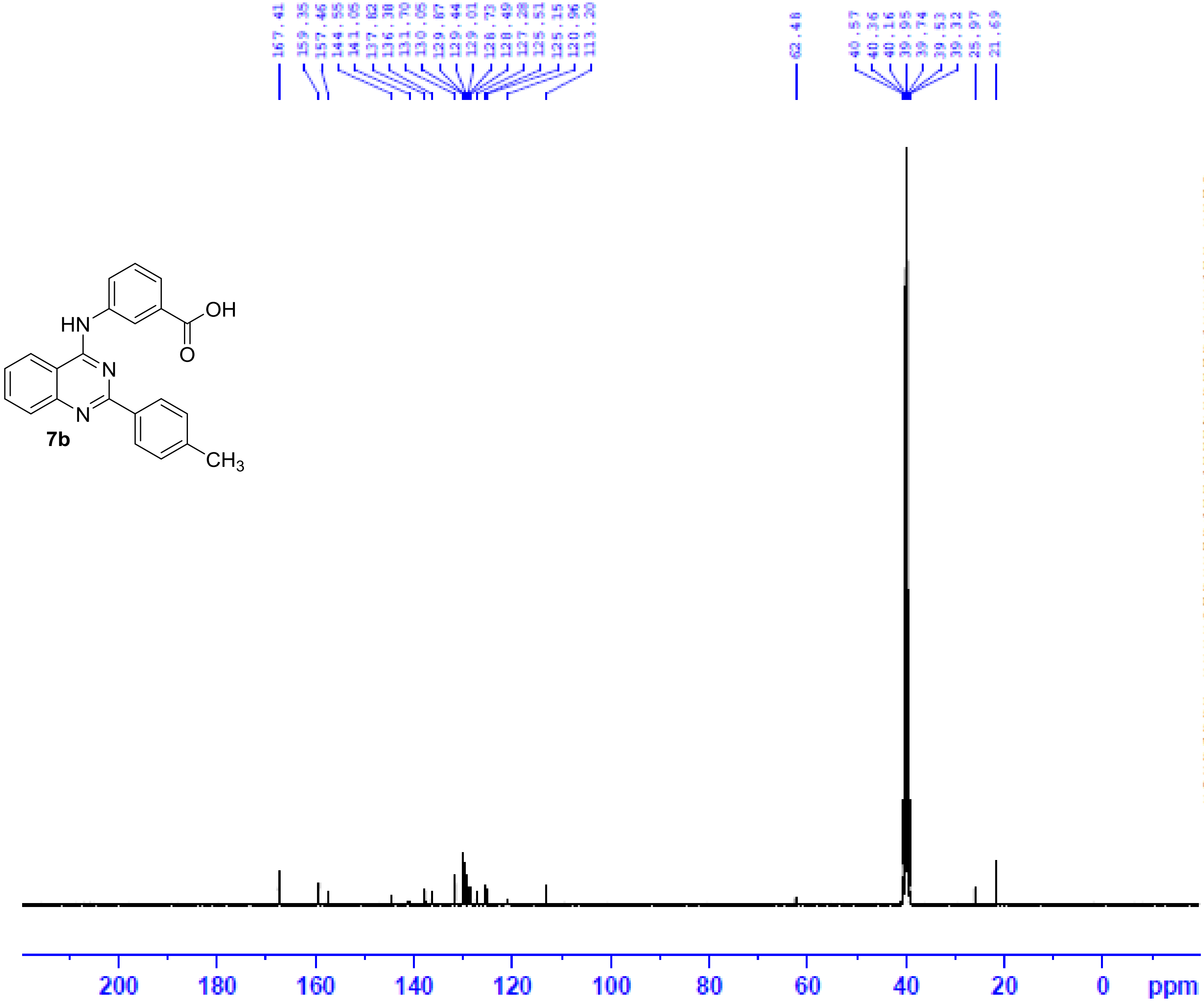

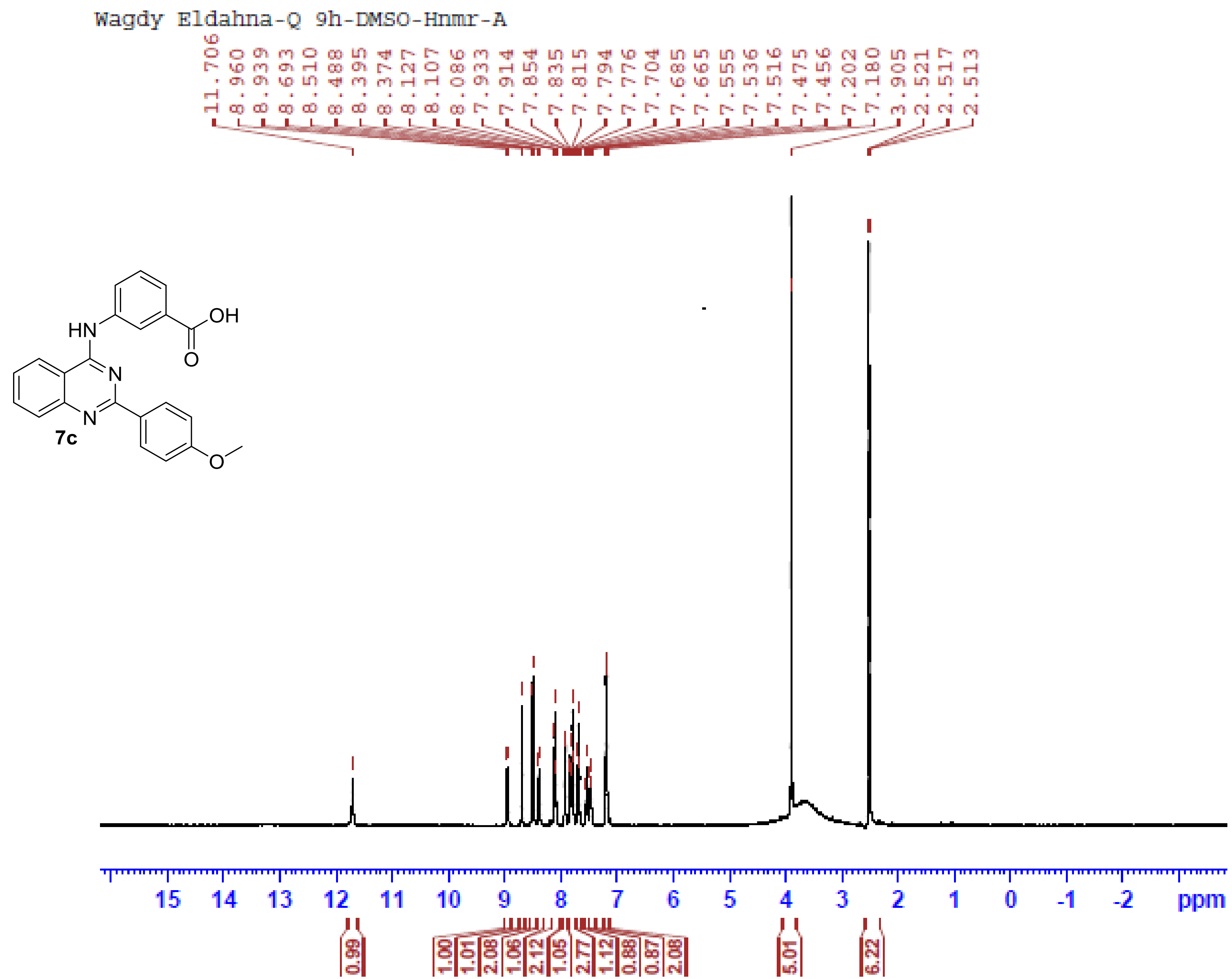
Wagdy Eldahna-Q 9h-DMSO-Hnmr-A
11.706
8.960
8.939
8.693
8.510
8.488
8.395
8.374
8.127
8.107
8.086
7.933
7.914
7.854
7.835
7.815
7.794
7.776
7.704
7.685
7.665
7.555
7.536
7.516
7.475
7.456
7.202
7.180
3.905
2.521
2.517
2.513
HN
OH
O
N
N
O
7c
15 14 13 12 11 10 9 8 7 6 5 4 3 2 1 0 -1 -2 ppm
0.99
1.00
1.01
2.08
1.06
2.12
1.05
2.77
1.12
0.88
0.87
2.08
5.01
6.22

Wagdy Eldahna-Q 9h-DMSO-Hnmr-A

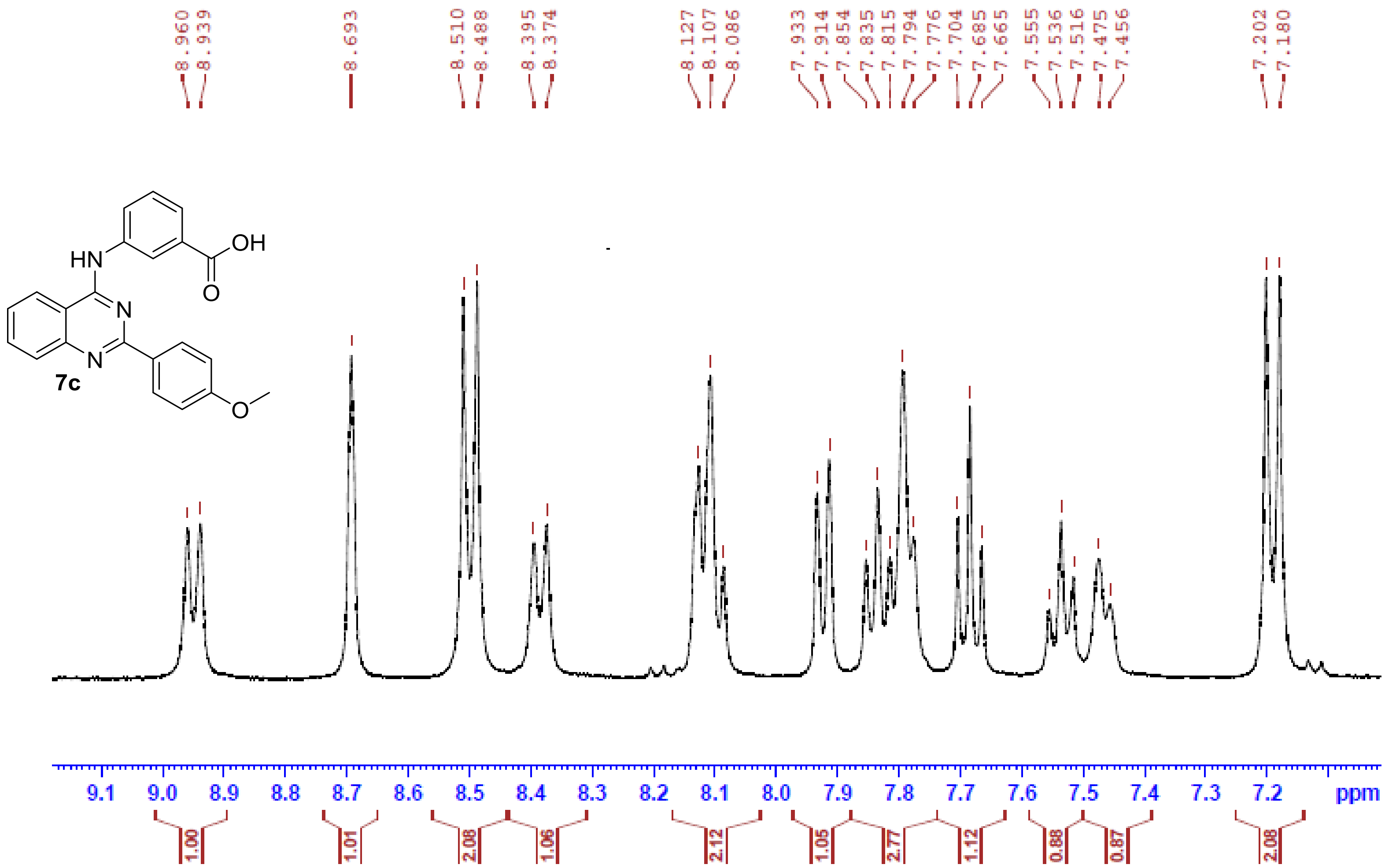

Wagdy Eldehna-Q 9h-Carbon_MS

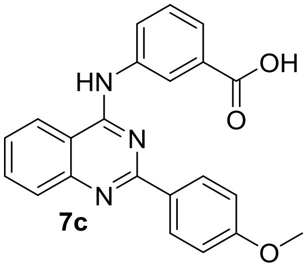

7c

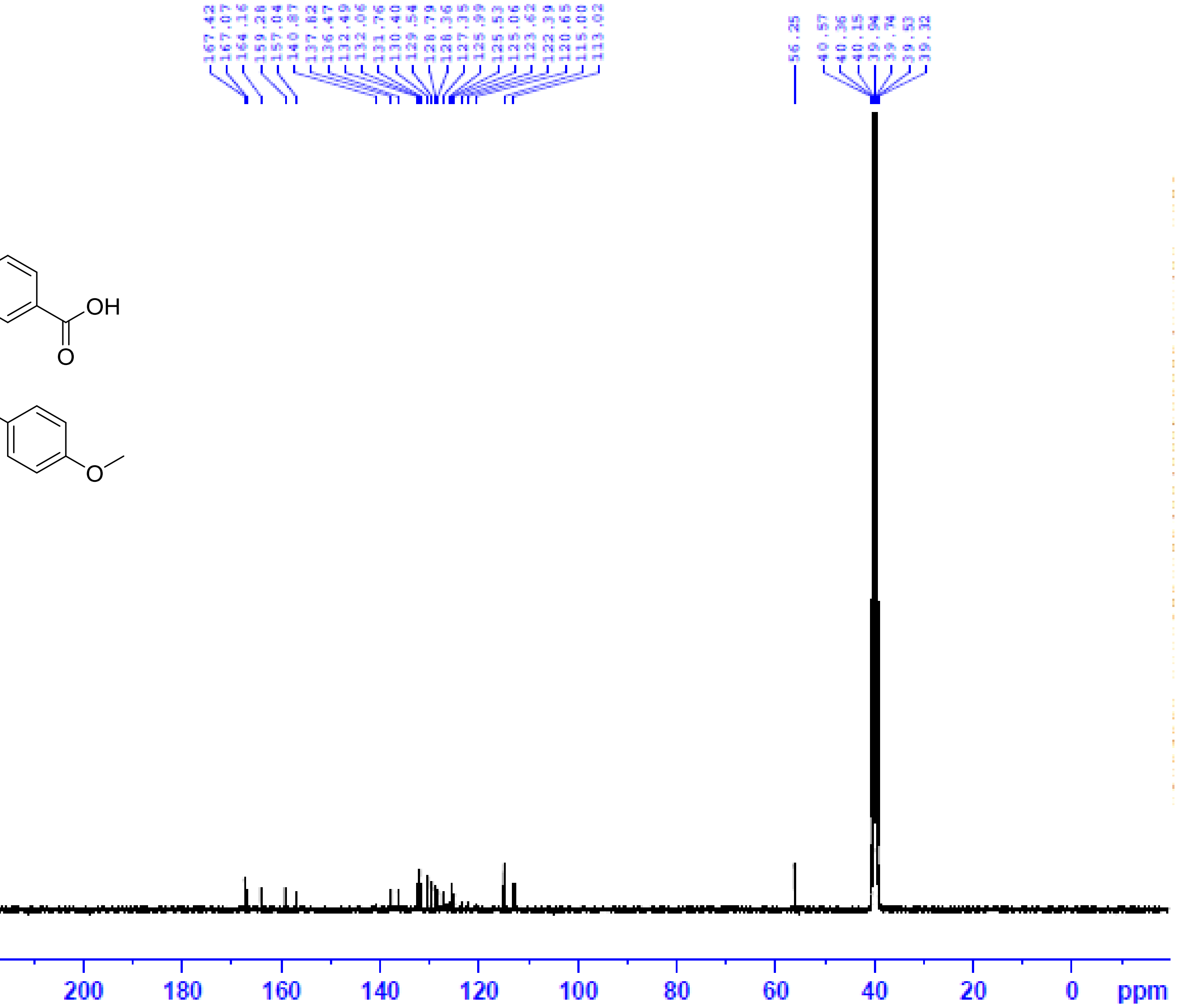

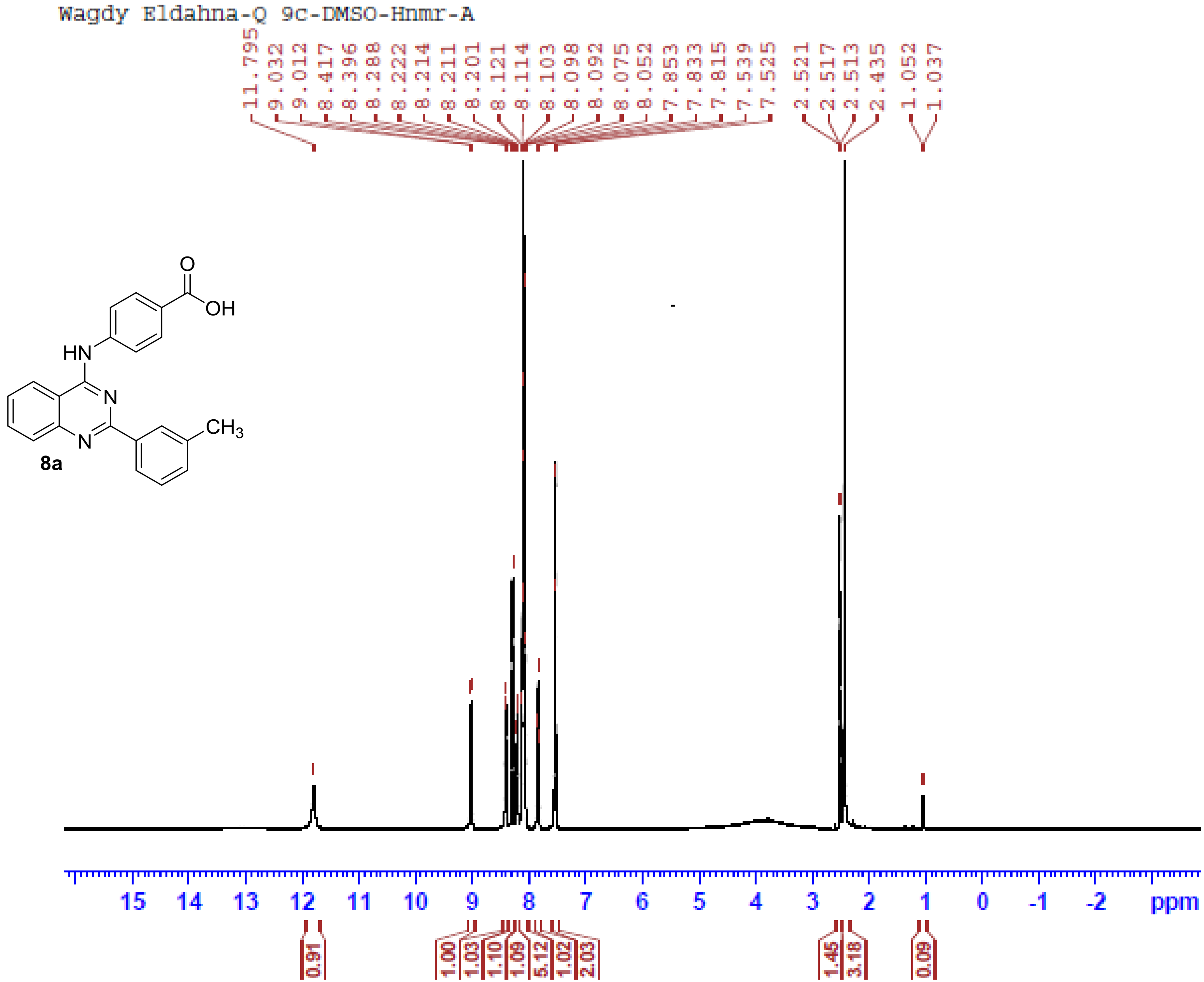
Wagdy Eldahna-Q 9c-DMSO-Hnmr-A
11.795
9.032
9.012
8.417
8.396
8.288
8.222
8.214
8.211
8.201
8.121
8.114
8.103
8.098
8.092
8.075
8.052
7.853
7.833
7.815
7.539
7.525
2.521
2.517
2.513
2.435
1.052
1.037
O
OH
HN
N
N
CH3
8a
15
14
13
12
11
10
9
8
7
6
5
4
3
2
1
0
-1
-2
ppm
0.91
1.00
1.03
1.10
1.09
5.12
1.02
2.03
1.45
3.18
0.09

Wagdy Eldahna-Q 9c-DMSO-Hnmr-A

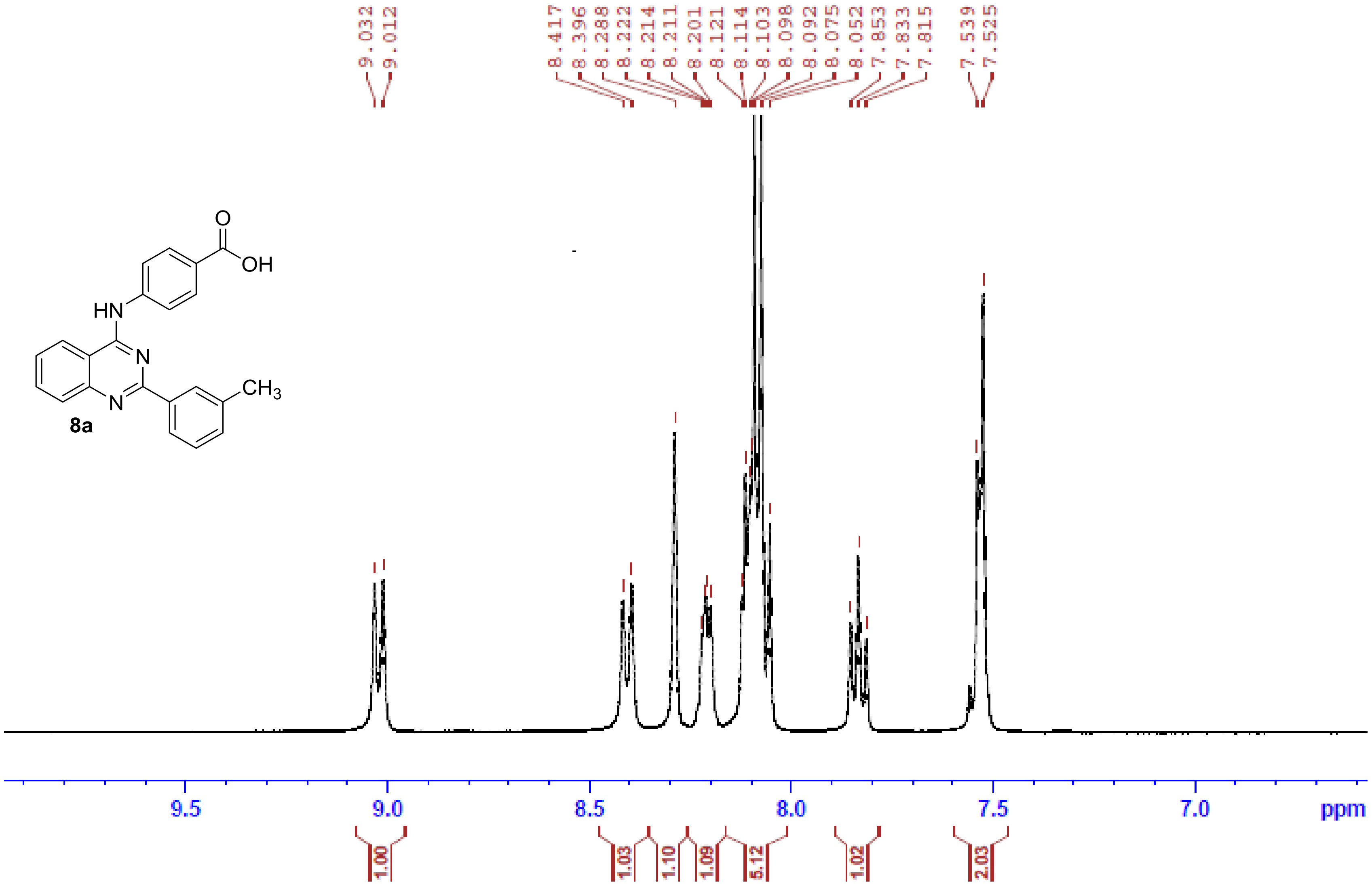

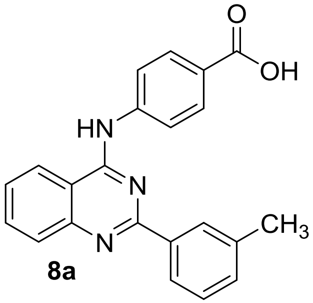
O
OH
HN
N
N
CH3

8a

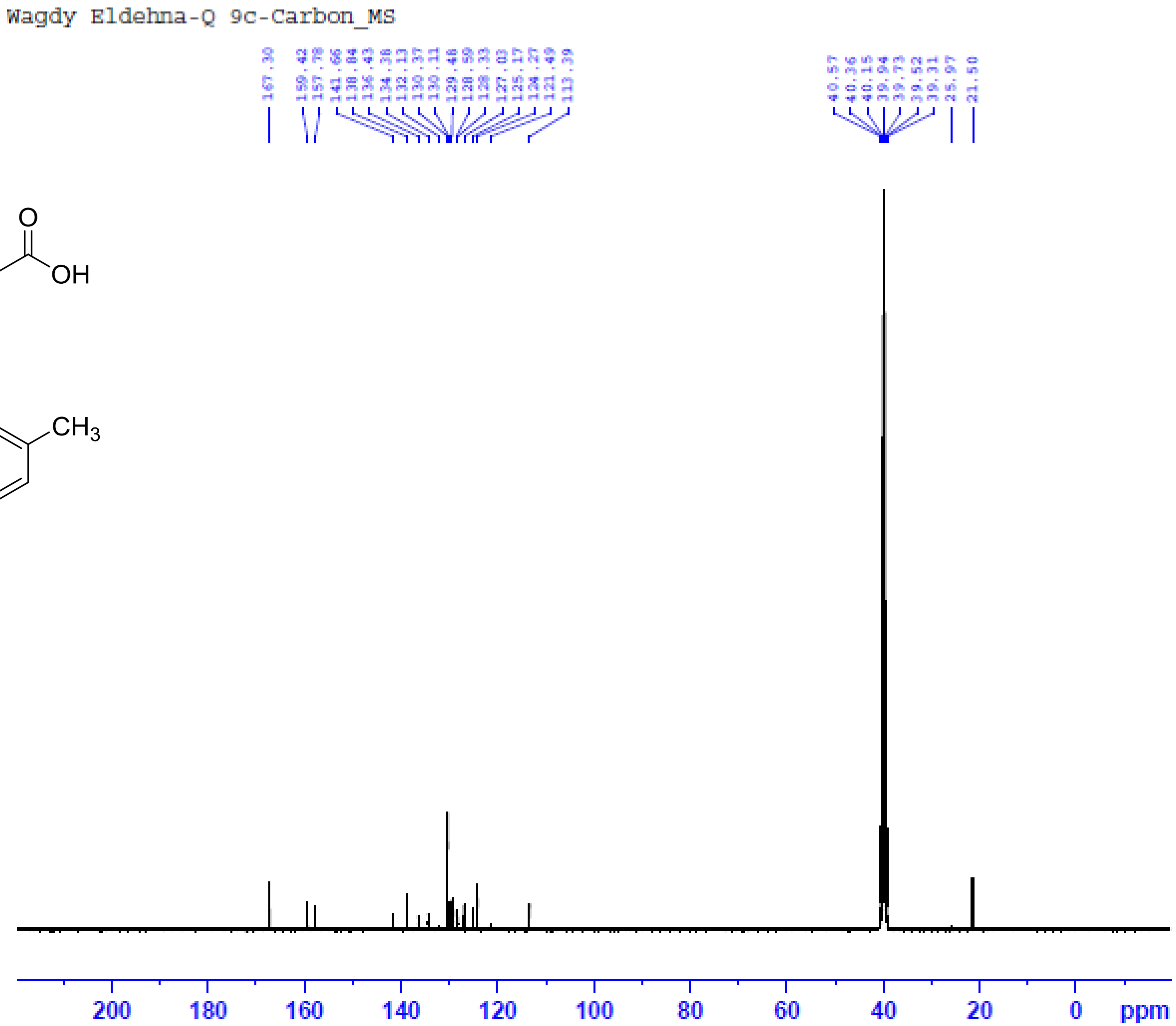
Wagdy Eldehna-Q 9c-Carbon_MS
167.30
159.42
157.78
141.66
138.84
136.43
134.38
132.13
130.37
130.11
129.48
128.59
128.33
127.03
125.17
124.27
121.49
113.39
40.57
40.36
40.15
39.94
39.73
39.52
39.31
25.97
21.50
200
180
160
140
120
100
80
60
40
20
0
ppm

Wagdy Eldahna-Q 9f-DMSO-Hnmr-A

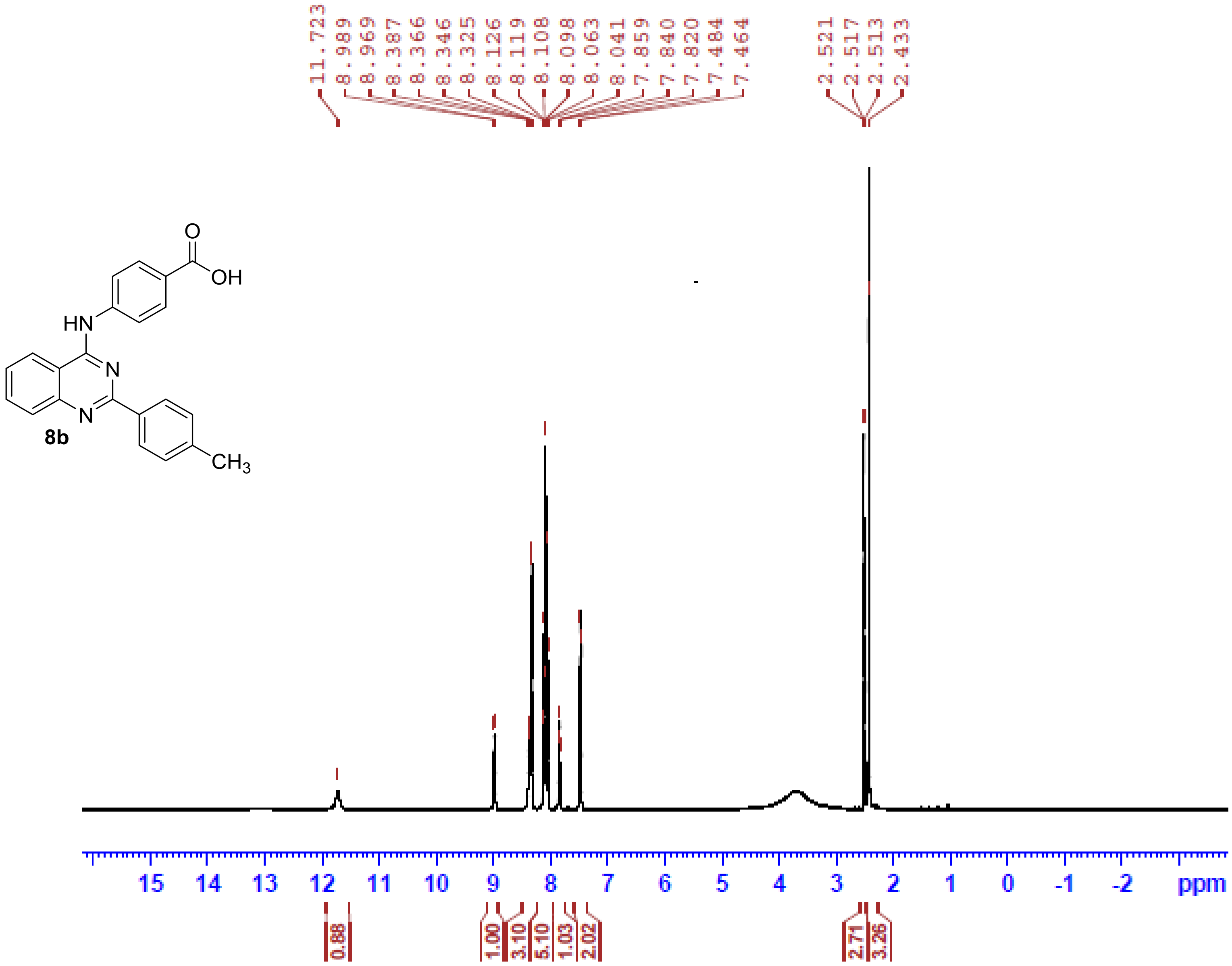

Wagdy Eldahna-Q 9f-DMSO-Hnmr-A

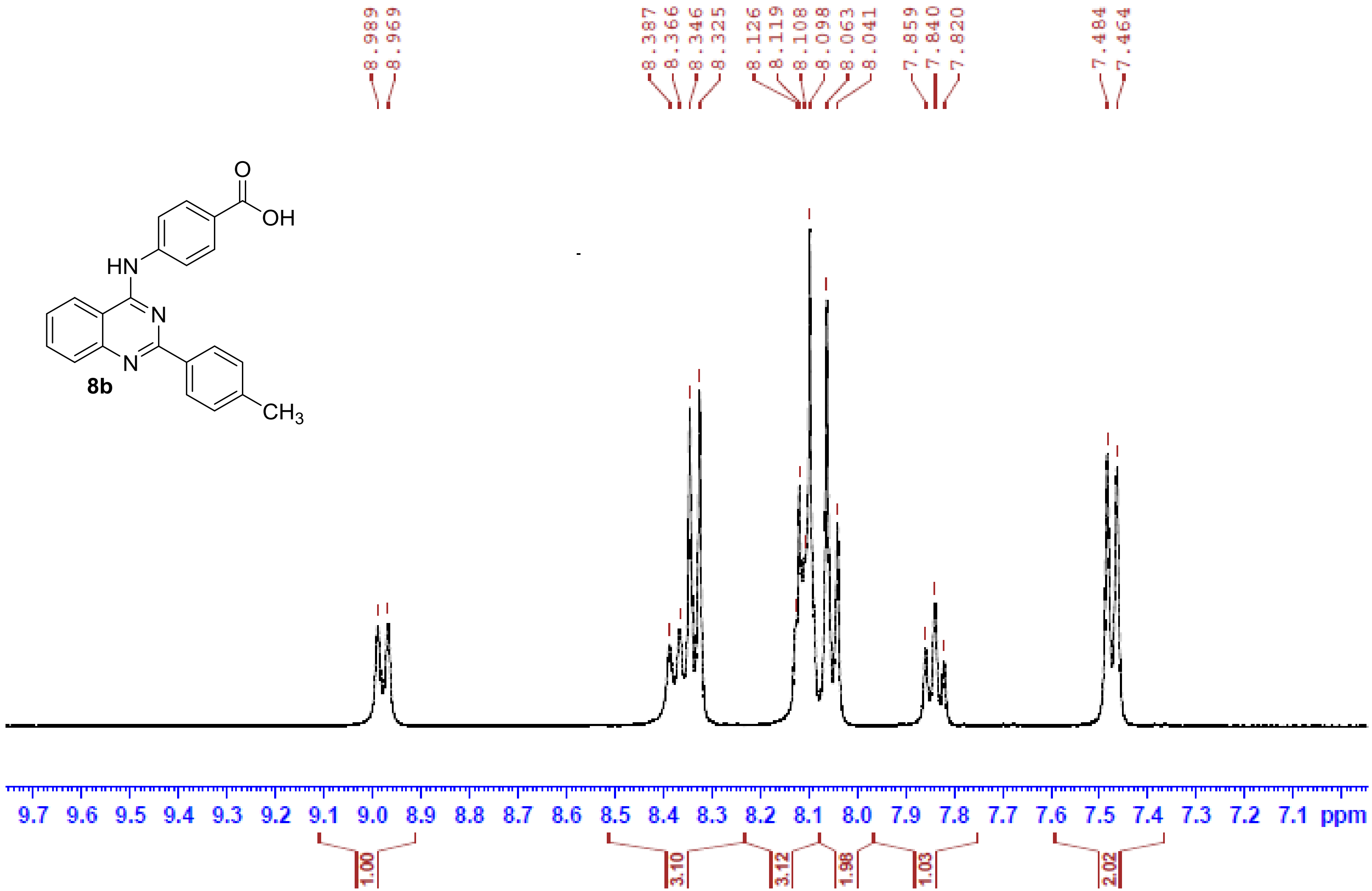

Wagdy Eldahna-Q 91-DMSO-Hnmr-A

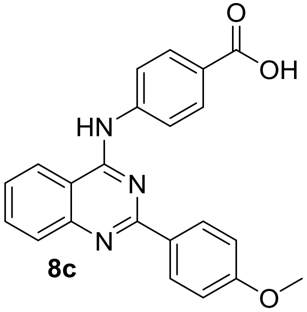

8c

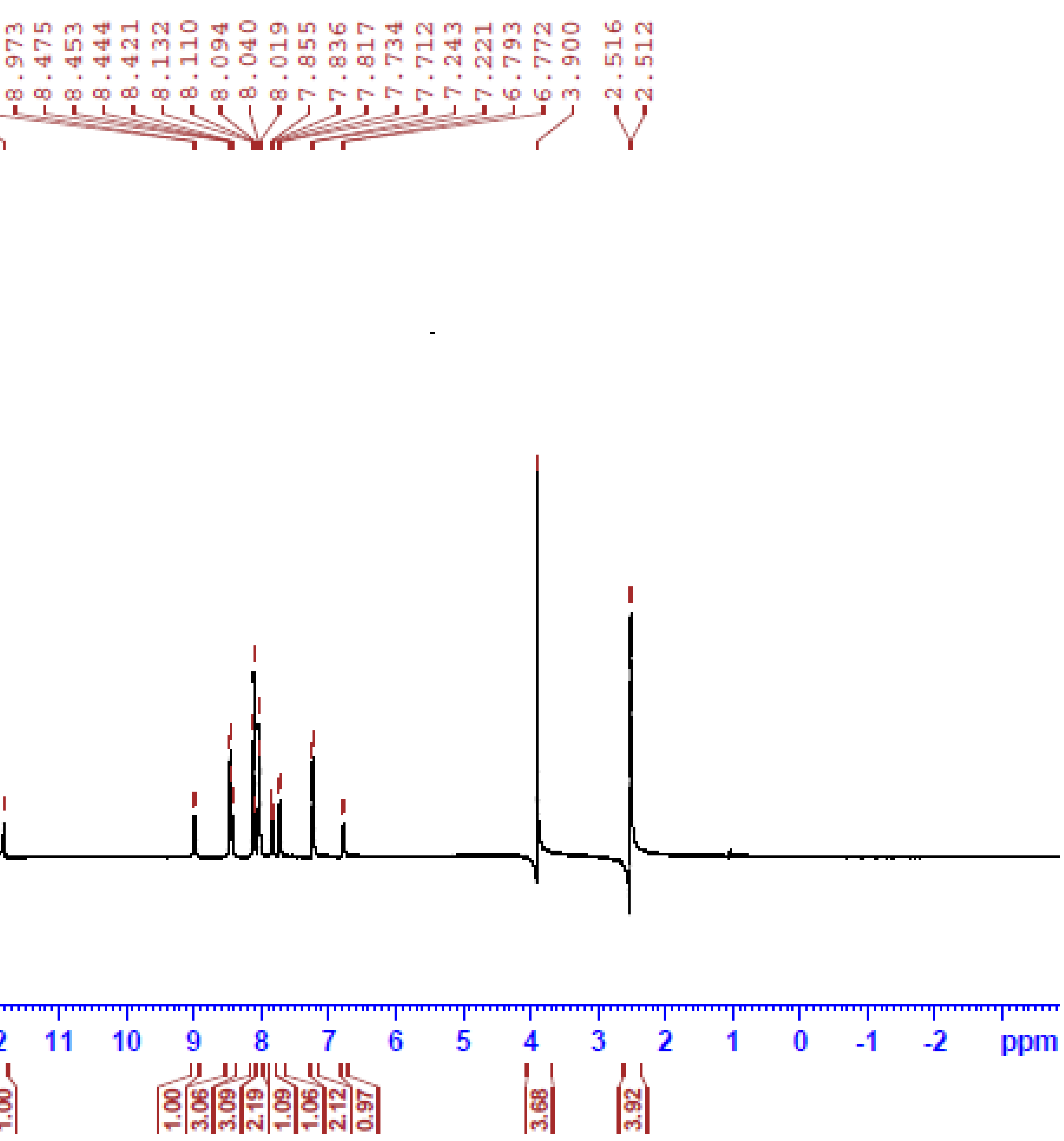

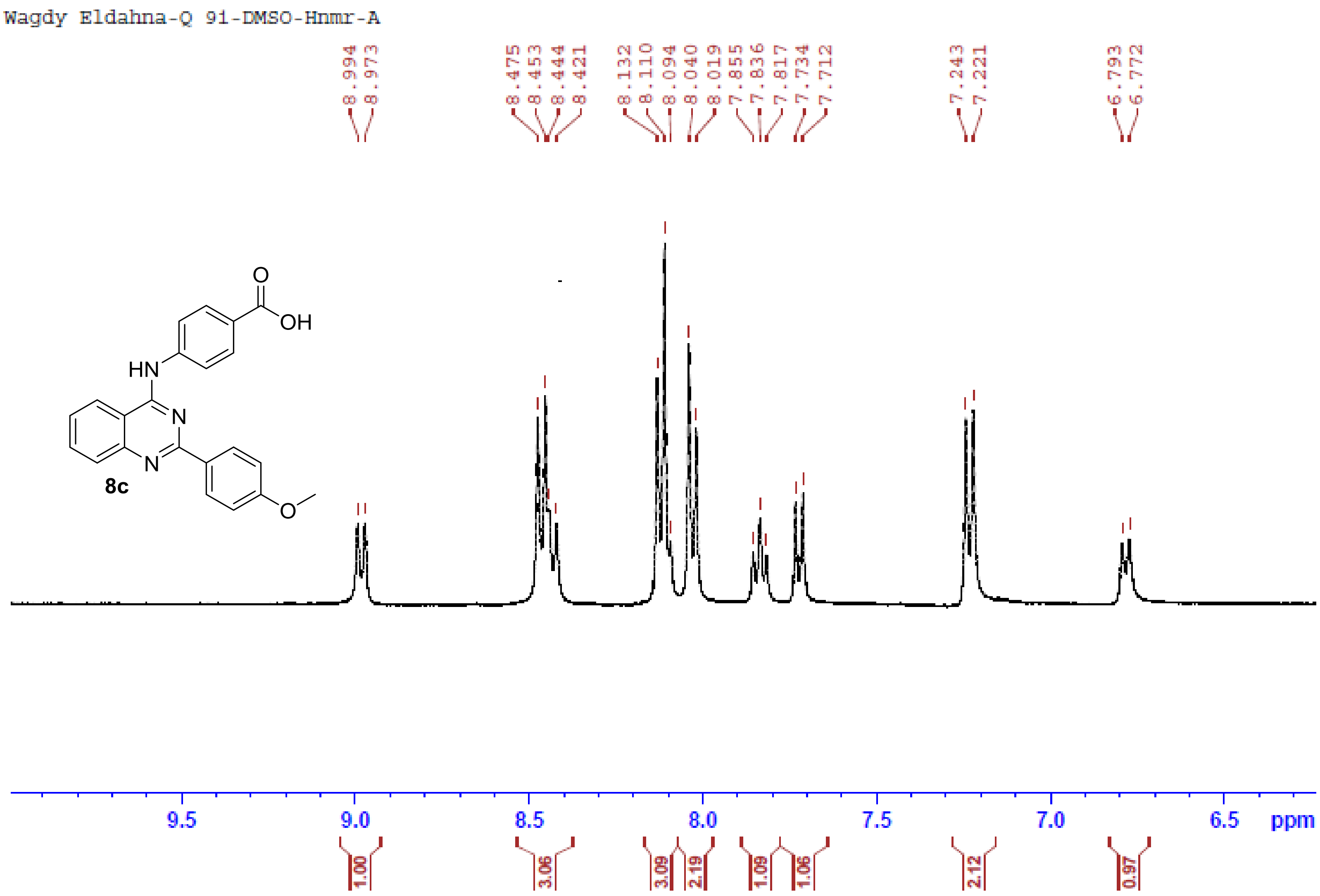
Wagdy Eldahna-Q 91-DMSO-Hnmr-A
8.994
8.973
8.475
8.453
8.444
8.421
8.132
8.110
8.094
8.040
8.019
7.855
7.836
7.817
7.734
7.712
7.243
7.221
6.793
6.772
O
OH
HN
N
N
O
8c
9.5
9.0
8.5
8.0
7.5
7.0
6.5
ppm
1.00
3.06
3.09
2.19
1.09
1.06
2.12
0.97

Wagdy Eldehna-Q 91-Carbon_MS

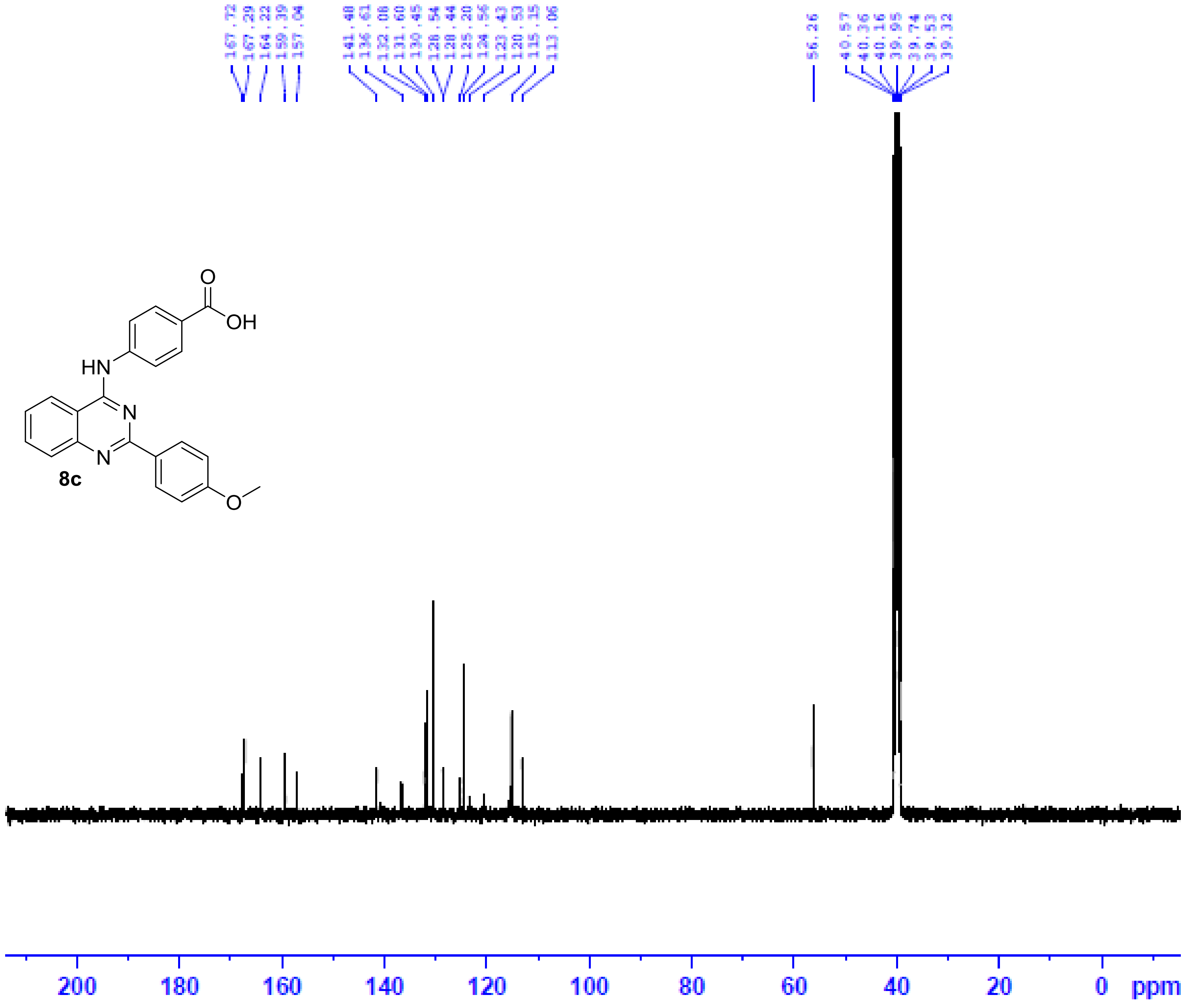

Developmental Therapeutics Program	NSC: D-835854 / 1	Conc: 1.00E-5 Molar	Test Date: Mar 07, 2022
One Dose Mean Graph	Experiment ID: 2203OS82		Report Date: Mar 25, 2022

6a

Panel/Cell Line	Growth Percent
Leukemia	
CCRF-CEM	17.83
HL-60(TB)	39.37
K-562	28.68
MOLT-4	34.28
RPMI-8226	28.59
SR	23.03
Non-Small Cell Lung Cancer	
A549/ATCC	43.63
EKVX	20.98
HOP-62	46.18
HOP-92	-5.02
NCI-H226	36.13
NCI-H23	20.79
NCI-H322M	48.74
NCI-H460	19.15
NCI-H522	59.94
Colon Cancer	
COLO 205	120.57
HCC-2998	27.10
HCT-116	22.67
HCT-15	14.29
HT29	39.66
KM12	29.97
SW-620	35.45
CNS Cancer	
SF-268	37.50
SF-295	27.07
SF-539	47.66
SNB-19	21.25
SNB-75	55.27
U251	26.61
Melanoma	
LOX IMVI	36.42
MALME-3M	37.17
M14	25.25
MDA-MB-435	44.63
SK-MEL-2	66.01
SK-MEL-28	49.95
SK-MEL-5	6.01
UACC-257	49.24
UACC-62	28.00
Ovarian Cancer	
IGROV1	59.24
OVCAR-3	34.15
OVCAR-4	41.88
OVCAR-5	77.00
OVCAR-8	60.34
NCI/ADR-RES	24.78
SK-OV-3	76.74
Renal Cancer	
786-0	25.01
A498	47.56
ACHN	36.44
CAKI-1	52.39
SN12C	40.74
TK-10	85.85
UO-31	31.01
Prostate Cancer	
PC-3	29.47
DU-145	50.47
Breast Cancer	
MCF7	23.97
MDA-MB-231/ATCC	66.08
HS 578T	16.61
BT-549	-0.68
T-47D	26.79
MDA-MB-468	-15.06
Mean	37.30
Delta	52.36
Range	135.63

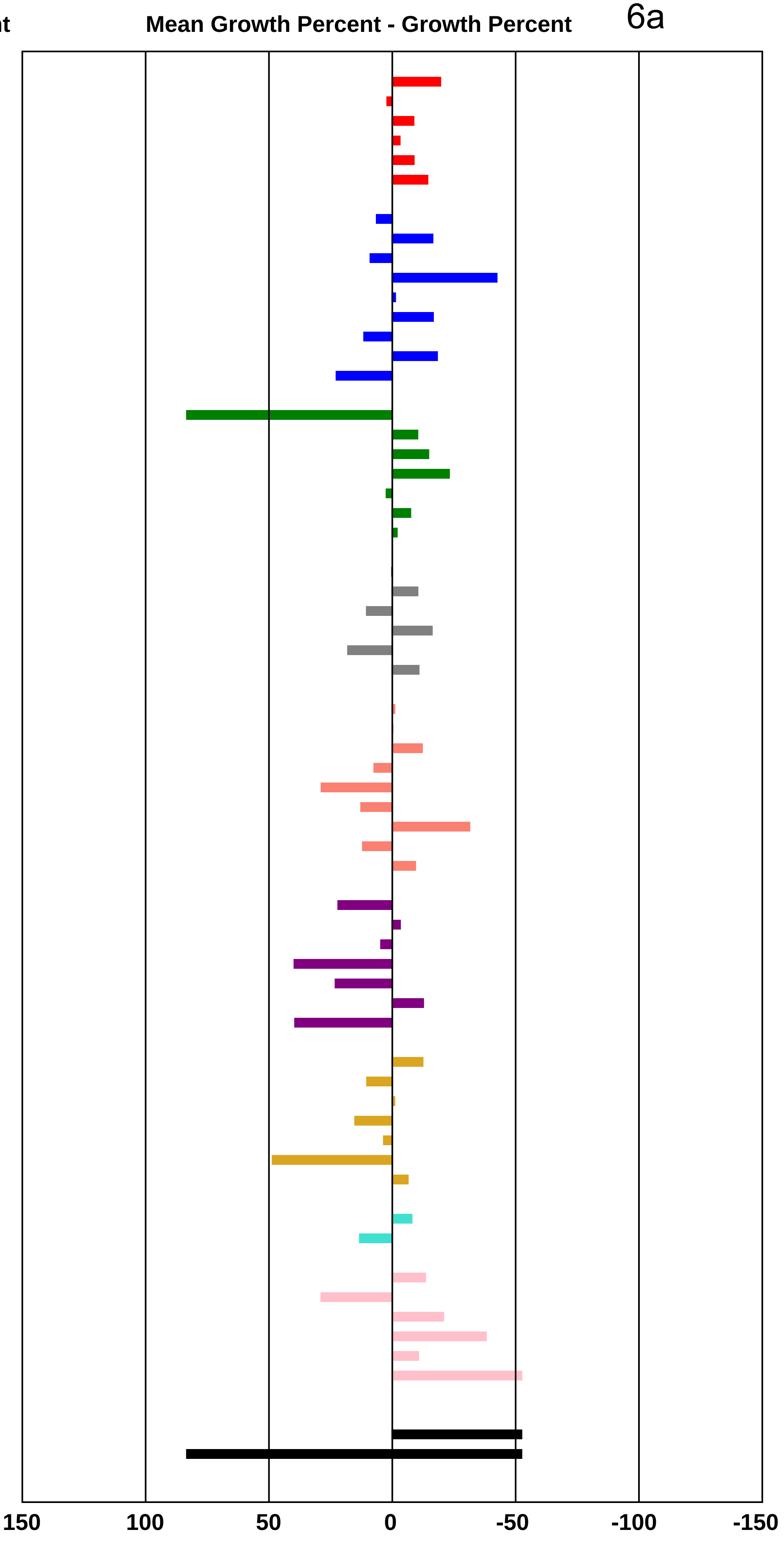

Developmental Therapeutics Program	NSC: D-835857 / 1	Conc: 1.00E-5 Molar	Test Date: Mar 07, 2022
One Dose Mean Graph	Experiment ID: 2203OS82		Report Date: Mar 25, 2022

Panel/Cell Line	Growth Percent
**Leukemia**	
CCRF-CEM	12.80
HL-60(TB)	17.80
K-562	17.64
MOLT-4	10.91
RPMI-8226	17.08
SR	18.56
**Non-Small Cell Lung Cancer**	
A549/ATCC	30.48
EKVX	13.07
HOP-62	28.29
HOP-92	-19.52
NCI-H226	15.34
NCI-H23	-4.11
NCI-H322M	33.77
NCI-H460	-9.44
NCI-H522	33.17
**Colon Cancer**	
COLO 205	80.94
HCC-2998	5.44
HCT-116	6.70
HCT-15	4.66
HT29	17.79
KM12	8.37
SW-620	14.46
**CNS Cancer**	
SF-268	11.24
SF-295	5.81
SF-539	21.26
SNB-19	8.82
SNB-75	15.71
U251	18.45
**Melanoma**	
LOX IMVI	8.57
MALME-3M	19.56
M14	12.58
MDA-MB-435	23.38
SK-MEL-2	5.75
SK-MEL-28	28.75
SK-MEL-5	-24.79
UACC-257	28.64
UACC-62	11.04
**Ovarian Cancer**	
IGROV1	31.06
OVCAR-3	6.01
OVCAR-4	21.83
OVCAR-5	65.92
OVCAR-8	30.51
NCI/ADR-RES	5.16
SK-OV-3	31.32
**Renal Cancer**	
786-0	18.67
A498	21.64
ACHN	7.89
CAKI-1	21.08
SN12C	22.24
TK-10	58.93
UO-31	12.56
**Prostate Cancer**	
PC-3	15.75
DU-145	19.37
**Breast Cancer**	
MCF7	7.70
MDA-MB-231/ATCC	35.40
HS 578T	-9.53
BT-549	-23.53
T-47D	10.72
MDA-MB-468	-26.88
Mean	15.81
Delta	42.69
Range	107.82

Mean Growth Percent - Growth Percent 6b

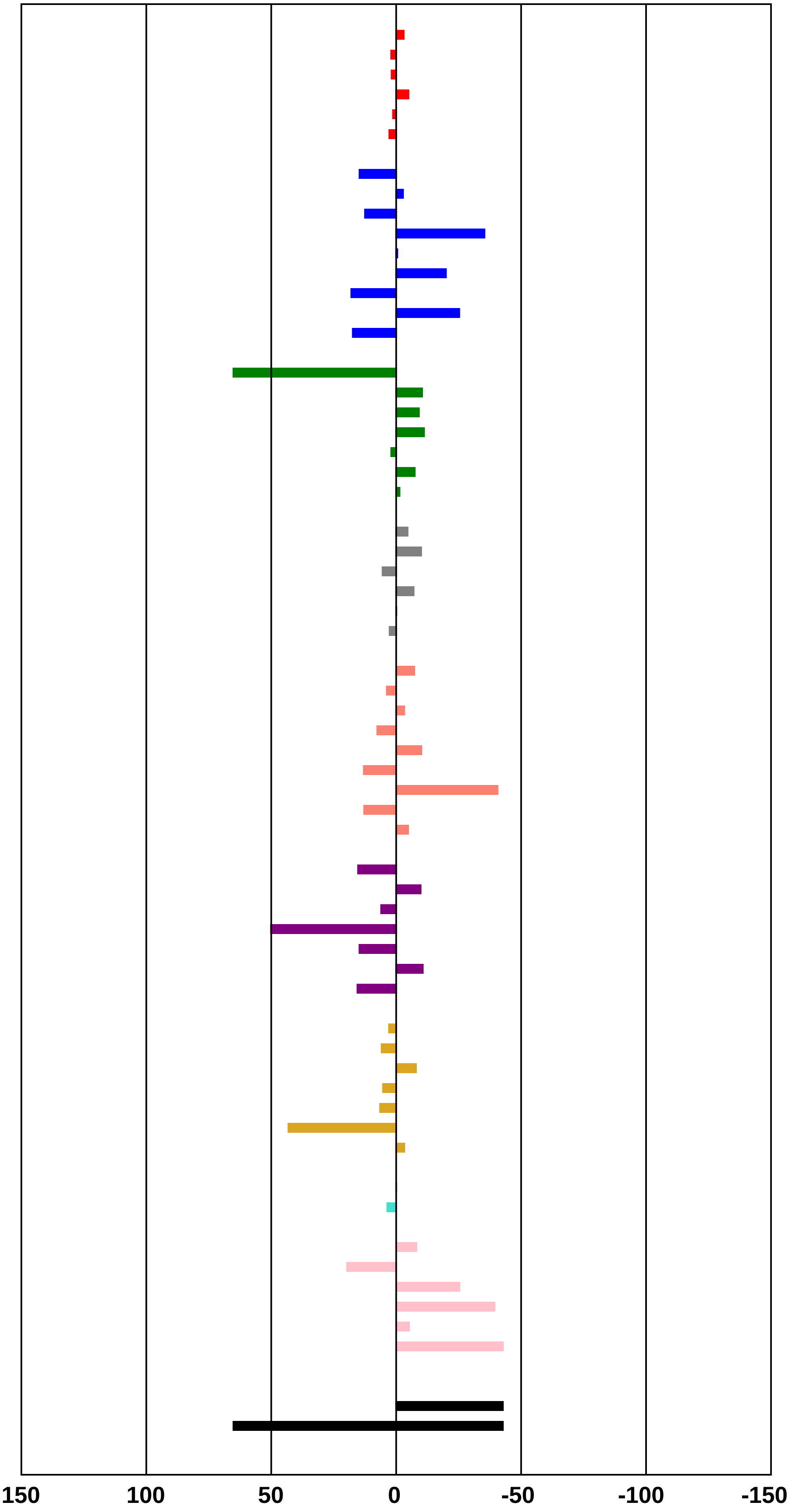

Developmental Therapeutics Program

One Dose Mean Graph

**NSC:** D-838525 / 1	**Conc:** 1.00E-5 Molar
**Test Date:** Jun 13, 2022	**Experiment ID:** 2206OS37
**Report Date:** Aug 10, 2022	

6c

Panel/Cell Line	Growth Percent
**Leukemia**	
CCRF-CEM	42.05
HL-60(TB)	32.98
K-562	31.34
MOLT-4	36.24
RPMI-8226	20.19
**Non-Small Cell Lung Cancer**	
A549/ATCC	39.61
EKVX	33.92
HOP-62	51.05
HOP-92	21.82
NCI-H226	53.45
NCI-H23	62.10
NCI-H322M	75.73
NCI-H460	11.57
NCI-H522	37.29
**Colon Cancer**	
COLO 205	81.88
HCC-2998	62.09
HCT-116	37.54
HCT-15	39.33
HT29	63.13
KM12	35.66
SW-620	39.05
**CNS Cancer**	
SF-268	56.93
SF-295	39.93
SF-539	63.06
SNB-19	45.59
SNB-75	32.48
U251	34.16
**Melanoma**	
LOX IMVI	49.53
MALME-3M	68.60
M14	52.09
MDA-MB-435	56.03
SK-MEL-2	43.49
SK-MEL-28	65.22
SK-MEL-5	30.49
UACC-257	72.03
UACC-62	55.88
**Ovarian Cancer**	
IGROV1	78.77
OVCAR-3	43.93
OVCAR-4	34.25
OVCAR-5	73.07
OVCAR-8	60.14
NCI/ADR-RES	54.40
SK-OV-3	47.19
**Renal Cancer**	
786-0	51.90
A498	58.92
ACHN	46.21
CAKI-1	46.89
RXF 393	68.31
SN12C	47.71
TK-10	74.99
**Prostate Cancer**	
PC-3	40.04
DU-145	56.86
**Breast Cancer**	
MCF7	51.24
HS 578T	28.05
BT-549	37.53
T-47D	29.31
MDA-MB-468	42.97
Mean	48.18
Delta	36.61
Range	70.31

Mean Growth Percent - Growth Percent

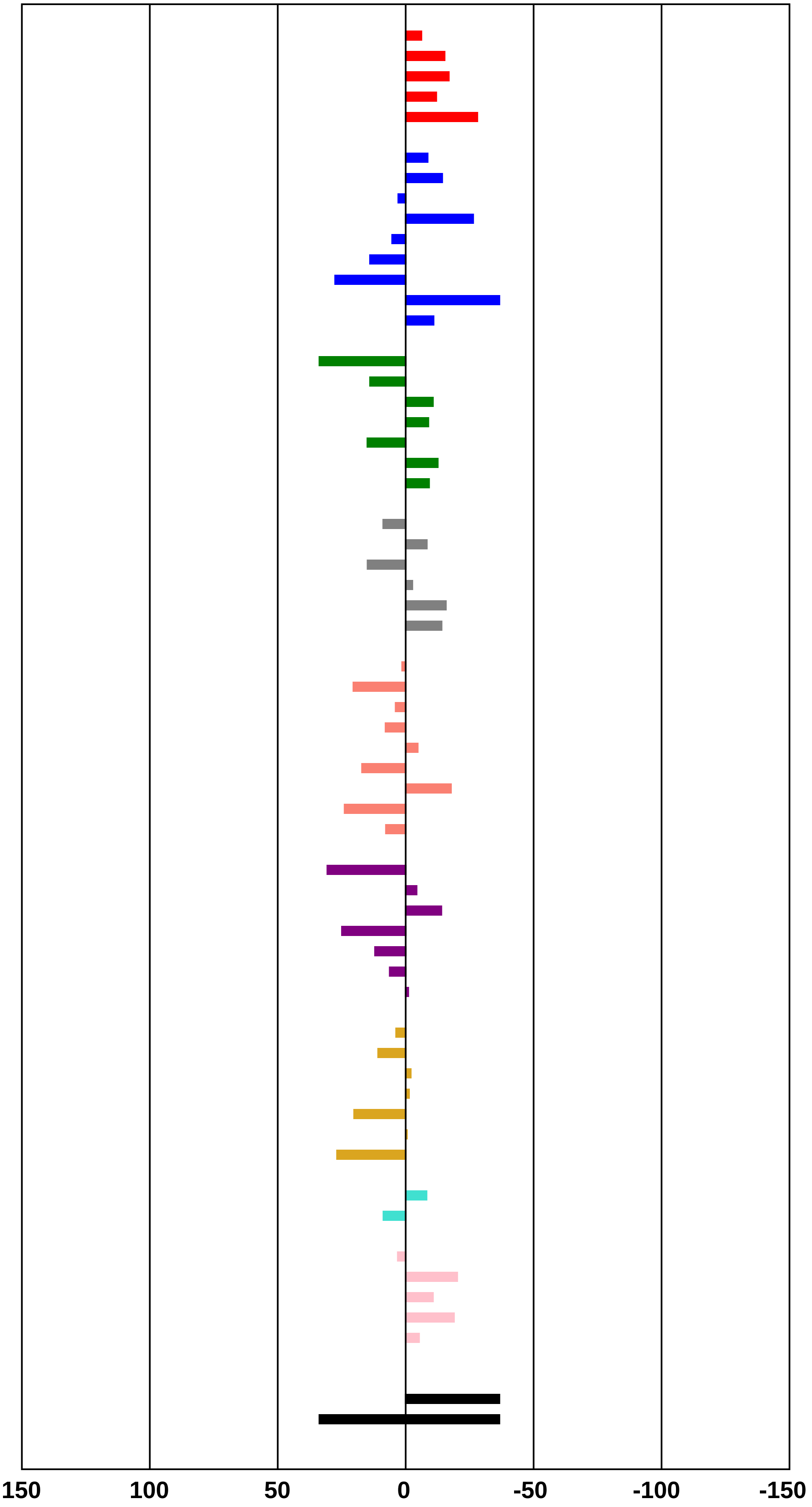

Developmental Therapeutics Program
One Dose Mean Graph

NSC: D-835858 / 1	Conc: 1.00E-5 Molar	Test Date: Mar 07, 2022
Experiment ID: 2203OS82		Report Date: Mar 25, 2022

7b

Panel/Cell Line	Growth Percent
**Leukemia**	
CCRF-CEM	78.66
HL-60(TB)	85.11
K-562	80.06
MOLT-4	89.24
RPMI-8226	70.78
SR	58.68
**Non-Small Cell Lung Cancer**	
A549/ATCC	90.82
EKVX	58.70
HOP-62	84.19
HOP-92	71.46
NCI-H226	75.32
NCI-H23	95.42
NCI-H322M	99.74
NCI-H460	97.53
NCI-H522	86.87
**Colon Cancer**	
COLO 205	124.60
HCC-2998	94.40
HCT-116	74.42
HCT-15	45.38
HT29	105.90
KM12	68.03
SW-620	94.08
**CNS Cancer**	
SF-268	69.32
SF-295	65.90
SF-539	73.18
SNB-19	71.76
SNB-75	91.06
U251	78.70
**Melanoma**	
LOX IMVI	81.15
MALME-3M	96.44
M14	83.59
MDA-MB-435	100.82
SK-MEL-2	112.04
SK-MEL-28	97.02
SK-MEL-5	85.67
UACC-257	111.51
UACC-62	87.74
**Ovarian Cancer**	
IGROV1	107.30
OVCAR-3	85.56
OVCAR-4	77.91
OVCAR-5	107.97
OVCAR-8	91.56
NCI/ADR-RES	89.49
SK-OV-3	89.56
**Renal Cancer**	
786-0	76.25
A498	95.15
ACHN	76.34
CAKI-1	82.90
SN12C	76.64
TK-10	115.15
UO-31	48.97
**Prostate Cancer**	
PC-3	80.52
DU-145	96.55
**Breast Cancer**	
MCF7	85.28
MDA-MB-231/ATCC	95.55
HS 578T	65.83
BT-549	101.46
T-47D	83.81
MDA-MB-468	116.40
Mean	86.13
Delta	40.75
Range	79.22

Mean Growth Percent - Growth Percent

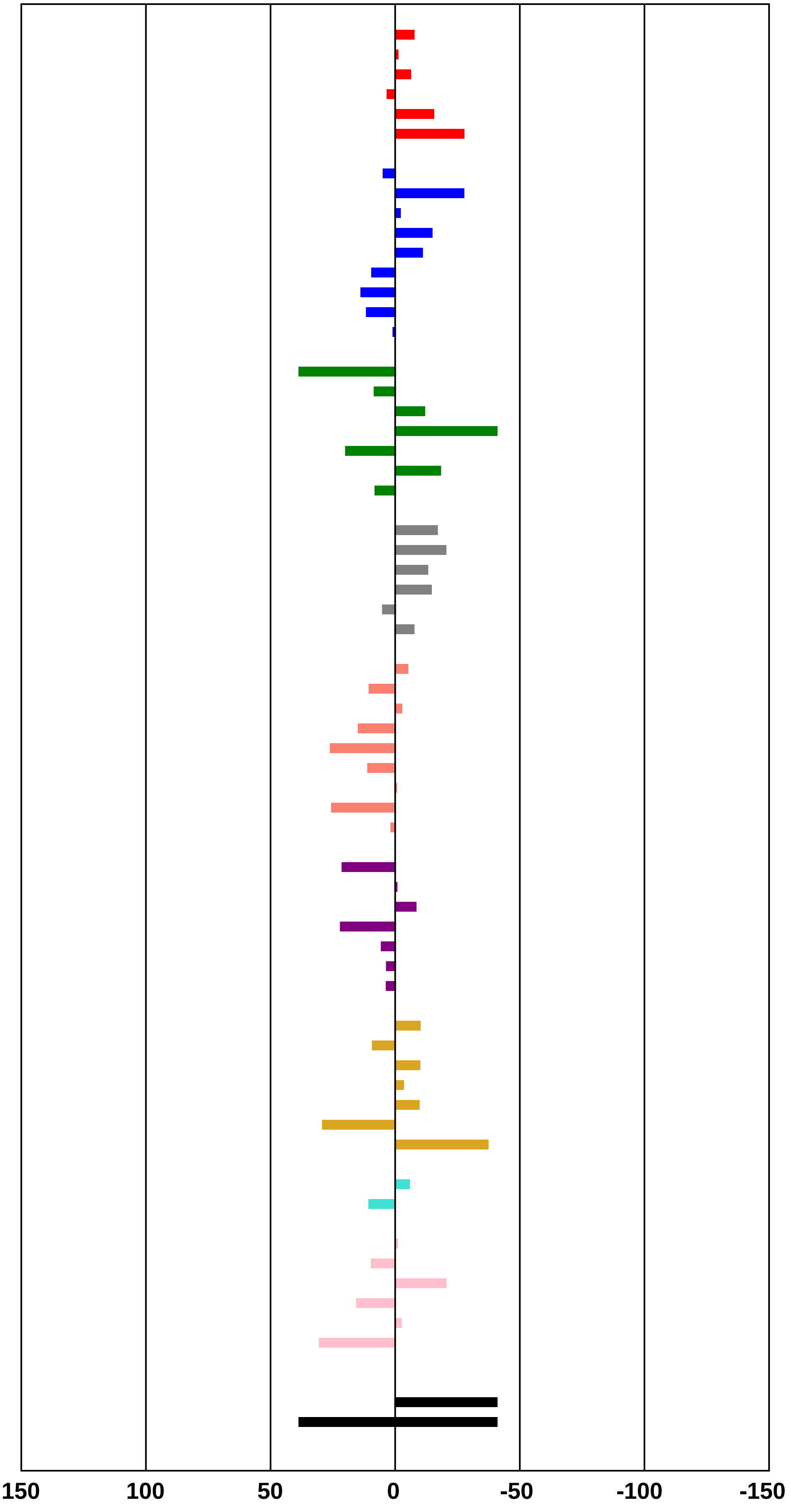

Developmental Therapeutics Program

One Dose Mean Graph

NSC: D-835856 / 1	Conc: 1.00E-5 Molar	Test Date: Mar 07, 2022
Experiment ID: 2203OS82		Report Date: Mar 25, 2022

8a

Panel/Cell Line	Growth Percent
Leukemia	
CCRF-CEM	74.58
HL-60(TB)	92.72
K-562	80.44
MOLT-4	78.39
RPMI-8226	70.27
SR	67.75
Non-Small Cell Lung Cancer	
A549/ATCC	78.30
EKVX	82.93
HOP-62	95.14
HOP-92	71.13
NCI-H226	47.62
NCI-H23	99.04
NCI-H322M	103.28
NCI-H460	78.01
NCI-H522	87.55
Colon Cancer	
COLO 205	135.98
HCC-2998	75.96
HCT-116	81.80
HCT-15	70.54
HT29	91.72
KM12	76.63
SW-620	84.27
CNS Cancer	
SF-268	62.51
SF-295	63.92
SF-539	91.50
SNB-19	67.24
SNB-75	100.40
U251	72.61
Melanoma	
LOX IMVI	71.49
MALME-3M	110.61
M14	88.98
MDA-MB-435	96.58
SK-MEL-2	123.11
SK-MEL-28	96.84
SK-MEL-5	71.03
UACC-257	101.78
UACC-62	104.52
Ovarian Cancer	
IGROV1	110.06
OVCAR-3	82.69
OVCAR-4	83.42
OVCAR-5	133.93
OVCAR-8	82.44
NCI/ADR-RES	84.58
SK-OV-3	108.70
Renal Cancer	
786-0	70.77
A498	45.68
ACHN	85.41
CAKI-1	94.53
SN12C	58.07
TK-10	91.50
UO-31	45.85
Prostate Cancer	
PC-3	83.31
DU-145	87.83
Breast Cancer	
MCF7	88.63
MDA-MB-231/ATCC	93.31
HS 578T	51.51
BT-549	66.04
T-47D	77.59
MDA-MB-468	113.23
Mean	84.51
Delta	38.83
Range	90.30

Mean Growth Percent - Growth Percent

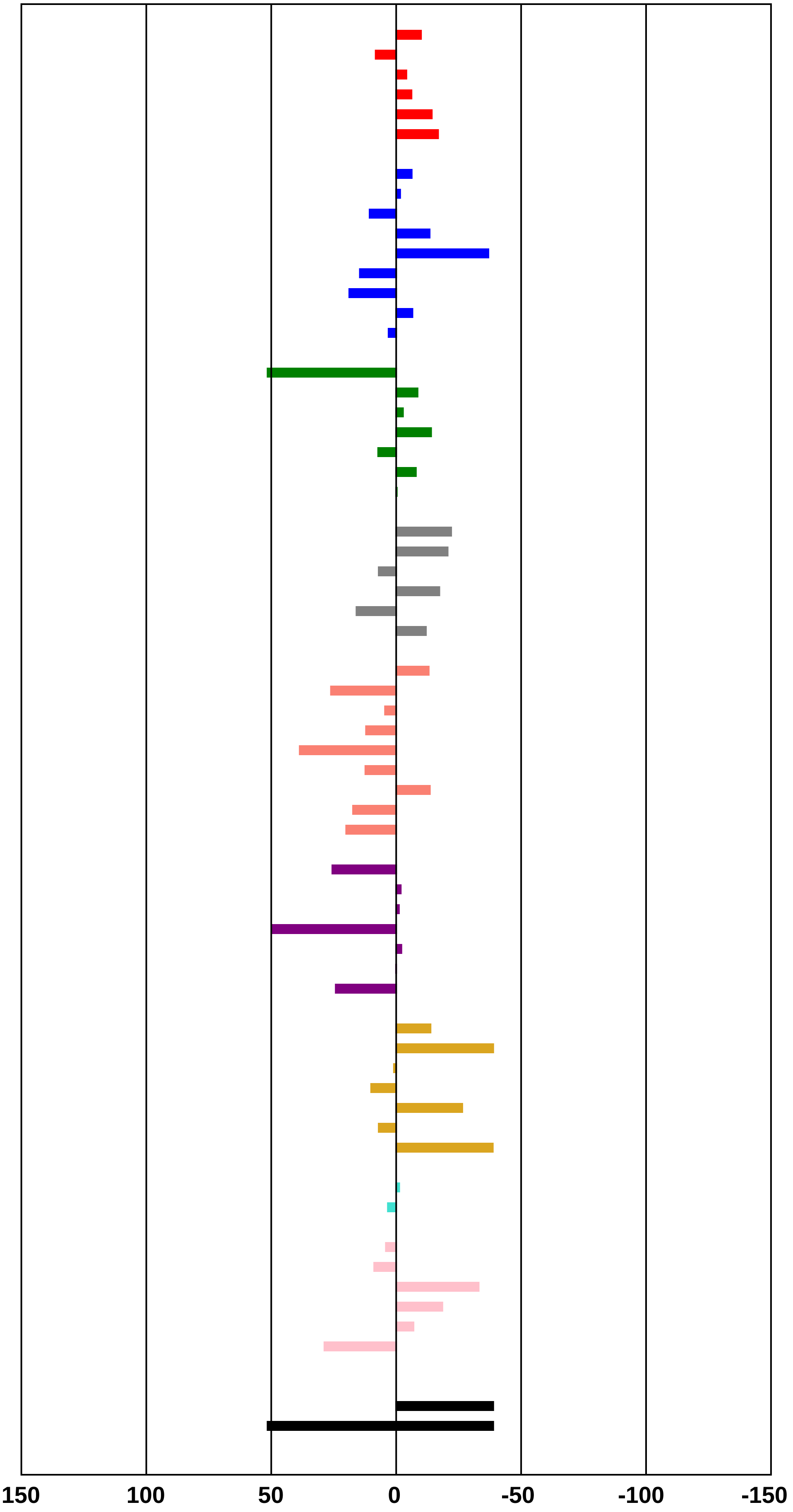

Developmental Therapeutics Program	NSC: D-835859 / 1	Conc: 1.00E-5 Molar	Test Date: Mar 07, 2022
One Dose Mean Graph	Experiment ID: 2203OS82		Report Date: Mar 25, 2022

Panel/Cell Line	Growth Percent
**Leukemia**	
CCRF-CEM	71.96
HL-60(TB)	76.30
K-562	76.80
MOLT-4	76.42
RPMI-8226	55.07
SR	61.50
**Non-Small Cell Lung Cancer**	
A549/ATCC	95.03
EKVX	72.41
HOP-62	88.98
HOP-92	75.22
NCI-H226	37.77
NCI-H23	87.14
NCI-H322M	84.20
NCI-H460	82.06
NCI-H522	91.02
**Colon Cancer**	
COLO 205	109.50
HCC-2998	72.45
HCT-116	77.19
HCT-15	50.85
HT29	87.37
KM12	57.97
SW-620	91.47
**CNS Cancer**	
SF-268	70.34
SF-295	57.85
SF-539	78.63
SNB-19	70.92
SNB-75	95.80
U251	86.94
**Melanoma**	
LOX IMVI	57.05
MALME-3M	101.05
M14	80.88
MDA-MB-435	100.06
SK-MEL-2	113.45
SK-MEL-28	84.51
SK-MEL-5	71.66
UACC-257	107.52
UACC-62	95.83
**Ovarian Cancer**	
IGROV1	92.17
OVCAR-3	79.51
OVCAR-4	74.11
OVCAR-5	95.98
OVCAR-8	81.90
NCI/ADR-RES	76.55
SK-OV-3	99.63
**Renal Cancer**	
786-0	85.63
A498	112.03
ACHN	75.90
CAKI-1	88.42
SN12C	48.99
TK-10	113.38
UO-31	41.73
**Prostate Cancer**	
PC-3	77.04
DU-145	81.93
**Breast Cancer**	
MCF7	63.19
MDA-MB-231/ATCC	87.83
HS 578T	67.76
BT-549	59.16
T-47D	68.59
MDA-MB-468	108.74
Mean	80.19
Delta	42.42
Range	75.68

Mean Growth Percent - Growth Percent 8b

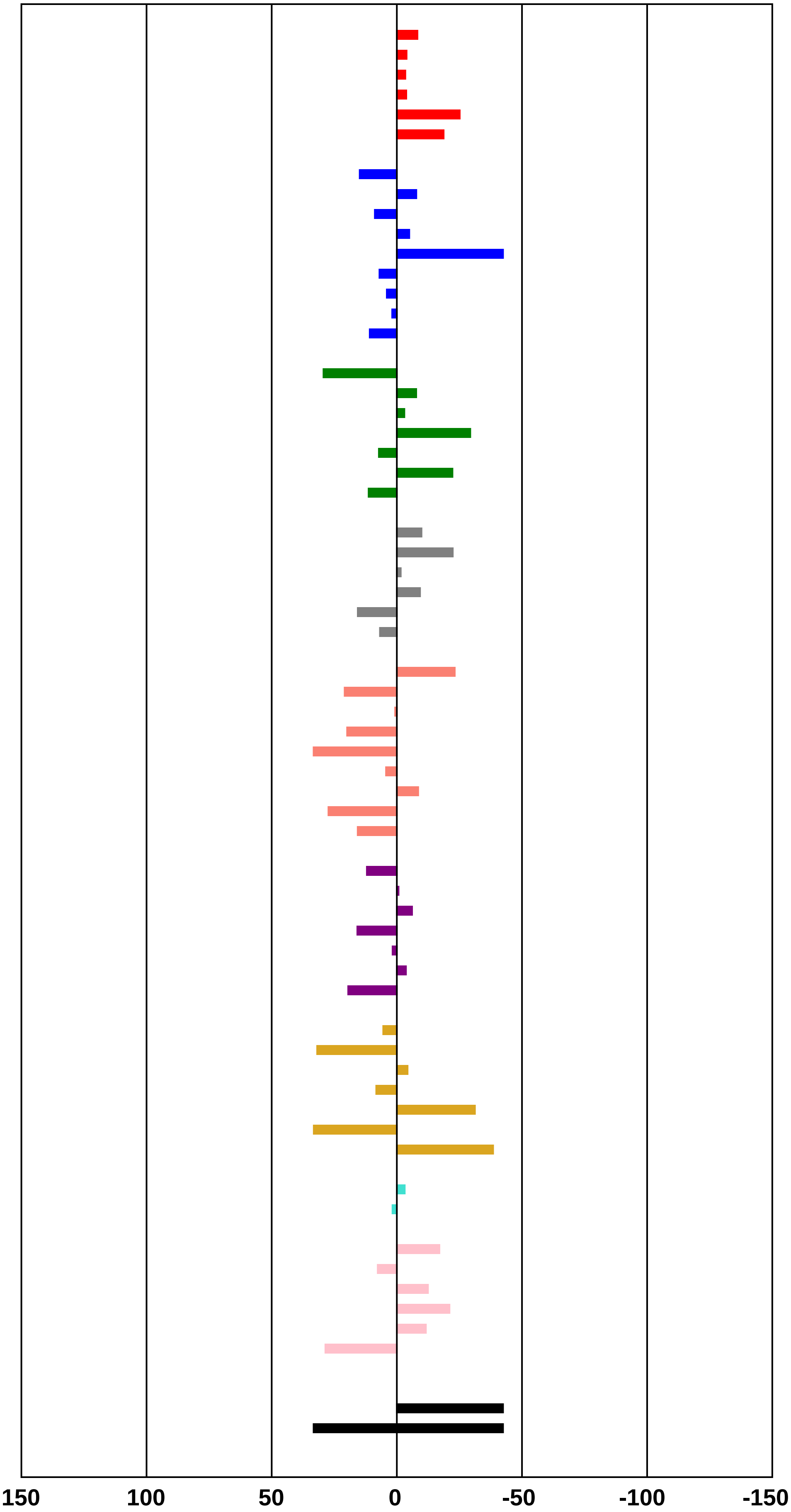

National Cancer Institute Developmental Therapeutics Program
Dose Response Curves

NSC: D - 835857 / 1 | SSPL: 1A9D | EXP. ID: 2205NS10

Report Date: June 19, 2022 | Test Date: May 02, 2022

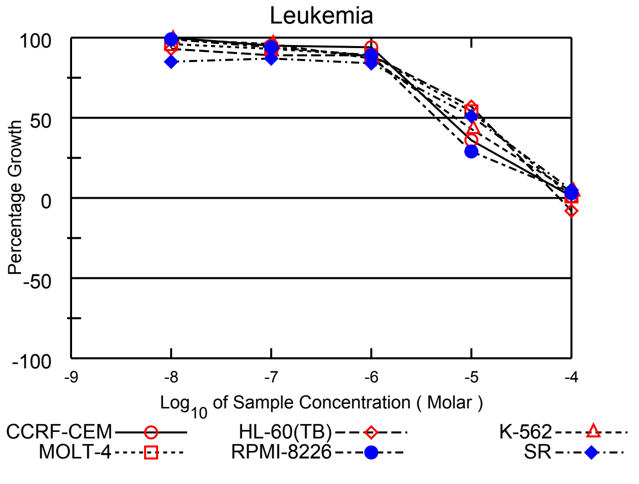

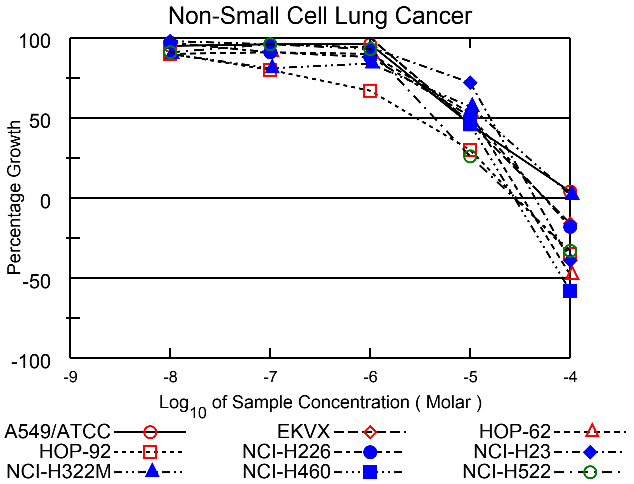

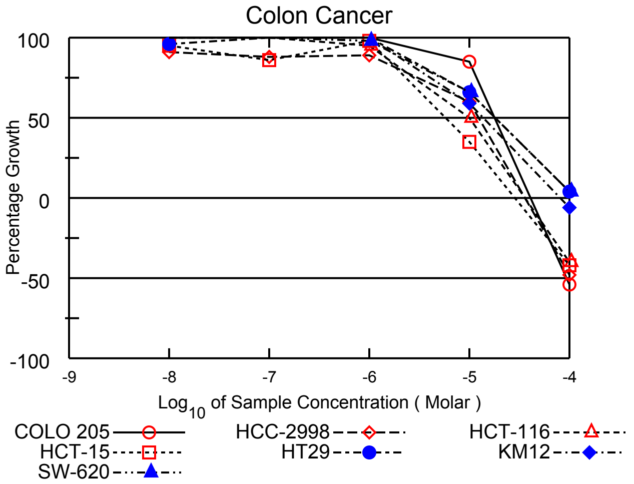

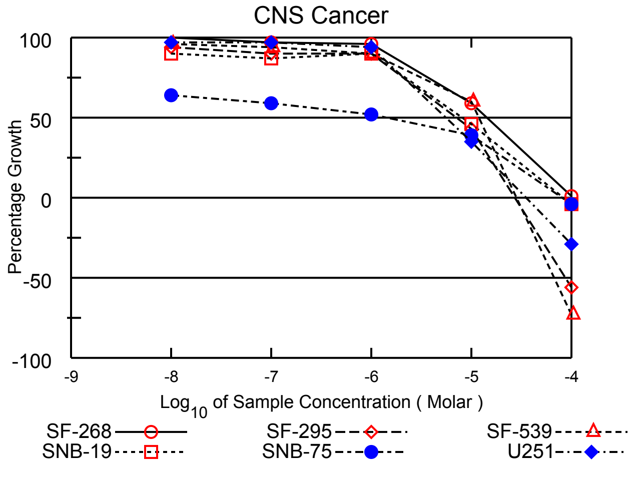

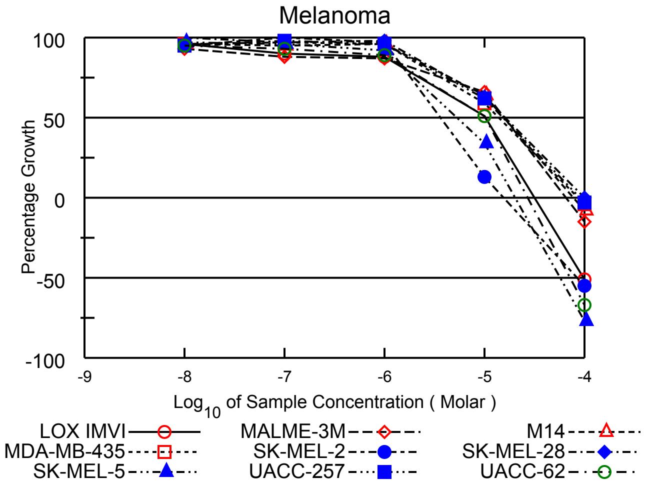

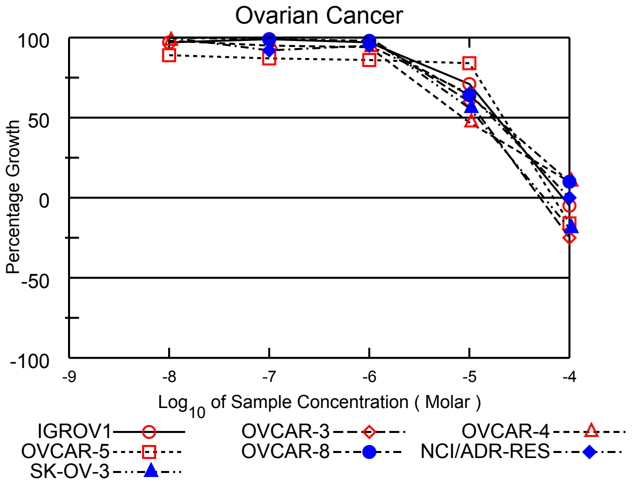

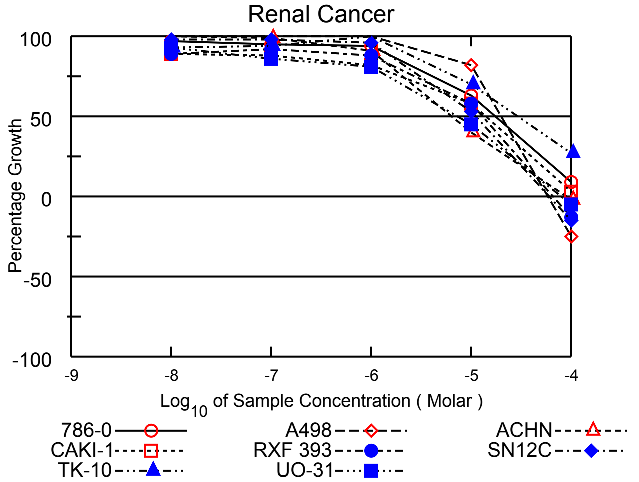

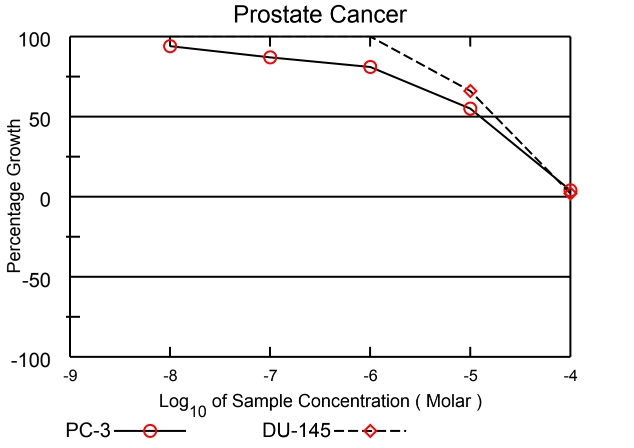

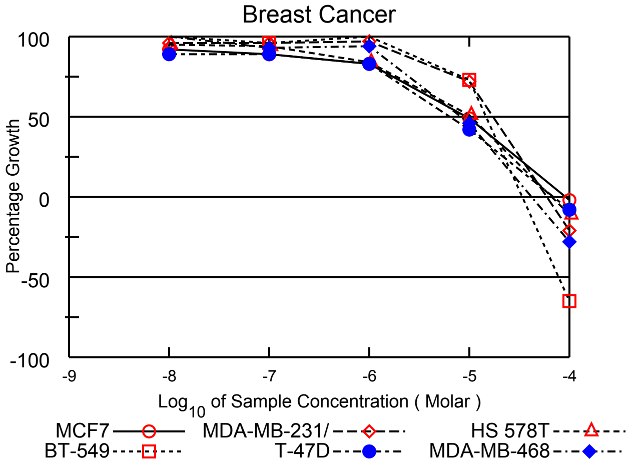

National Cancer Institute Developmental Therapeutics Program
In-Vitro Testing Results

NSC : D - 835857 / 1	Experiment ID : 2205NS10	Test Type : 08	Units : Molar
Report Date : June 19, 2022	Test Date : May 02, 2022	QNS :	MC :
COMI : 9d	Stain Reagent : SRB Dual-Pass Related	SSPL : 1A9D	

			Log10 Concentration													
	Time		Mean Optical Densities					Percent Growth								
Panel/Cell Line	Zero	Ctrl	-8.0	-7.0	-6.0	-5.0	-4.0	-8.0	-7.0	-6.0	-5.0	-4.0	GI50	TGI	LC50
Leukemia															
CCRF-CEM	0.555	2.544	2.539	2.449	2.430	1.268	0.573	100	95	94	36	1	5.72E-6	> 1.00E-4	> 1.00E-4
HL-60(TB)	0.599	2.910	2.755	2.660	2.653	1.921	0.551	93	89	89	57	-8	1.29E-5	7.54E-5	> 1.00E-4
K-562	0.195	2.363	2.352	2.276	2.073	1.137	0.273	99	96	87	43	4	7.05E-6	> 1.00E-4	> 1.00E-4
MOLT-4	0.517	2.669	2.582	2.523	2.434	1.671	0.528	96	93	89	54	1	1.17E-5	> 1.00E-4	> 1.00E-4
RPMI-8226	0.634	2.821	2.799	2.685	2.588	1.258	0.693	99	94	89	29	3	4.44E-6	> 1.00E-4	> 1.00E-4
SR	0.550	2.710	2.382	2.432	2.375	1.643	0.657	85	87	84	51	5	1.03E-5	> 1.00E-4	> 1.00E-4
Non-Small Cell Lung Cancer															
A549/ATCC	0.763	2.865	2.764	2.789	2.780	1.730	0.848	95	96	96	46	4	8.31E-6	> 1.00E-4	> 1.00E-4
EKVX	0.555	1.647	1.698	1.742	1.706	1.069	0.468	105	109	105	47	-16	8.89E-6	5.62E-5	> 1.00E-4
HOP-62	1.167	2.744	2.588	2.599	2.589	2.010	0.608	90	91	90	53	-48	1.08E-5	3.37E-5	> 1.00E-4
HOP-92	1.253	2.127	2.042	1.950	1.834	1.517	0.821	90	80	67	30	-35	2.85E-6	2.93E-5	> 1.00E-4
NCI-H226	0.905	1.913	1.885	1.826	1.793	1.415	0.740	97	91	88	51	-18	1.02E-5	5.42E-5	> 1.00E-4
NCI-H23	0.534	1.616	1.597	1.568	1.552	1.310	0.326	98	96	94	72	-39	1.57E-5	4.44E-5	> 1.00E-4
NCI-H322M	1.051	2.615	2.453	2.311	2.369	1.946	1.089	90	81	84	57	2	1.36E-5	> 1.00E-4	> 1.00E-4
NCI-H460	0.338	2.936	3.191	3.187	3.032	1.533	0.143	110	110	104	46	-58	8.52E-6	2.77E-5	8.40E-5
NCI-H522	1.493	2.969	2.830	2.906	2.868	1.871	0.994	91	96	93	26	-33	4.35E-6	2.71E-5	> 1.00E-4
Colon Cancer															
COLO 205	0.611	2.166	2.197	2.236	2.192	1.930	0.283	102	104	102	85	-54	1.78E-5	4.10E-5	9.41E-5
HCC-2998	0.733	2.480	2.323	2.273	2.285	1.781	0.383	91	88	89	60	-48	1.24E-5	3.60E-5	> 1.00E-4
HCT-116	0.204	2.147	2.199	2.151	2.043	1.184	0.123	103	100	95	50	-40	1.01E-5	3.61E-5	> 1.00E-4
HCT-15	0.243	1.661	1.594	1.466	1.628	0.745	0.141	95	86	98	35	-42	5.82E-6	2.87E-5	> 1.00E-4
HT29	0.296	1.730	1.678	1.829	1.790	1.236	0.354	96	107	104	66	4	1.79E-5	> 1.00E-4	> 1.00E-4
KM12	1.006	3.353	3.368	3.352	3.344	2.388	0.947	101	100	100	59	-6	1.37E-5	8.12E-5	> 1.00E-4
SW-620	0.417	2.599	2.698	2.624	2.559	1.866	0.494	105	101	98	66	4	1.82E-5	> 1.00E-4	> 1.00E-4
CNS Cancer															
SF-268	1.067	2.834	2.890	2.780	2.770	2.110	1.080	103	97	96	59	1	1.43E-5	> 1.00E-4	> 1.00E-4
SF-295	0.616	1.906	1.827	1.777	1.778	1.168	0.268	94	90	90	43	-56	7.04E-6	2.70E-5	8.60E-5
SF-539	0.768	2.442	2.376	2.339	2.269	1.777	0.208	96	94	90	60	-73	1.19E-5	2.83E-5	6.72E-5
SNB-19	1.188	2.590	2.452	2.406	2.450	1.832	1.136	90	87	90	46	-4	8.09E-6	8.17E-5	> 1.00E-4
SNB-75	1.757	3.018	2.564	2.496	2.409	2.252	1.685	64	59	52	39	-4	1.36E-6	8.03E-5	> 1.00E-4
U251	0.481	2.083	2.035	2.033	1.984	1.044	0.340	97	97	94	35	-29	5.58E-6	3.50E-5	> 1.00E-4
Melanoma															
LOX IMVI	0.428	2.755	2.662	2.527	2.474	1.616	0.210	96	90	88	51	-51	1.02E-5	3.16E-5	9.77E-5
MALME-3M	0.756	1.602	1.540	1.503	1.495	1.313	0.639	93	88	87	66	-15	1.56E-5	6.45E-5	> 1.00E-4
M14	0.445	1.900	1.843	1.835	1.849	1.371	0.410	96	95	96	64	-8	1.55E-5	7.74E-5	> 1.00E-4
MDA-MB-435	0.670	2.926	2.835	2.858	2.830	2.002	0.645	96	97	96	59	-4	1.39E-5	8.72E-5	> 1.00E-4
SK-MEL-2	1.579	3.042	2.966	3.042	3.004	1.772	0.705	95	100	97	13	-55	3.65E-6	1.56E-5	8.35E-5
SK-MEL-28	0.766	2.127	2.148	2.086	2.101	1.621	0.761	102	97	98	63	0	1.59E-5	9.74E-5	> 1.00E-4
SK-MEL-5	0.865	2.943	2.871	2.852	2.787	1.571	0.203	97	96	92	34	-77	5.32E-6	2.03E-5	5.75E-5
UACC-257	1.473	2.769	2.701	2.741	2.716	2.276	1.431	95	98	96	62	-3	1.53E-5	9.03E-5	> 1.00E-4
UACC-62	1.075	2.743	2.664	2.618	2.562	1.925	0.354	95	93	89	51	-67	1.02E-5	2.70E-5	7.16E-5
Ovarian Cancer															
IGROV1	1.370	3.066	3.012	3.044	3.013	2.581	1.308	97	99	97	71	-5	1.91E-5	8.71E-5	> 1.00E-4
OVCAR-3	0.989	2.633	2.757	2.678	2.634	1.977	0.747	107	103	100	60	-25	1.31E-5	5.13E-5	> 1.00E-4
OVCAR-4	1.278	2.763	2.729	2.682	2.670	1.982	1.434	98	95	94	47	10	8.77E-6	> 1.00E-4	> 1.00E-4
OVCAR-5	1.097	1.935	1.843	1.829	1.821	1.801	0.919	89	87	86	84	-16	2.18E-5	6.89E-5	> 1.00E-4
OVCAR-8	0.936	3.188	3.194	3.170	3.136	2.379	1.164	100	99	98	64	10	1.82E-5	> 1.00E-4	> 1.00E-4
NCI/ADR-RES	0.466	1.666	1.671	1.568	1.603	1.241	0.464	100	92	95	65	0	1.68E-5	9.85E-5	> 1.00E-4
SK-OV-3	1.282	2.159	2.207	2.256	2.227	1.769	1.036	105	111	108	56	-19	1.19E-5	5.53E-5	> 1.00E-4
Renal Cancer															
786-0	0.677	2.718	2.647	2.618	2.589	1.955	0.856	97	95	94	63	9	1.71E-5	> 1.00E-4	> 1.00E-4
A498	1.245	1.957	1.975	2.026	1.998	1.830	0.930	102	110	106	82	-25	1.99E-5	5.81E-5	> 1.00E-4
ACHN	0.645	2.533	2.549	2.520	2.364	1.406	0.634	101	99	91	40	-2	6.44E-6	9.11E-5	> 1.00E-4
CAKI-1	0.856	2.691	2.496	2.478	2.366	1.915	0.909	89	88	82	58	3	1.38E-5	> 1.00E-4	> 1.00E-4
RXF 393	0.799	1.291	1.234	1.250	1.232	1.086	0.696	89	92	88	58	-13	1.31E-5	6.59E-5	> 1.00E-4
SN12C	2.250	3.462	3.434	3.442	3.418	2.890	1.917	98	98	96	53	-15	1.10E-5	6.04E-5	> 1.00E-4
TK-10	1.140	2.064	2.002	2.004	2.111	1.791	1.385	93	94	105	70	27	2.93E-5	> 1.00E-4	> 1.00E-4
UO-31	1.112	3.017	2.888	2.755	2.662	1.971	1.051	93	86	81	45	-5	7.33E-6	7.79E-5	> 1.00E-4
Prostate Cancer															
PC-3	0.475	1.946	1.860	1.760	1.671	1.290	0.528	94	87	81	55	4	1.27E-5	> 1.00E-4	> 1.00E-4
DU-145	0.634	2.405	2.497	2.457	2.440	1.797	0.678	105	103	102	66	2	1.77E-5	> 1.00E-4	> 1.00E-4
Breast Cancer															
MCF7	0.420	2.107	1.966	1.918	1.815	1.249	0.410	92	89	83	49	-2	9.41E-6	8.99E-5	> 1.00E-4
MDA-MB-231/ATCC	0.754	1.395	1.368	1.370	1.377	1.214	0.598	96	96	97	72	-21	1.72E-5	5.97E-5	> 1.00E-4
HS 578T	1.607	2.525	2.484	2.468	2.378	2.072	1.436	95	94	84	51	-11	1.02E-5	6.70E-5	> 1.00E-4
BT-549	0.995	1.837	1.867	1.807	1.851	1.606	0.351	104	96	102	73	-65	1.46E-5	3.38E-5	7.81E-5
T-47D	1.019	2.367	2.219	2.222	2.138	1.581	0.941	89	89	83	42	-8	6.29E-6	7.00E-5	> 1.00E-4
MDA-MB-468	0.840	1.693	1.690	1.635	1.643	1.233	0.609	100	93	94	46	-28	8.29E-6	4.23E-5	> 1.00E-4

National Cancer Institute Developmental Therapeutics Program

Mean Graphs

NSC : D - 835857/1 | Units :Molar | SSPL :1A9D | EXP. ID :2205NS10

Report Date :June 19, 2022 | Test Date :May 02, 2022

Panel/Cell Line	Log_{10}GI50	Log_{10}TGI	Log_{10}LC50
**Leukemia**			
CCRF-CEM	-5.24	> -4.00	> -4.00
HL-60(TB)	-4.89	-4.12	> -4.00
K-562	-5.15	> -4.00	> -4.00
MOLT-4	-4.93	> -4.00	> -4.00
RPMI-8226	-5.35	> -4.00	> -4.00
SR	-4.99	> -4.00	> -4.00
**Non-Small Cell Lung Cancer**			
A549/ATCC	-5.08	> -4.00	> -4.00
EKVX	-5.05	-4.25	> -4.00
HOP-62	-4.97	-4.47	> -4.00
HOP-92	-5.55	-4.53	> -4.00
NCI-H226	-4.99	-4.27	> -4.00
NCI-H23	-4.80	-4.35	> -4.00
NCI-H322M	-4.87	> -4.00	> -4.00
NCI-H460	-5.07	-4.56	-4.08
NCI-H522	-5.36	-4.57	> -4.00
**Colon Cancer**			
COLO 205	-4.75	-4.39	-4.03
HCC-2998	-4.91	-4.44	> -4.00
HCT-116	-5.00	-4.44	> -4.00
HCT-15	-5.23	-4.54	> -4.00
HT29	-4.75	> -4.00	> -4.00
KM12	-4.86	-4.09	> -4.00
SW-620	-4.74	> -4.00	> -4.00
**CNS Cancer**			
SF-268	-4.84	> -4.00	> -4.00
SF-295	-5.15	-4.57	-4.07
SF-539	-4.92	-4.55	-4.17
SNB-19	-5.09	-4.09	> -4.00
SNB-75	-5.87	-4.10	> -4.00
U251	-5.25	-4.46	> -4.00
**Melanoma**			
LOX IMVI	-4.99	-4.50	-4.01
MALME-3M	-4.81	-4.19	> -4.00
M14	-4.81	-4.11	> -4.00
MDA-MB-435	-4.86	-4.06	> -4.00
SK-MEL-2	-5.44	-4.81	-4.08
SK-MEL-28	-4.80	-4.01	> -4.00
SK-MEL-5	-5.27	-4.69	-4.24
UACC-257	-4.82	-4.04	> -4.00
UACC-62	-4.99	-4.57	-4.14
**Ovarian Cancer**			
IGROV1	-4.72	-4.06	> -4.00
OVCAR-3	-4.88	-4.29	> -4.00
OVCAR-4	-5.06	> -4.00	> -4.00
OVCAR-5	-4.66	-4.16	> -4.00
OVCAR-8	-4.74	> -4.00	> -4.00
NCI/ADR-RES	-4.78	-4.01	> -4.00
SK-OV-3	-4.93	-4.26	> -4.00
**Renal Cancer**			
786-0	-4.77	> -4.00	> -4.00
A498	-4.70	-4.24	> -4.00
ACHN	-5.19	-4.04	> -4.00
CAKI-1	-4.86	> -4.00	> -4.00
RXF 393	-4.88	-4.18	> -4.00
SN12C	-4.96	-4.22	> -4.00
TK-10	-4.53	> -4.00	> -4.00
UO-31	-5.14	-4.11	> -4.00
**Prostate Cancer**			
PC-3	-4.90	> -4.00	> -4.00
DU-145	-4.75	> -4.00	> -4.00
**Breast Cancer**			
MCF7	-5.03	-4.05	> -4.00
MDA-MB-231/ATCC	-4.76	-4.22	> -4.00
HS 578T	-4.99	-4.17	> -4.00
BT-549	-4.84	-4.47	-4.11
T-47D	-5.20	-4.16	> -4.00
MDA-MB-468	-5.08	-4.37	> -4.00
_MID	-4.98	-4.21	-4.02
Delta	0.89	0.6	0.22
Range	1.34	0.81	0.24

GI50 | TGI | LC50

+3 +2 +1 0 -1 -2 -3

National Cancer Institute Developmental Therapeutics Program Dose Response Curves	NSC : D - 835857/1	SSPL:1A9D	EXP. ID:2205NS10
	Report Date:June 19, 2022	Test Date:May 02, 2022	

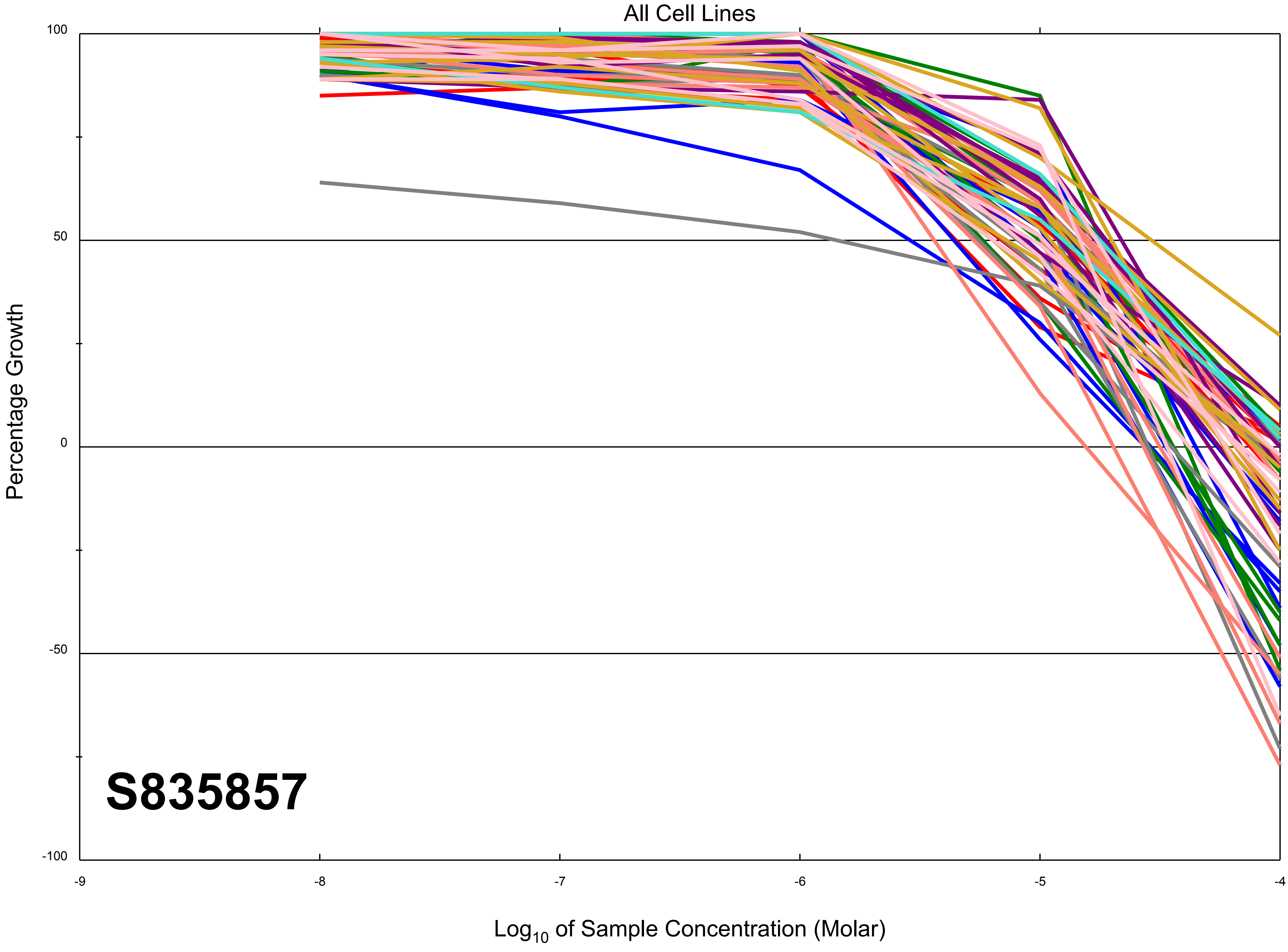

Supplement: Supplemental Material [file IENZ_A_2191163_SM5021.pdf]
